# cloneRate: fast estimation of single-cell clonal dynamics using coalescent theory

**DOI:** 10.1093/bioinformatics/btad561

**Published:** 2023-09-12

**Authors:** Brian Johnson, Yubo Shuai, Jason Schweinsberg, Kit Curtius

**Affiliations:** Division of Biomedical Informatics, Department of Medicine, University of California San Diego, La Jolla, CA 92093, United States; Department of Mathematics, University of California San Diego, La Jolla, CA 92093, United States; Department of Mathematics, University of California San Diego, La Jolla, CA 92093, United States; Division of Biomedical Informatics, Department of Medicine, University of California San Diego, La Jolla, CA 92093, United States; Moores Cancer Center, University of California San Diego, La Jolla, CA 92093, United States; VA San Diego Healthcare System, San Diego, CA 92161, United States

## Abstract

**Motivation:**

While evolutionary approaches to medicine show promise, measuring evolution itself is difficult due to experimental constraints and the dynamic nature of body systems. In cancer evolution, continuous observation of clonal architecture is impossible, and longitudinal samples from multiple timepoints are rare. Increasingly available DNA sequencing datasets at single-cell resolution enable the reconstruction of past evolution using mutational history, allowing for a better understanding of dynamics prior to detectable disease. There is an unmet need for an accurate, fast, and easy-to-use method to quantify clone growth dynamics from these datasets.

**Results:**

We derived methods based on coalescent theory for estimating the net growth rate of clones using either reconstructed phylogenies or the number of shared mutations. We applied and validated our analytical methods for estimating the net growth rate of clones, eliminating the need for complex simulations used in previous methods. When applied to hematopoietic data, we show that our estimates may have broad applications to improve mechanistic understanding and prognostic ability. Compared to clones with a single or unknown driver mutation, clones with multiple drivers have significantly increased growth rates (median 0.94 versus 0.25 per year; *P* = $1.6\times 10^{-6}$). Further, stratifying patients with a myeloproliferative neoplasm (MPN) by the growth rate of their fittest clone shows that higher growth rates are associated with shorter time to MPN diagnosis (median 13.9 versus 26.4 months; *P* = 0.0026).

**Availability and implementation:**

We developed a publicly available R package, cloneRate, to implement our methods (Package website: https://bdj34.github.io/cloneRate/). Source code: https://github.com/bdj34/cloneRate/.

## 1 Introduction

Clonal expansions of cells that acquire certain mutations post-conception are a direct result of somatic evolution and are prevalent across the human body (Jonason *et al.* 1996, Jaiswal *et al.* 2014, Martincorena *et al.* 2015, 2018, Suda *et al.* 2018, Lee-Six *et al.* 2019, Yokoyama *et al.* 2019). By estimating the timing of clone initiation and subsequent growth rates of clones, we can characterize evolutionary mechanisms that underlie aging (Steensma and Ebert 2020) and malignant progression (Martinez *et al.* 2016, Scott and Marusyk 2017, Curtius *et al.* 2018, Gerstung *et al.* 2020). In blood, for example, this evolutionary process is known as ‘clonal hematopoiesis’ and has been associated with many aging-related disorders such as anemia (van Zeventer *et al.* 2020), impaired immunity (Geiger *et al.* 2013, Schenz *et al.* 2022), and cardiovascular disease (Sano *et al.* 2022, Tall and Fuster 2022), as well as progression to hematologic cancers (Jaiswal *et al.* 2014, Gillis *et al.* 2017, Warren and Link 2020). Previous analyses found that somatic mutations conferring higher fitness, measured by clonal growth rate, lead to a higher risk of malignant transformation (Watson *et al.* 2020, Fabre *et al.* 2022). However, validated methods for measuring these important evolutionary parameters, which can vary from patient to patient, remain limited. Fast, accurate estimates of the underlying clonal dynamics using genomic data could serve to improve prognostic ability and ultimately lead to better patient outcomes.

Recent whole genome single-cell sequencing experiments in blood (Van Egeren *et al.* 2021, Fabre *et al.* 2022, Mitchell *et al.* 2022, Williams *et al.* 2022) allow for phylogenetic reconstruction of the ancestral relationships among cells. Information on the growth dynamics of individual clones is contained in the phylogeny of sampled cells from a population (Stadler *et al.* 2021). Classical phylodynamics approaches to estimate population size trajectories depend on Kingman’s coalescent (Kingman 1982) and its subsequent generalization to variable population size (Slatkin and Hudson 1991, Griffiths and Tavaré 1994, 1998). With this approach, ancestral lineages are assumed to merge at a rate which is inversely proportional to the population size, and exact computations are sometimes feasible (Polanski and Kimmel 2003). The coalescent, with the assumption of an underlying deterministic population growth model such as logistic growth, provides the basis for clone growth rate estimation using the *phylodyn* R package (Karcher *et al.* 2017, Van Egeren *et al.* 2021, Fabre *et al.* 2022), as well as a method called Phylofit (Mitchell *et al.* 2022, Williams *et al.* 2022). Alternatively, the package BEAST 2 (Bouckaert *et al.* 2019) enables phylodynamic inference either by using the coalescent method or by modeling the population as a birth–death process, which allows the population size to vary stochastically without relying on coalescent approximations (Stadler 2009, Boskova *et al.* 2014). Due to the lack of an analytical solution for confidence intervals, these previous approaches estimate the growth rate using Markov chain Monte Carlo (MCMC), integrated nested Laplace approximations (INLA), or approximate Bayesian computation (ABC). Here we introduce new methods for estimating the net growth rate of a continuous time, supercritical birth–death process. The birth–death process is consistent with a cellular model of symmetric division (birth) and death or differentiation, and our methods remain valid for other models of clonal expansion that begin with an exponential growth phase (e.g. logistic, Gompertzian, etc.) in the context of hematopoietic data. Importantly, our methods require few assumptions and do not depend on computationally expensive simulation, allowing for near instantaneous estimation of the growth rate and its confidence intervals.

Our methods build on the mathematical work of Harris *et al.* (2020) and Lambert (2018), who recently discovered a relatively simple way to describe the ‘exact’ genealogy of a sample of size *n* at time *T* from a birth–death process. Using Lambert’s construction, we derive an approximation to the genealogy when *T* and *n* are large and use this approximation to obtain a maximum likelihood estimate of the net growth rate of a clone. We prove a limit theorem which gives the asymptotic distribution for the total lengths of the internal and external branches in the phylogenetic tree. The asymptotic distribution of the total internal branch length leads to a second method for estimating the net growth rate. This also allows us to estimate the net growth rate directly from the number of internal or shared mutations, those which are inherited by more than one of the sampled cells. Additionally, we provide an estimate for clone age which is applicable when the growth rate is known and the mutation rate is unknown.

Recent single-cell sequencing datasets (Van Egeren *et al.* 2021, Fabre *et al.* 2022, Mitchell *et al.* 2022, Williams *et al.* 2022) have generated novel insights about the nature of clones in the blood, identifying high risk mutations and revealing that clonal expansions with known drivers are present decades before symptoms appear, if they appear at all (Williams *et al.* 2022). Applying our methods to these datasets generates additional insights on the early growth rates of clones, which appear to be clinically relevant. We validate our estimates with longitudinal data and show that our methods contribute to a better understanding of the overall trajectory of the population size of a clone, refining previous estimates and further advancing our understanding of hematopoiesis, aging and cancer initiation.

## 2 Materials and methods

We derived new mathematical estimates for evolutionary parameters (e.g. growth rate of a clone) when analyzing single-cell-derived DNA sequencing data from a ‘sample’ of the clone. A sample is a random subset of the total cells in the clone, as is commonly available in a realistic single-cell dataset. In the blood datasets analyzed below, samples of unique clones range from 4 to 109 cells (Van Egeren *et al.* 2021, Fabre *et al.* 2022, Mitchell *et al.* 2022, Williams *et al.* 2022), while total clone size can hypothetically be as large as the total number of hematopoietic stem cells (HSCs) in the human body [estimates range from 25 000–300 000 (Lee-Six *et al.* 2018, Watson *et al.* 2020, Moeller *et al.* 2022)]. Analysis of coalescence times then requires explicit consideration of the size of the sample, and new theoretical results were needed to obtain analytical growth rate estimates in this setting. In this section, we provide our estimates for clonal growth parameters under a wide range of applicable modeling assumptions, then apply them to simulated and real data. We also compare our results to those produced using Phylofit, a recent coalescent-based MCMC approach (Williams *et al.* 2022), and a birth–death MCMC approach introduced by Stadler (2009).

### 2.1 Mathematical models for estimating clonal growth

First, we describe the biological rationale for inferring the growth rate from a genealogical tree. All cells sampled from the same clone progeny will have a common ancestor dating back to the clone’s origin, i.e. when the first cell acquired the identifying mutation leading to clonal expansion. Any two sampled cells may have a more recent common ancestor, and the most recent time at which the two cells have a common ancestor is called the ‘coalescence time’ for these cells. In a sample of *n* cells, there will be *n* *−* 1 distinct coalescence times. For larger populations, it is less likely that any two sampled cells will have a recent common ancestor. Therefore, a faster growing (larger) population should have older common ancestors and a slower growing (smaller) population should have more recent common ancestors. Because the probability of a shared ancestor is dependent on the total clone size, the distribution of coalescence times provides information on the clone size trajectory, and here we use it to infer the early growth rate of the clone, *r*.

**Figure 1.**
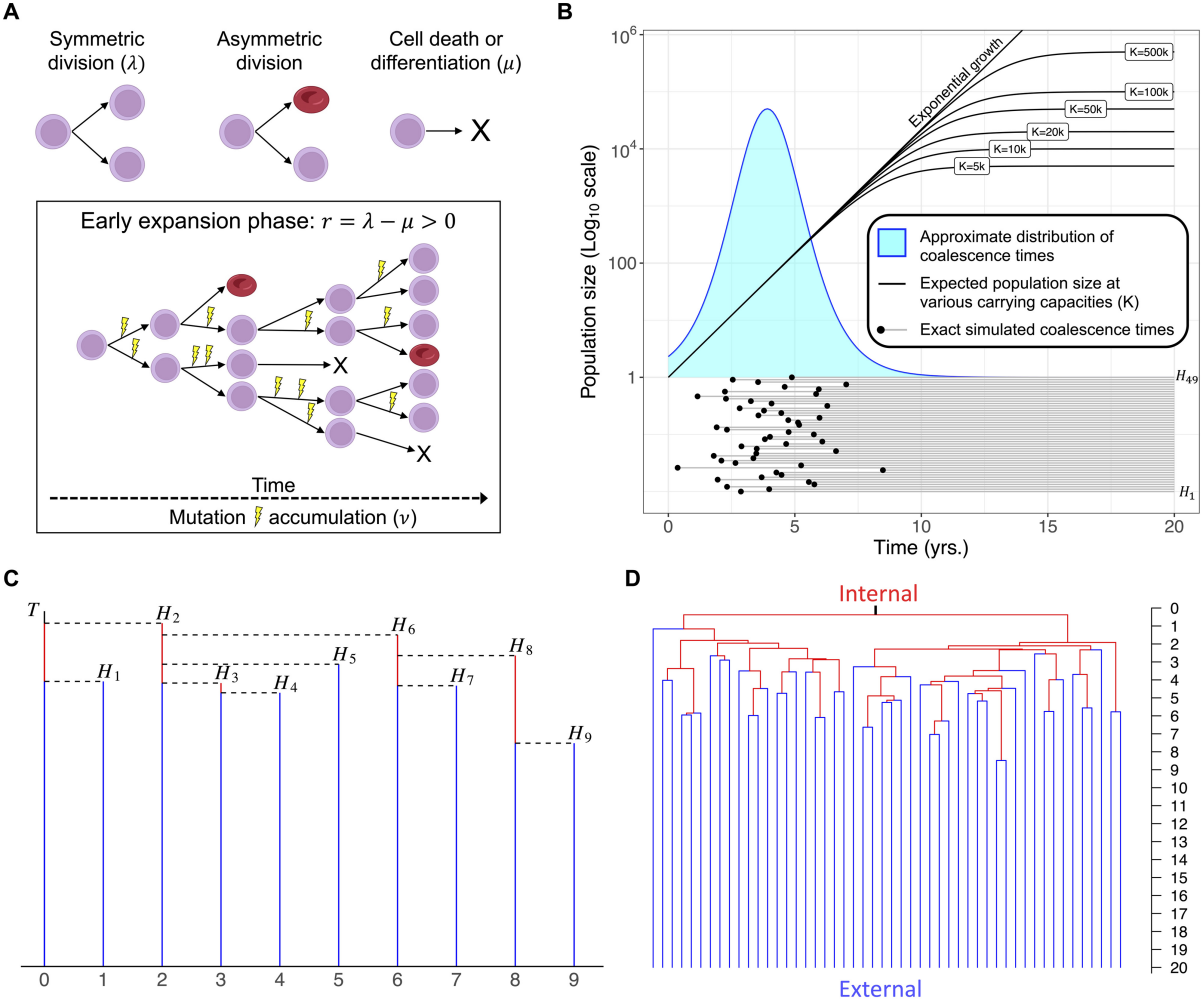
Model schematic and coalescent results. (A) Stem cells undergo symmetric division at rate *λ*, increasing the population of stem cells by 1. Asymmetric division does not affect the population size or phylogeny except to introduce mutations. Cell death (or differentiation) occurs at rate *μ*, which removes the cell’s inherited history from the phylogeny and decreases the population size by 1. Our methods seek only to estimate the growth rate during the expansion phase of a clone, when the rate of symmetric division is greater than the rate of cell death (r=λ−μ>0) and both rates are assumed to be constant during this phase. Mutations, which can occur at or between divisions, are assumed to accumulate linearly with time at rate *ν*. (B) The approximate distribution of coalescence times for *n *=* *50 cells is plotted above one example of n−1=49 coalescence times drawn from the exact distribution of coalescence times for a birth–death process. The expected population size assuming logistic growth with different carrying capacities shows that most coalescence events occur at smaller population sizes, when the growth trajectory is still approximately exponential. Other parameters: *r *=* *1, (λ=1.5, μ=0.5), *T *=* *20. Note that a sampling time *T *<* *10 years would artificially affect the distribution of coalescence times, introducing bias. (C) Method overview: Reconstruction of a genealogical tree using the coalescent point process (CPP) can be done by first adding a vertical line of length *T*, and then adding successive vertical lines representing the coalescence times (*H_i_*). The coalescence times are drawn i.i.d. from the distribution defined in Supplementary Section S1.1 and are then connected via horizontal lines to form the ultrametric tree. (D) Tree reconstructed by randomly merging lineages with coalescence times from (B), which is statistically equivalent to using the coalescent point process.

To connect growth rates to a genealogical tree, we consider the following birth–death process. We assume each cell divides symmetrically at rate *λ* and dies or differentiates at rate *μ*, acquiring mutations through time at rate *ν*. We wish to estimate r=λ−μ, the net growth rate (see Fig. 1A). The data consists of a sample of *n* cells from the clone (of total size = *N* cells) at a clone age *T*. We assume that *r* is positive and constant during the expansion phase of the clone. We also assume the sample size *n* is much smaller than the total clone size *N*, which is usually valid for single-cell-derived datasets described herein that typically have most coalescence events occurring shortly after clone initiation (i.e. star-shaped genealogies) (Van Egeren *et al.* 2021, Fabre *et al.* 2022, Mitchell *et al.* 2022, Williams *et al.* 2022). Although our mathematical results are proved for this simple birth–death process, they can be applied to a larger class of models that describe the expansion of clones in blood with an early exponential growth phase [e.g. logistic growth (Fabre *et al.* 2022, Williams *et al.* 2022), purely exponential growth (Watson *et al.* 2020), Wright–Fisher with selection (Van Egeren *et al.* 2021), etc.]. Our results will be valid when observed coalescence times are not impacted by changing growth rates that may occur after the initial expansion phase (see Fig. 1B), and when the time at sampling, *T*, does not bias the coalescence times.

### 2.2 Approximating genealogy using a coalescent point process

A recent elegant method by Lambert for computing the exact genealogy of a sample of size *n* at time *T* from a birth–death process is described in Supplementary Section S1.1 (Lambert 2018). Because we are mostly interested in the case when the clone age at sampling *T* and the sample size *n* are large, we can obtain a useful approximation by letting T→∞ and then n→∞ in Lambert’s construction. This leads to the following simpler method for approximating the coalescence times $H_{1},\ldots ,H_{n-1}$, which provides the foundation for our estimates of the net growth rate:

1. Let *W* have an exponential distribution with mean 1.
2. Let $U_{1},U_{2},\ldots ,U_{n-1}$ be independent and identically distributed (i.i.d.) random variables having the logistic distribution, which is a symmetric distribution on the real line with density given by
  $$f_{U_{i}}(u)=\frac{e^{u}}{{\left(1+e^{u}\right)}^{2}}.$$
3. Let
  $$H_{i}=T-\frac{1}{r}(\text{log}(1/W)+\text{log}\;n+U_{i}).$$

For mathematical details of the derivation, see Supplementary Section S2.1. Note that we model the coalescence times as i.i.d. random variables having a logistic distribution, plus a random shift which accounts for the randomness in the initial growth of the branching process. Ignatieva *et al.* (2020) showed using a different method involving a random time change that the coalescence times can be approximated well by i.i.d. logistic random variables.

Once the *H_i_* have been determined, we can construct the genealogical tree by randomly merging two lineages at each coalescence time, or by using the coalescent point process as shown in Fig. 1C. To understand the formula for *H_i_*, note that in a supercritical branching process (*r *>* *0), the individuals sampled at time *T* are likely to have been descended from different ancestors fairly close to time zero, when the clone began expanding. That is, the genealogical tree will be nearly star-shaped, with most coalescence occurring near time 0. Note that the expected population size of the clone at time *t* is *e^rt^*. Considering the case when λ=r and *μ *= 0, the size of the population after a large time *t* can therefore be approximated by $We^{rt}$, where *W* has an exponential distribution with mean 1. This expression equals *n* when $t=\frac{1}{r}(\text{log}(1/W)+\text{log}\;n)$. We expect most lineages to coalesce when the size of the population is comparable to *n*, which is why the coalescence times *H_i_* are close to $T-\frac{1}{r}(\text{log}(1/W)+\text{log}\;n)$.

### 2.3 Estimating growth rate of a clone

If we can reconstruct the full genealogical tree from data, then we have estimates for the *n* *−* 1 coalescence times $H_{1},\ldots ,H_{n-1}$. From the discussion above, we can write

(1) $$\begin{array}{c} H_{i}=a+bU_{i},a=T-\frac{1}{r}(\text{log}(1/W)+\text{log}\;n),b=\frac{1}{r}, \end{array}$$

where the random variables $U_{1},\ldots ,U_{n}$ are i.i.d. and have a logistic distribution. Note that we can write b=1/r instead of b=−1/r because the logistic distribution is symmetric. We can therefore estimate the growth rate *r* by estimating the parameter *b*. We introduce here three methods. Alongside the results below, we also created an R package *cloneRate* for implementing growth rate estimation on novel user input data.

#### 2.3.1 Growth rate estimation using maximum likelihood

Maximum likelihood can be used to estimate *b* from $H_{1},\ldots ,H_{n-1}$. Because the maximum likelihood estimate does not have a closed form expression, it must be found using numerical methods. We computed the maximum likelihood estimate $\hat{b}$ in R using the Nelder–Mead method (Nelder and Mead 1965). From Equation (1), we can estimate *r* by

(2) $$\hat{r}=1/\hat{b}.$$

Let 0<α<1. A 100(1−α)% confidence interval (CI) for *r* is

(3) $$[\hat{r}(1-\frac{cz_{\alpha /2}}{\sqrt{n}}),\;\hat{r}(1+\frac{cz_{\alpha /2}}{\sqrt{n}})],\;c=\frac{3}{\sqrt{3+\pi^{2}}},$$

where $z_{\alpha /2}$ is the number such that if *Z* has a standard normal distribution, then $P(Z>z_{\alpha /2})=\alpha /2$. Note that c≈.836, which we use to compare to the confidence intervals of the following estimate based on internal branch lengths. See Supplementary Section S3.1 for CI derivation.

#### 2.3.2 Growth rate estimation using internal branch lengths

If we are able to reconstruct the full tree, then we know the internal branch lengths $L_{n}^{in}$ (e.g. sum of the lengths of red branches in Fig. 1D). By Theorem 1 in Supplementary Section S1.2, the distribution of $L_{n}^{in}$ is approximately normal with mean *n*/*r* and variance $n/r^{2}$. Therefore, we can estimate the growth rate by

(4) $$\hat{r}=\frac{n}{L_{n}^{in}}$$

and we obtain an asymptotically valid 100(1−α)% confidence interval for *r* by

(5) $$[\hat{r}(1-\frac{z_{\alpha /2}}{\sqrt{n}}),\;\hat{r}(1+\frac{z_{\alpha /2}}{\sqrt{n}})].$$

This estimate based on the internal branch lengths can be compared directly to the maximum likelihood estimate, as both methods take a time-based ultrametric tree as input. If the coalescence times are accurate, then considering only the internal branch lengths discards relevant information and one would expect the maximum likelihood estimate to perform better. The confidence bounds of the internal lengths estimate reflect this, as the confidence bounds of the internal lengths method in Equation (5) are identical in form to the confidence bounds for maximum likelihood in Equation (3), except that the internal lengths effectively has *c *=* *1. Because c=1>0.836, the internal lengths method has wider confidence intervals. See Supplementary Section S3.2 for CI derivation.

When reconstructing a time-based ultrametric tree from mutations, there will be some randomness inherent in estimating the edge lengths, due to the Poissonian accumulation of mutations. Because the above methods use the time-based tree as input, neither accounts for this uncertainty. The following section uses the ideas presented here to estimate the net growth rate directly from the observed mutations, providing confidence bounds which account for the randomness of mutation accumulation.

#### 2.3.3 Growth rate estimation using shared mutations rather than full tree

If we can estimate the mutation rate *ν* during the expansion phase of the clone, then we can also estimate the growth rate directly from the number of shared mutations, defined as those mutations present in more than 1 but not all of the *n* sampled cells. The key idea is that there will be more shared mutations when the growth rate is smaller and fewer when the growth rate is larger. As shown in Supplementary Section S1.3, the distribution of the number of shared mutations *M^in^* is approximately normal with mean nν/r and variance $\sigma^{2}=n(\nu /r+\nu^{2}/r^{2})$. Therefore, if the mutation rate *ν* is known, we can estimate the growth rate by

(6) $$\hat{r}=\frac{n\nu }{M^{in}}.$$

An asymptotically valid 100(1−α)% confidence interval for *r* is given by

(7) $$[\hat{r}(1-\frac{z_{\alpha /2}}{\sqrt{n}}\sqrt{1+\frac{n}{M_{n}^{in}}}),\;\hat{r}(1+\frac{z_{\alpha /2}}{\sqrt{n}}\sqrt{1+\frac{n}{M_{n}^{in}}})].$$

Accounting for Poissonian fluctuations in the observed number of shared mutations leads to confidence bounds slightly wider than those from the internal lengths method [Equation (5)]. See Supplementary Section S3.3 for CI derivation.

## 3 Results

### 3.1 Estimation performance on simulated data

To verify the performance of our methods, we generated trees using the exact genealogy reconstruction discussed in full detail in Supplementary Section S1.1. Recent work by Lambert (2018) allows for instantaneous generation of the exact genealogy of a sample from a supercritical birth–death process, removing the need for complex simulations [such as those employed by the stochastic simulator MASTER (Vaughan and Drummond 2013) in BEAST 2 (Bouckaert *et al.* 2019)] for many population genetics and coalescent applications. We briefly describe this process here and note that the tree generator is available in *cloneRate*. For a sample of size *n* at time *T* from a clone expanding with birth rate *λ* and death rate *μ*, *n − *1 exact coalescence times are drawn using the process described in Supplementary Section S1.1. An example of a set of 49 coalescence times for a sample of 50 cells is shown in Fig. 1B. Given the coalescence times $H_{i},\ldots ,H_{n-1}$, the coalescent point process can be used to quickly reconstruct the genealogy. To reconstruct the tree from the coalescence times, we begin by drawing a vertical line of height *T*. We then draw vertical lines of heights $H_{1},\ldots ,H_{n-1}$ and, at the top of each vertical line, draw a horizontal line to the left, stopping when it hits a vertical branch. The resulting tree is ultrametric, meaning that the root to tip distance is the same for all tips. Figure 1C shows a schematic example of a tree generated with 10 tips from 9 coalescence times, and Fig. 1D shows the tree constructed using the coalescence times in Fig. 1B.

Then, applying our methods to these reconstructed trees gives a distribution of estimates which allows for benchmarking since we know the “true” growth rate in the simulated data. We compared the performance of our methods to the MCMC-based approach, Phylofit, introduced by Williams *et al.* (2022). We did not compare to the performance of their ABC-based estimates, but note that the authors show a strong correlation between estimates from Phylofit and the ABC-based method (correlation coefficient *r* = 0.96) (Williams *et al.* 2022). We also compared our methods to another MCMC approach based on the birth-death model using the likelihood given in Equation (5) by Stadler (2009). Whereas Phylofit is based on Kingman’s coalescent assuming logistic population growth, the method based on Stadler’s work models the population as a birth–death process, and assumes each individual is sampled with some fixed probability *ρ*. Our methods also use a birth–death process but instead assume a fixed sample size *n*, allowing us to obtain analytical approximations when the sample size is much smaller than the population size at the time of sampling.

#### 3.1.1 Performance across varying sample size *n* and growth rate *r*

**Figure 2.**
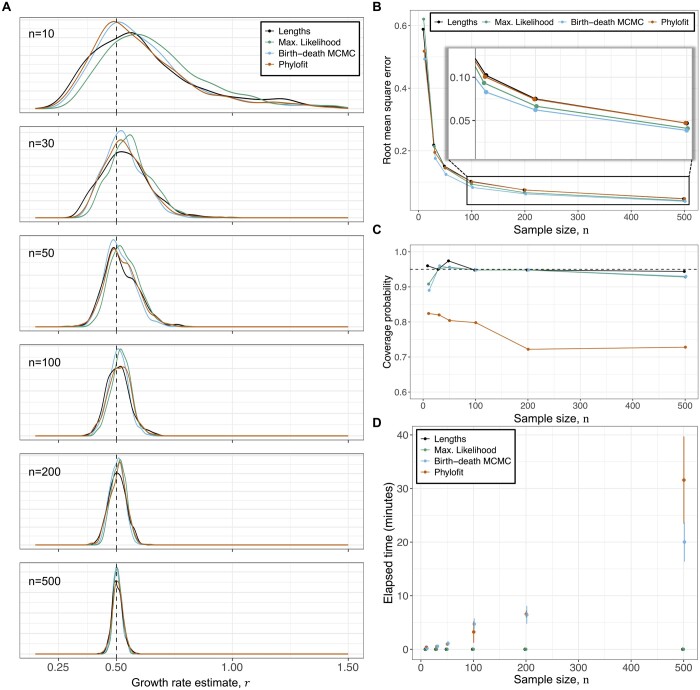
Performance and number of samples, *n*. (A) Distribution of estimates from our methods using Equation (2) (Max. Likelihood) and Equation (4) (Lengths), Phylofit, and the birth–death MCMC on 500 simulated ultrametric trees for each *n* value, where *n* is the number of sampled cells. Simulated trees were generated assuming a continuous time birth–death branching process with *r *=* *0.5 per unit time and *T *=* *40 time units, where time units are arbitrary (e.g. years). Birth rate *λ* was sampled from a uniform distribution on [0.5,1.5] and death rate μ=λ−r. (B) Root mean square error for each method from simulated data shown in (A) illustrates improved performance with number of samples. MCMC methods are most accurate for small *n*, while the birth–death MCMC and maximum likelihood perform best for large *n*. (C) Coverage of 95% confidence intervals methods based on simulations in (A), measured as the fraction of simulations where the true growth rate falls within the estimated confidence intervals. (D) Runtime (mean +/− st. dev.) of various methods of estimating net growth rate shows that while the MCMC-based methods scale with the number of samples, *n*, our methods run effectively instantaneously for any tree size.

We found similar performance using our methods compared to the MCMC methods. As shown in Fig. 2A and B, both MCMC methods appear to perform slightly better for small sample size *n*, while our maximum likelihood method outperforms Phylofit for n≥100. Of our two analytical methods, maximum likelihood has the lower root mean square error for larger values of *n* and converges to the birth–death MCMC as *n* becomes large (see Fig. 2B). When the sample size *n* is too low, our approximation of the distribution of coalescence times, which is valid as n→∞, no longer accurately describes the population. Intuitively, a smaller sample provides less information available to make an accurate estimate of the growth rate. As such, performance deteriorates and the confidence intervals of our estimates expand with decreasing *n* (see Equations (3), (5), and (7)). We use a cutoff sample size of *n *=* *10 for each clone when applying to real data below, but note that this cutoff depends on the desired accuracy of the estimate.

The confidence intervals for our methods are approximately accurate, as shown in Fig. 2C. We evaluate the accuracy of the confidence intervals using coverage probability, which denotes the fraction of simulations where the true growth rate falls within the estimated confidence interval (in our case, 95% confidence intervals). We note that the maximum likelihood confidence intervals may be slightly too narrow for small *n* because the variance estimate is based on the asymptotic Cramer–Rao bound (Antle *et al.* 1970). Alternatively, the 95% highest posterior densities (HPD) for Phylofit are consistently too narrow, where <80% of coverage is typically observed for growth rate estimates (Fig. 2C). A similar observation about overconfidence of coalescent-based estimates has been made previously (Boskova *et al.* 2014, Stadler *et al.* 2015), which is likely due to the fact that the birth–death process explicitly models stochastic population changes while the coalescent-based approach assumes that the change in population size is deterministic. Finally, we show that the MCMC methods’ runtimes scale with the number of samples, while our analytical methods are essentially instantaneous regardless of the size of the tree (Fig. 2D).

**Figure 3.**
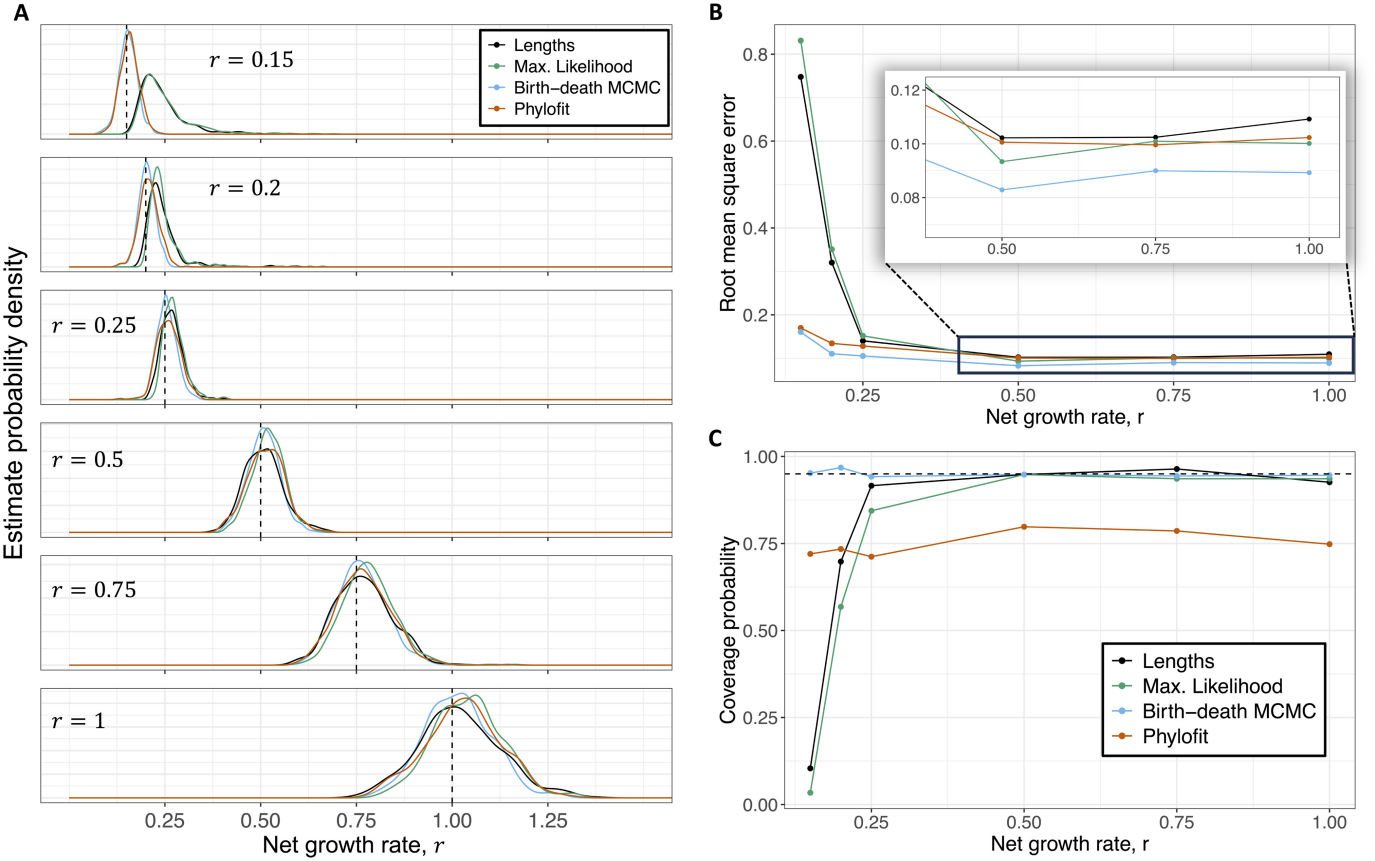
Performance across different growth rates, *r*. (A) Distribution of estimates from our methods, Phylofit, and the birth–death MCMC on 500 simulated ultrametric trees for each *r* value, where *r* is the growth rate. Simulated trees were generated assuming a continuous time birth–death branching process with *n *=* *100 and *T *=* *40. Birth rate *λ* was sampled from a uniform distribution on [r,1+r] and death rate μ=λ−r. Time units are arbitrary (e.g. years). (B) Root mean square error, normalized to account for the different true growth rates, for each method calculated for simulated data shown in (A) illustrates a decrease in accuracy for small growth rates. Performance of MCMC methods is less affected by small growth rates. (C) Accuracy of 95% confidence intervals of the methods using data from simulations in (A), measured as the fraction of simulations where the true growth rate falls within the estimated confidence intervals.

Similarly, we quantified the performance of our methods across *r* values. As shown in Fig. 3A and B, the four methods perform comparably well, with the birth–death MCMC having the lowest root mean square error. Again, Fig. 3C shows that our confidence intervals are accurate when r≥0.5, while coverage by Phylofit is below 80% when applied to the birth–death trees. In Fig. 3, the smallest growth rates show concerning performance, which motivated further investigation into the impact of small growth rates.

#### 3.1.2 Small growth rate diagnostic for method utilization

**Figure 4.**
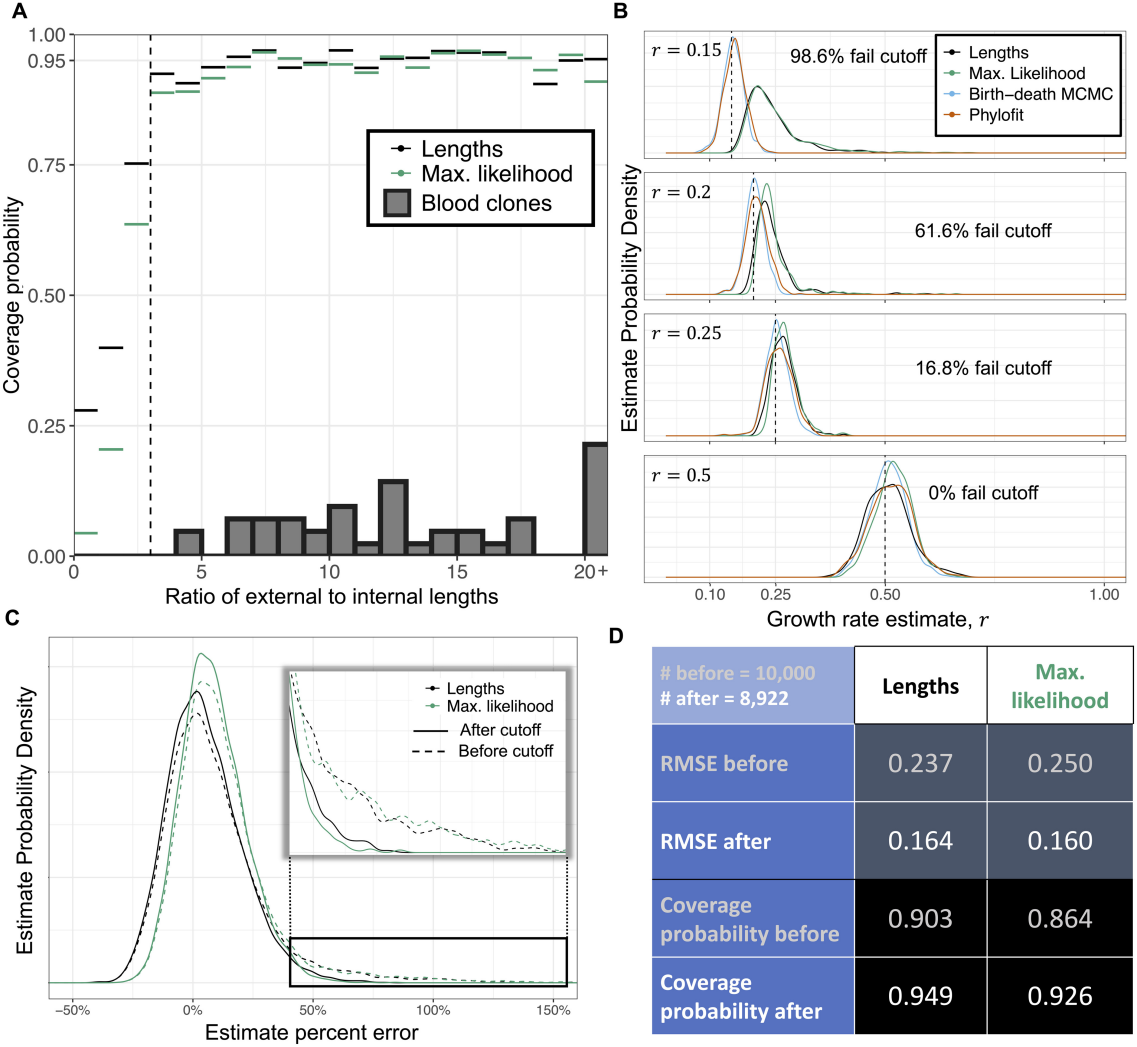
Small growth rate *r* diagnostic. (A) Trees with *n *=* *50 tips and *T *=* *40 were created from 10* *000 randomly sampled *r* values from a uniform distribution on (0.1, 1) and then binned by the ratio of external to internal lengths in increments of one. For corresponding estimates of *r*, accuracy of the 95% confidence intervals (CI) shows that the ratio of external to internal lengths can be used as a diagnostic to determine whether our method can accurately estimate growth rate. We use a ratio of 3 as a cutoff value as simulations with a ratio greater than 3 are captured by the 95% confidence intervals approximately 95% of the time. Minimum number of simulations captured in a single bin is 61. Histogram of external to internal lengths ratio for real blood clone data (Table 1) shown in grey for comparison, with frequency denoted by *y*-axis. (B) Distribution of estimates from our methods, Phylofit, and the birth–death MCMC on 500 simulated ultrametric trees from each *r* value, where *r* is the growth rate. Simulated trees generated assuming a continuous time birth–death branching process with *n *=* *100 and *T *=* *40. For each tree, we calculate the ratio of external lengths to internal lengths. For each growth rate, we show the percentage of trees which have a ratio of external to internal lengths below the diagnostic value of 3. (C) Relative fractional error distribution for four methods from the same simulations shown in (A) before (dashed lines, iterations = 10* *000) and after (solid lines, iterations = 8922) the cutoff of 3 was applied. Inset shows significant reduction in overestimates due to the diagnostic cutoff. (D) Normalized root mean square error (RMSE) and coverage of 95% confidence intervals for four methods using same simulations shown in (A) and (C) before and after the diagnostic cutoff was applied. The diagnostic provides a significant reduction in error and improvement in accuracy of 95% confidence intervals.

We investigated the performance failure at small growth rates and derived a diagnostic to determine when the growth rate is large enough for our analytical methods to be applicable (Fig. 4A). As shown in Fig. 3, our methods do not perform as well as the MCMC methods when *r* is small. To understand why, note that when *r* is large, the population grows rapidly, and the *n* sampled cells should all have distinct ancestors that were alive a short time after the initiation of the clone. Consequently, the genealogical tree will be nearly star-shaped, with long external branches and short internal branches near the root (see example in Fig. 1D). Our approximation was designed to work well under these circumstances. On the other hand, when *r *=* *0, so that the branching process is critical, the population size is nearly stable over time. Then the genealogy of the *n* sampled cells resembles Kingman’s coalescent, in which most coalescence events occur near the time of sampling, leading to long internal branches and short external branches. When *r* is small but positive, the genealogical tree will be star-shaped if the sampling time *T* is sufficiently large, but the internal branch lengths may still be long. Consequently, if *T* is not sufficiently large, then the constraint that the coalescence times must be less than *T* will affect the distribution of the coalescence times, and the approximation that we derived will not be accurate.

More specifically, we expect to obtain a star-shaped genealogical tree when the sample size *n* is much smaller than the expected population size *e^rT^*. Indeed, Theorem 1 (see Supplementary Section S1.2), establishes that the growth rate estimate should be accurate as long as $ne^{-rT}$ is small. When *n *=* *100 and *T *=* *40, as in the simulations presented in Fig. 4, the value of $ne^{-rT}$ equals 0.248, 0.034, and 0.005 when *r* equals 0.15, 0.20, and 0.25, respectively, which explains the large difference in the performance of our estimates over this range of values of *r*.

Note that if the birth and death rates are multiplied by a constant *c* and the sampling time is multiplied by 1/c, then the distribution of the shape of the genealogical tree of the sample will be unchanged except for a rescaling of the branch lengths. Consequently, the condition for the validity of our methods should be unchanged by this rescaling. Indeed, the product *rT* is unchanged by this rescaling, and our theorem shows that as long as $ne^{-rT}$ is small, our methods will perform well, regardless of the values of *λ* and *μ*. Because the aforementioned scaling relation allows us to fix one of the parameters for the purposes of simulation, we considered *T *=* *40 throughout. For fixed *T*, we show the effects of varying *r* and the μ/λ ratio in Supplementary Fig. S8, demonstrating the dependence of the tree shape on *r* rather than μ/λ.

When applying our methods to real data, we can compare the internal and external branch lengths of a given genealogical tree to diagnose when estimates should be reliable, because our methods work well when the tree is star-shaped. As shown in Fig. 4A, when the ratio of external to internal lengths is greater than or equal to 3, our methods and confidence intervals are accurate. Fig. 4B shows that most of the simulated trees with problematic small growth rates fail this diagnostic cutoff. Applying this cutoff to a simulated dataset with growth rates between 0.1 and 1 reduces overestimates and greatly improves performance (Fig. 4C and D). Notably, small growth rate clones at a relatively young clone age are unlikely to be observed in enough sampled cells in real data to make an accurate estimate; a requirement of n≥10 cells is unlikely to be satisfied in these clones. In fact, as shown in Fig. 4A, none of the 42 clones which we analyze from the blood datasets below has an external to internal length ratio less than 4, and only two clones have a ratio less than 5.

### 3.2 Application to human blood datasets

We applied our methods to single-cell-derived sequencing data from human blood (Table 1). The methods for generating the data are fairly similar across the studies: single hematopoietic progenitor cells were clonally expanded and each single-cell-derived colony was sequenced to a mean depth of roughly 15×, with slight differences depending on the study (Van Egeren *et al.* 2021, Fabre *et al.* 2022, Mitchell *et al.* 2022, Williams *et al.* 2022). Time-based ultrametric trees are used as input for our methods, Phylofit, and the birth–death MCMC. Manual annotation is required to identify clonal expansions, associate clones with specific drivers, and to remove nested subclonal expansions from the clone of interest. We generally designated the clones as annotated in the studies which produced the data (Van Egeren *et al.* 2021, Fabre *et al.* 2022, Mitchell *et al.* 2022, Williams *et al.* 2022) and provide details in Supplementary Section S4.4.

**Table 1.** Whole genome sequencing datasets of single-cell-derived colonies.^a^

Number of individuals	Number of clones	Data source	Diagnosis	References
11	18 (15 unique)	Adult peripheral blood (PB) and/or bone marrow (BM)	Myeloproliferative neoplasm (MPN)	Williams *et al.* (2022)
2	2	Adult BM	MPN	Van Egeren *et al.* (2021)
3	15	Adult PB and/or BM	Normal	Mitchell *et al.* (2022)
3	7	Adult PB	Normal	Fabre *et al.* (2022)
**19**	**42 (39 unique)**	**Total**		

a Number of clones indicates the number of clonal expansions with n≥10 cells sampled. As some clones profiled by Williams *et al.* (2022) had n≥10 cells sampled at multiple timepoints from the same clone, we also specify the number of unique clones. See Supplementary Section S4.4 for details on annotating clones.

First, we check our assumption of neutrality within expanding clones (i.e. all cells within the clone grow at approximately the same rate). Previous authors have studied the expected site frequency spectrum for a sample from a birth–death process. Letting $M_{n}^{k}$ denote the number of mutations inherited by *k* of the *n* sampled individuals, Durrett (2013) showed that as T→∞, for k≥2 we have

(8) $$E[M_{n}^{k}]∼\frac{n\nu }{r}\cdot \frac{1}{k(k-1)}.$$

**Figure 5.**
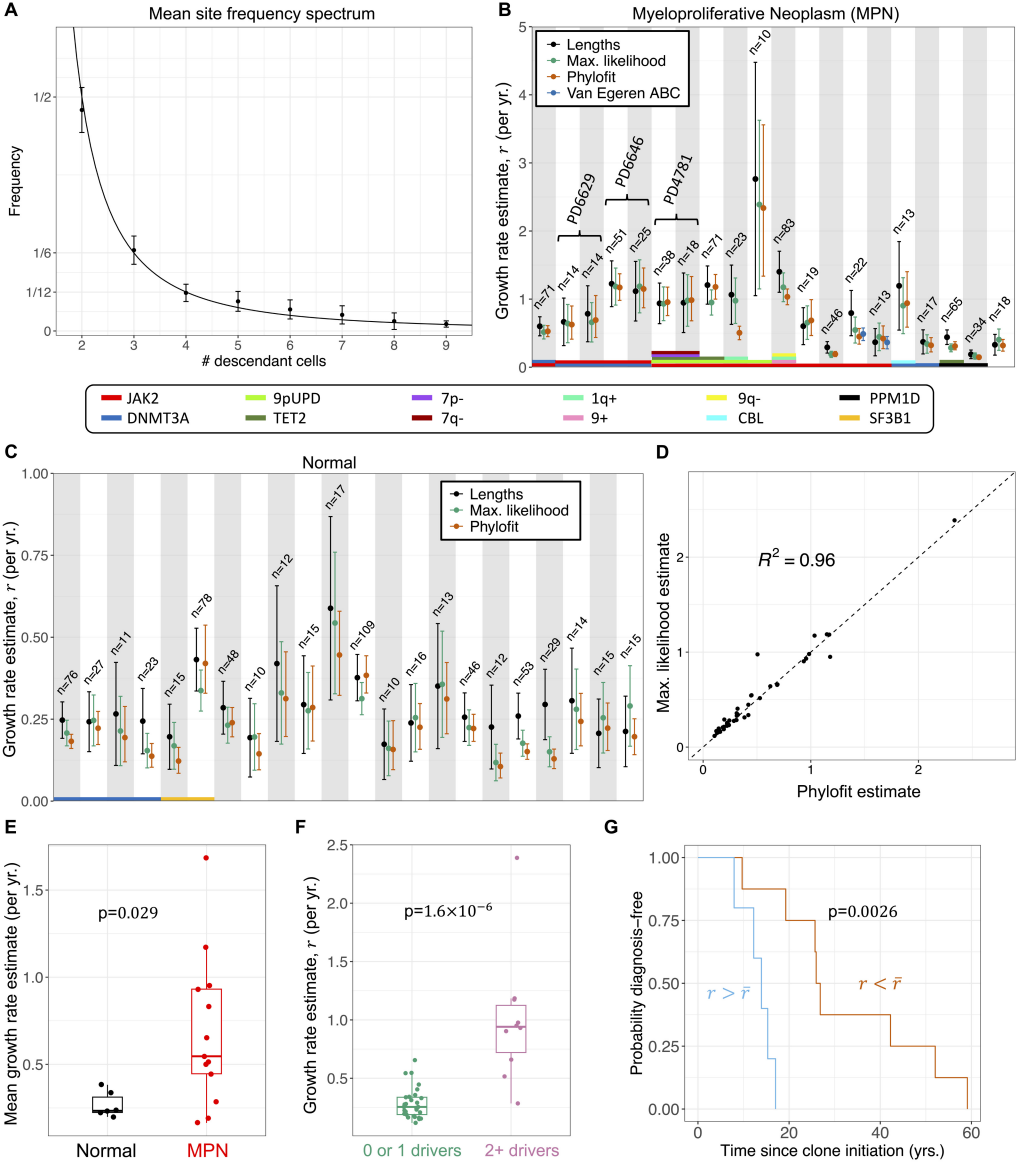
Applying estimates to blood data. (A) Averaged site frequency spectrum across 42 clones shows agreement with the $\frac{1}{k(k-1)}$ neutral expectation (solid line). Error bars show 95% confidence interval of the mean. (B–C) Our estimates and Phylofit for clones with n≥10 tips from individuals with (B) and without (C) myeloproliferative neoplasms (MPN) shows good agreement across methods. Brackets in (B) group estimates from the same clones in the same patient estimated from two distinct samples taken years apart, showing consistency of estimates. Note that we also include estimates from Van Egeren *et al.* (2021) in dark blue for the two clones from their dataset. (D) Correlation between our maximum likelihood estimate and estimates from Phylofit for all clones from (B) and (C). (E) Mean maximum likelihood net growth rate estimate for clones from patients with and without a diagnosis of MPN shows that more aggressive expansions are associated with MPN. (F) Maximum likelihood net growth rate estimate for clones with single or unknown drivers and multiple drivers show that fitness predicted by our methods is consistent with effects of known drivers. Non-parametric Mann–Whitney test used for *P*-value calculation in (E, F). (G) In the single most aggressive clone from each patient diagnosed with MPN, stratification by mean net growth rate $\bar{r}$ shows significant differences in Kaplan–Meier survival curves from clone initiation to MPN diagnosis (log-rank test *P* = 0.0026) though sample set was small (13 patients). At time of sampling, mean age of high growth rate group was 60.3 years, median was 50.4 years. Mean age of low growth rate group was 60.9 years, median was 63 years.

Gunnarsson *et al.* (2021) calculated the exact expectation in the case when the entire clone is sampled [see also (Bozic *et al.* 2016, Williams *et al.* 2016) for similar calculations]. Therefore, we expect the site frequency spectrum to follow the curve 1/k(k−1), where *k* equals the number of cells. In Fig. 5A, we show the averaged site frequency spectrum across all clones, with any nested subclones removed, along with the 95% confidence interval of the mean. The agreement between the observed mean and the expectation indicates neutrality within clones, consistent with previous conclusions in blood (Van Egeren *et al.* 2021, Fabre *et al.* 2022, Mitchell *et al.* 2022, Williams *et al.* 2022). For more detailed data on the site frequency spectrum for each clone, see Supplementary Table S5.

In applying the methods to real data, we found agreement across our two analytical methods and agreement with the estimates from Phylofit (see Fig. 5B–D). Close agreement was also found between our maximum likelihood method and the birth–death MCMC approach (*R*^2^ = 0.995, see Supplementary Fig. S2). Estimates using the internal lengths method were slightly higher than maximum likelihood in some clones, and we expect that this is due to non-random merging of lineages as a result of slight fitness differences within the clone (see Supplementary Fig. S3 for details). As discussed in Supplementary Section S4.2, we only include the estimates from Phylofit without including the sampled clonal fraction as a target, because clones have been shown to behave unpredictably at high clonal fractions, decelerating more than would be expected by a logistic growth trajectory (Fabre *et al.* 2022, Mitchell *et al.* 2022). Also, sampled clonal fraction and/or variant allele frequency (VAF) may be a poor estimate of mutant allele burden in progenitors and HSCs (see Supplementary Section S4.2), possibly due to lineage bias in mutated cells, such as the erythroid lineage bias observed in *JAK2* mutants (Van Egeren *et al.* 2021).

The most fit clones (those with fastest growth rates) were observed in patients with myeloproliferative neoplasms (MPN). As shown in Fig. 5E, we found significantly increased estimates of mean detected clone fitness in individuals diagnosed with MPN as opposed to healthy adults (*P* = 0.029). Additionally, Fig. 5F shows that multiple-driver clones have significantly increased rates of expansion as compared to clones with just one or zero known driver mutations (*P* = $1.6\times 10^{-6}$). This suggests increasing fitness effects from the accumulation of additional mutations. Higher growth rates may also be associated with shorter time from clone initiation to cancer diagnosis (log-rank *P* = 0.0026), as shown in Kaplan–Meier curves in Fig. 5G. Here the clone initiation time is estimated to occur 1/r years before the first coalescence (i.e. first surviving symmetric division). Together, these findings indicate that mechanistic rates for clonal dynamics such as the early growth rate may provide clinically important information in our understanding of hematopoietic stem and progenitor cell evolution and transformation to malignancy.

#### 3.2.1 Longitudinal validation of clone growth estimates

We leveraged available longitudinal data to validate our growth rate estimates. Again, for single-cell-derived data, we used a lower bound of *n *=* *10 cells per clone to include in our analysis. For longitudinal bulk data, we restrict analysis to expanding clones with a minimum of four timepoints available of the same bulk cell type. Further, because coalescent estimates are relevant for the early growth rate, we require that longitudinal data have at least two samples with a variant allele frequency between 0 and 0.25. The longitudinal data consists of peripheral (whole) blood samples (Fabre *et al.* 2022) and peripheral blood granulocyte samples (Williams *et al.* 2022). It has been suggested that clonal fraction may differ across different blood cell types (granulocyte versus whole versus mononuclear) (Van Egeren *et al.* 2021). Data from Williams *et al.* (2022) is consistent with this finding, as there is significantly different clonal fraction across sampled cell type in three out of four patients where multiple cell types were sampled within a month of each other (see Supplementary. Fig. S6). Therefore, we require that a consistent type be used within each longitudinal growth rate estimate. For more details on the criteria for analysis, see Supplementary Section S4.3.1.

**Figure 6.**
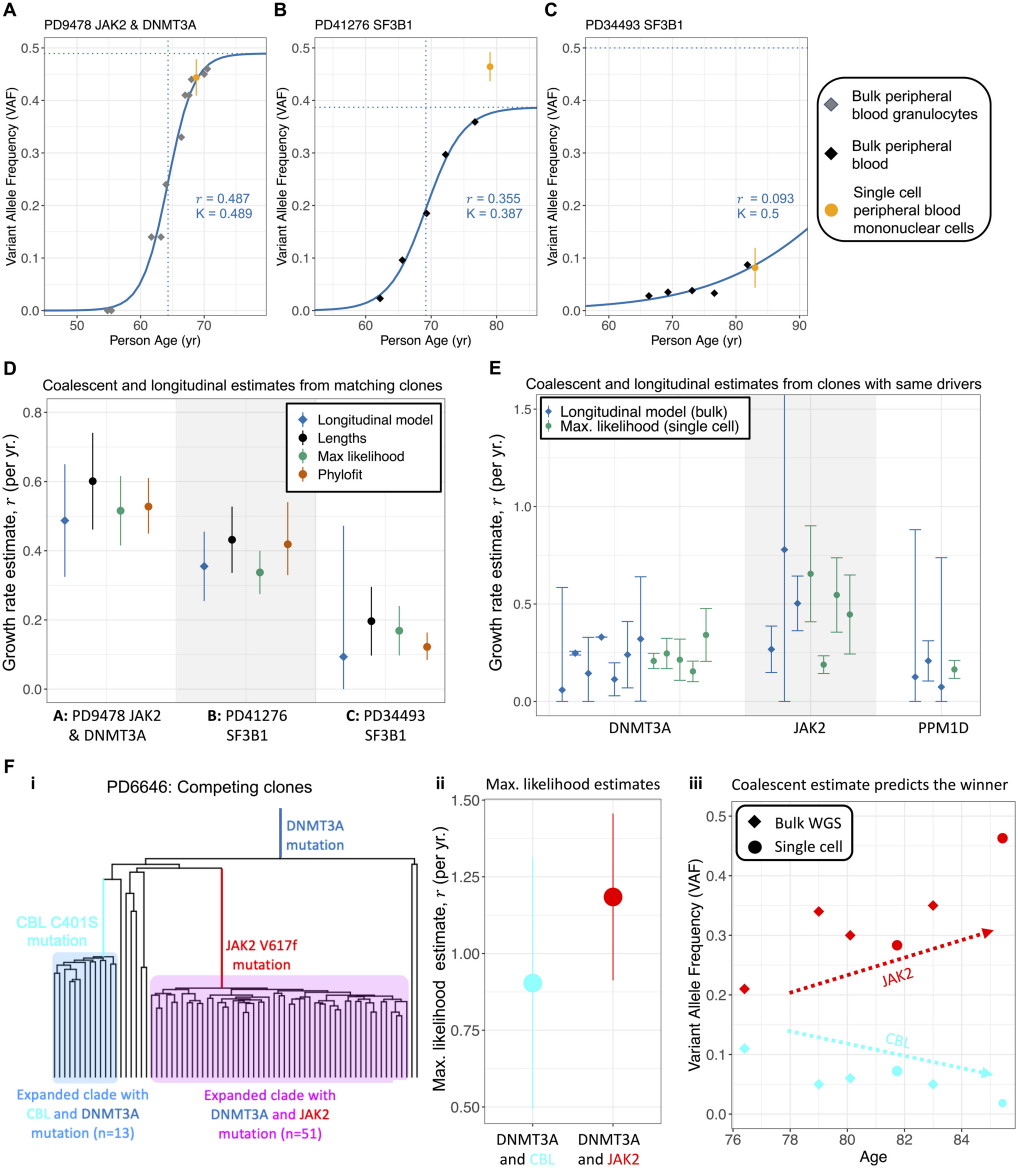
Longitudinal validation. (A–C) Logistic fit to longitudinal data for three clones which have both single-cell and longitudinal data. Only longitudinal bulk WGS data were used for fitting. Single-cell colony clonal fraction (divided by 2) and 95% confidence intervals are shown in orange. Source for (A) is Williams *et al.* (2022), and source for (B) and (C) is Fabre *et al.* (2022). (D) Longitudinal and single-cell estimates for each of the clones in (A–C) show agreement across data types. (E) Longitudinal and single-cell estimates for different clones sharing the same driver. (F) Clonal competition between a *DNMT3A* + *CBL* clone and a *DNMT3A* + *JAK2* clone shown in the reconstructed phylogeny (i). Maximum likelihood single-cell estimate from each clone (ii) shows that the *DNMT3A* + *JAK2* clone likely has higher fitness. Longitudinal data (iii) shows that the *DNMT3A* + *JAK2* clone increases in VAF over time while the *DNMT3A* + *CBL* clone decreases, confirming that the *DNMT3A* + *JAK2* clone has higher fitness, as predicted by our maximum likelihood estimate. All error bars represent 95% confidence intervals.

We analyzed four clones from Williams *et al.* (2022) and 56 clones from Fabre *et al.* (2022) that had appropriate longitudinal data. Of these 60 clones, 3 have sufficient matched data from single-cell-derived whole genome sequencing (WGS) samples [1 clone from Williams *et al.* (2022) and 2 clones from Fabre *et al.* (2022)] to allow for orthogonal estimates from the same clone. Results for these three clones are shown in Fig. 6A–C, along with a logistic growth model fit. While we show the corresponding single-cell colony clonal fraction (orange, divided by 2 to scale to the VAF of a diploid mutant), we do not use this data point in the fitting as the different cell type may affect the clonal fraction, as noted above. The logistic growth model is used primarily to identify the growth rate, *r*, and is chosen because it has been shown to face fewer parameter identifiability issues than other sigmoid growth models, such as Gompertz or Richards’, when applied to similar data (Simpson *et al.* 2022). For details on the longitudinal modeling, see Supplementary Section S4.3.2.

In comparing the growth rates from the longitudinal fits to our methods and Phylofit (Fig. 6D), we found general agreement in the estimates, though we note the wide confidence intervals especially from the logistic model fit. Additional longitudinal data from Fabre *et al.* (2022), although not from clones with matched single-cell data, was also used to compare to our coalescent estimates. First, we identify longitudinal clones with drivers also present in the single-cell data. Then, we fit the logistic growth model to these longitudinal clones. After filtering (see Supplementary Section S4.3.1) and excluding the three clones shown in Fig. 6A–C, there were 13 clones with longitudinal data and a driver gene also found in single-cell clones. This data comes from clones with a mutation in one of the following genes: *DNMT3A*, *JAK2*, or *PPM1D*. The estimated growth rates are shown in Fig. 6E. Similar growth rates in the same driver genes shows general consistency across all methods, though the small amount of data and wide confidence intervals limit the conclusions that can be drawn. Interestingly, similar longitudinal modeling of white blood cell (WBC) counts in chronic lymphocytic leukemia (CLL) (Gruber *et al.* 2019) produced comparable estimates of CLL growth rates [median 0.50 per year, interquartile range (IQR) = 0.30−0.86] to our estimates based on single-cell data for MPN clone growth rates (median 0.64 per year, IQR = 0.40−0.96). See Supplementary Fig. S9 for more details.

Finally, we considered competing clones within the same patient. If our estimates are relevant for clonal fitness, we would expect that clones with higher estimated growth rates should out-compete clones with lower estimated growth rates. The only example of competing clones with sufficient single-cell data comes from patient PD6646 from Williams *et al.* (2022) (Fig. 6F). A *CBL* and a *JAK2* mutation gave rise to two independent clones, both of which already have a *DNMT3A* mutation (Fig. 6F, i). By our maximum likelihood estimate, the *DNMT3A* + *JAK2* clone is slightly more fit than the *DNMT3A* + *CBL* clone (Fig. 6F, ii). Both Phylofit and our internal lengths method also estimate a higher growth rate for the *JAK2* clone. While this patient was undergoing treatment in this time period and the trajectory does not appear logistic, the *JAK2* clone increases in variant allele frequency while the *CBL* clone decreases (Fig. 6F, iii), consistent with our estimate suggesting that the *JAK2* clone is more fit. There is an important caveat in this example because the specific interactions between clone/mutation and treatment may be responsible for the increasing/decreasing VAF, which is not accounted for by our estimate of fitness that characterizes early growth before treatment would have begun.

## 4 Discussion

We developed new methods using coalescent theory to estimate rates of clonal expansion (and clone age) at greatly reduced computational expense. Leveraging previous work (Lambert 2018), we validated our methods using efficient computational realizations of phylogenies resulting from birth–death branching processes. We then applied our methods to single-cell resolution data from blood, showing that our growth rate estimates are both meaningful and consistent in biological and clinical contexts. We found general consistency of estimates with a previously published MCMC-based approach, Phylofit (Williams *et al.* 2022) ($R^{2}=0.94$–0.96), and a birth–death MCMC approach (Stadler 2009) ($R^{2}=0.965$–0.995). Where possible, we validated our estimates using single-cell data from multiple timepoints, and also show that our estimates are consistent with and generally more precise than orthogonal estimates of net growth rate derived from longitudinal bulk data. Because they are based on analytical results, our methods for estimating growth rates from phylogenetic reconstruction are simple and run quickly without sacrificing accuracy. For future datasets with a higher number of sampled cells *n* and larger numbers of patients and clones, near instantaneous runtime at any tree size may be a critical feature separating our methods from MCMC- or ABC-based alternatives. We provide a simple and easy-to-use R package, *cloneRate*, which will allow other researchers to estimate growth rates with their own input data.

For testing model performance on simulated data, we use results of Harris *et al.* (2020) and Lambert (2018) to reconstruct the exact genealogy of a sample of size *n* from a birth–death process at time *T*, conditional on the population size being at least *n* at time *T*. This method avoids the need to simulate the entire large clonal population starting from a single cell as is commonly performed in other methods. From a mathematical point of view, the idea of using the coalescent point process to obtain results about statistics such as the site frequency spectrum and the allele frequency spectrum goes back to Lambert (2009) and was later developed further (Champagnat and Lambert 2012, Champagnat and Henry 2016, Delaporte *et al.* 2016), and then was recently applied to cancer modeling by Dinh *et al.* (2020). Here we combine these ideas with the results from Lambert (2018) to obtain asymptotic results for quantities that can be derived from a large sample from a birth–death process. By taking advantage of the independence that is inherent in the coalescent point process, we are able to apply the *m*-dependent Central Limit Theorem to show that the total internal branch length, which can be used to estimate the growth rate of the process, has an asymptotic normal distribution. This observation allows us to obtain an asymptotically valid confidence interval for the growth rate; to our knowledge, ours is the first method for confidence interval construction that does not rely on Bayesian inference techniques. Finally, this is a unifying method for growth rate estimation that is applicable to many biologically relevant models assumed in previous works for clonal dynamics in blood (Watson *et al.* 2020, Van Egeren *et al.* 2021, Fabre *et al.* 2022, Mitchell *et al.* 2022, Williams *et al.* 2022).

Acknowledging that this is both a limitation and a strength, our methods estimate only the growth rate in the early expansion phase, when growth is approximately exponential. Growth rates following the initial expansion phase may change over time in unpredictable ways and this, in most cases, should not affect our results. As such, our methods work well for clones with star-shaped trees that are common in blood and other somatic cell datasets. In the case of critical branching behavior (i.e. trees with relatively longer internal branches), our simulations indicate that the MCMC approaches should be applied. In focusing only on the early growth rate, our methods do not rely on assumptions of the overall growth trajectory. Additionally, we have shown that early growth rates are relevant to the greater context of clonal and malignant hematopoiesis. For example, we found that higher growth rates are associated with shorter time from clone initiation to MPN diagnosis. The association between MPN diagnosis and growth rate suggests a possible avenue for early detection by predicting which patients are more likely to remain asymptomatic and which are more likely to undergo malignant transformation. Understanding the role of evolutionary dynamics to predict risk of progression in clonal hematopoiesis and provide prognostic information in hematological malignancies has been noted as a top clinical priority (Savona *et al.* 2015, Stahl *et al.* 2023).

Further, multi-driver clones show significantly increased rates of expansion, suggesting possible cumulative and/or synergistic effects of driver mutations. We found wide heterogeneity of fitness effects for *JAK2* clones, consistent with previous findings (Fabre *et al.* 2022), and relatively low fitness effects with smaller variation for *DNMT3A* clones. In the context of clonal hematopoiesis, single hit drivers with lower growth rates may increase risk for MPN by increasing the reservoir of cells at risk of additional stochastic mutations, thus initiating multi-hit driver clones with potentially additive fitness effects. There are other possible benefits to knowing the early rate of expansion. For example, early expansion rates are affected by fewer outside pressures such as treatment (Bolton *et al.* 2020) and may be more consistent across patients.

Our findings also provide guidance to experimental researchers designing single-cell DNA sequencing experiments that aim to determine clone fitness. The minimum number of sampled cells required for reliable estimates of growth rates falls roughly between 10 and 30, depending on desired accuracy (see Fig. 2). Bulk whole genome sequencing performed prior to single-cell experiments could provide variant allele frequency information that can be used to estimate the cell fraction of clones of interest. Then, the total number of cells sequenced can be decided in a way that ensures enough sampled cells from clones of interest are included, while reducing overall costs.

One limitation is that current methods rely on the manual annotation of clones from a phylogenetic tree. While this is currently a fairly easy task given the relatively small size of single-cell DNA sequencing datasets, it may become more challenging for expected increases in throughput (Evrony *et al.* 2021). An automated way to detect clonal expansions and distinguish normal cell turnover from expansions may be required to effectively scale the application of our methods. Such an automated algorithm would likely have to leverage not just the distribution of coalescence times, but also measures of tree balance.

## 5 Conclusion

Phylodynamics for human somatic data and cancer is a rapidly growing area and new tools are needed for useful applications (Stadler *et al.* 2021, Househam *et al.* 2022, Lewinsohn *et al.* 2023). With our methods, phylogenetic reconstruction can become an even more powerful tool to infer the past evolutionary dynamics of a population of cells. It has been hypothesized that individuals at high risk of developing myeloid malignancies can be identified before presenting with any symptoms (Abelson *et al.* 2018). Knowing which drivers are associated with more aggressive expansions will provide clinicians with better tools to direct treatment and/or prevention strategies. Additionally, clonal expansions without known drivers can provide mechanistic and biological insight. While blood is currently the most convenient medium for creation of single-cell-derived DNA sequencing data and validation of these methods, age-related clonal expansions are also a feature of somatic evolution in tissues with spatial organization. Selection of the same drivers are found at similar burdens in solid tissues across patients, and thus accurate phylogenetic reconstruction in solid tissues may allow our method to be applicable in a variety of disease types. More comprehensive methods and datasets leading to the construction of more accurate phylogenetic trees (Kang *et al.* 2022, Kozlov *et al.* 2022), when combined with the methods presented here, will enable researchers and clinicians to quickly draw conclusions about net growth rate from mutational data.

## Supplementary Material

btad561_Supplementary_Data

## Data Availability

The de-identified ultrametric trees derived from mutational data analyzed herein are included in the package *cloneRate* for convenience; please cite the appropriate references if this data is used (Van Egeren *et al.* 2021, Fabre *et al.* 2022, Mitchell *et al.* 2022, Williams *et al.* 2022). All modeling results for simulated and real data are provided as Supplementary tables.

## References

[btad561-B1] Abelson S , Collord G, Ng SW et al Prediction of acute myeloid leukaemia risk in healthy individuals. Nature 2018;559:400–4. 29988082 10.1038/s41586-018-0317-6 PMC6485381

[btad561-B2] Antle C , Klimko L, Harkness W. Confidence intervals for the parameters of the logistic distribution. Biometrika 1970;57:397–402.

[btad561-B3] Bolton KL , Ptashkin RN, Gao T et al Cancer therapy shapes the fitness landscape of clonal hematopoiesis. Nat Genet 2020;52:1219–26. 33106634 10.1038/s41588-020-00710-0 PMC7891089

[btad561-B4] Boskova V , Bonhoeffer S, Stadler T. Inference of epidemiological dynamics based on simulated phylogenies using birth-death and coalescent models. PLoS Comput Biol 2014;10:e1003913. 25375100 10.1371/journal.pcbi.1003913 PMC4222655

[btad561-B5] Bouckaert R , Vaughan TG, Barido-Sottani J et al Beast 2.5: an advanced software platform for Bayesian evolutionary analysis. PLoS Comput Biol 2019;15:e1006650. 30958812 10.1371/journal.pcbi.1006650 PMC6472827

[btad561-B6] Bozic I , Gerold JM, Nowak MA. Quantifying clonal and subclonal passenger mutations in cancer evolution. PLoS Comput Biol 2016;12:e1004731. 26828429 10.1371/journal.pcbi.1004731 PMC4734774

[btad561-B7] Champagnat N , Henry B. Moments of the frequency spectrum of a splitting tree with neutral Poissonian mutations. Electron J Probab 2016;21:1–34.

[btad561-B8] Champagnat N , Lambert A. Splitting trees with neutral Poissonian mutations I: small families. Stoch Process Their Appl 2012;122:1003–33.

[btad561-B9] Curtius K , Wright NA, Graham TA. An evolutionary perspective on field cancerization. Nat Rev Cancer 2018;18:19–32. 29217838 10.1038/nrc.2017.102

[btad561-B10] Delaporte C , Achaz G, Lambert A. Mutational pattern of a sample from a critical branching population. J Math Biol 2016;73:627–64. 26748918 10.1007/s00285-015-0964-2

[btad561-B11] Dinh KN , Jaksik R, Kimmel M et al Statistical inference for the evolutionary history of cancer genomes. Statist Sci 2020;35:129–44. 10.1214/19-sts7561 PMC12519734 41098675

[btad561-B12] Durrett R. Population genetics of neutral mutations in exponentially growing cancer cell populations. Ann Appl Probab 2013;23:230. 23471293 10.1214/11-aap824 PMC3588108

[btad561-B13] Evrony GD , Hinch AG, Luo C. Applications of single-cell DNA sequencing. Annu Rev Genomics Hum Genet 2021;22:171–97. 33722077 10.1146/annurev-genom-111320-090436 PMC8410678

[btad561-B14] Fabre MA , de Almeida JG, Fiorillo E et al The longitudinal dynamics and natural history of clonal haematopoiesis. Nature 2022;606:335–42. 35650444 10.1038/s41586-022-04785-z PMC9177423

[btad561-B15] Geiger H , De Haan G, Florian M. The ageing haematopoietic stem cell compartment. Nat Rev Immunol 2013;13:376–89. 23584423 10.1038/nri3433

[btad561-B16] Gerstung M , Jolly C, Leshchiner I et al; PCAWG Consortium. The evolutionary history of 2,658 cancers. Nature 2020;578:122–8. 32025013 10.1038/s41586-019-1907-7 PMC7054212

[btad561-B17] Gillis NK , Ball M, Zhang Q et al Clonal haemopoiesis and therapy-related myeloid malignancies in elderly patients: a proof-of-concept, case-control study. Lancet Oncol 2017;18:112–21. 27927582 10.1016/S1470-2045(16)30627-1 PMC7771361

[btad561-B18] Griffiths RC , Tavaré S. Sampling theory for neutral alleles in a varying environment. Philos Trans R Soc Lond B Biol Sci 1994;344:403–10. 7800710 10.1098/rstb.1994.0079

[btad561-B19] Griffiths RC , Tavaré S. The age of a mutation in a general coalescent tree. Stoch Model 1998;14:273–95.

[btad561-B20] Gruber M , Bozic I, Leshchiner I et al Growth dynamics in naturally progressing chronic lymphocytic leukaemia. Nature 2019;570:474–9. 31142838 10.1038/s41586-019-1252-x PMC6630176

[btad561-B21] Gunnarsson EB , Leder K, Foo J. Exact site frequency spectra of neutrally evolving tumors: a transition between power laws reveals a signature of cell viability. Theor Popul Biol 2021;142:67–90. 34560155 10.1016/j.tpb.2021.09.004

[btad561-B22] Harris SC , Johnston SG, Roberts MI. The coalescent structure of continuous-time Galton–Watson trees. Ann Appl Probab 2020;30:1368–414.

[btad561-B23] Househam J , Heide T, Cresswell GD et al Phenotypic plasticity and genetic control in colorectal cancer evolution. Nature 2022;611:744–753. 36289336 10.1038/s41586-022-05311-x PMC9684078

[btad561-B24] Ignatieva A , Hein J, Jenkins PA. A characterisation of the reconstructed birth–death process through time rescaling. Theor Popul Biol 2020;134:61–76. 32439294 10.1016/j.tpb.2020.05.001

[btad561-B25] Jaiswal S , Fontanillas P, Flannick J et al Age-related clonal hematopoiesis associated with adverse outcomes. N Engl J Med 2014;371:2488–98. 25426837 10.1056/NEJMoa1408617 PMC4306669

[btad561-B26] Jonason AS , Kunala S, Price GJ et al Frequent clones of p53-mutated keratinocytes in normal human skin. Proc Natl Acad Sci U S A 1996;93:14025–9. 8943054 10.1073/pnas.93.24.14025 PMC19488

[btad561-B27] Kang S , Borgsmüller N, Valecha M et al SIEVE: joint inference of single-nucleotide variants and cell phylogeny from single-cell DNA sequencing data. Genome Biol 2022;23:248. 36451239 10.1186/s13059-022-02813-9 PMC9714196

[btad561-B28] Karcher MD , Palacios JA, Lan S et al phylodyn: an R package for phylodynamic simulation and inference. Mol Ecol Resour 2017;17:96–100. 27801980 10.1111/1755-0998.12630 PMC5466693

[btad561-B29] Kingman JFC. The coalescent. Stoch Process Their Appl 1982;13:235–48.

[btad561-B30] Kozlov A , Alves JM, Stamatakis A et al CellPhy: accurate and fast probabilistic inference of single-cell phylogenies from scDNA-seq data. Genome Biol 2022;23:37. 35081992 10.1186/s13059-021-02583-w PMC8790911

[btad561-B31] Lambert A. The allelic partition for coalescent point processes. Markov Process Relat Fields 2009;15:359–86.

[btad561-B32] Lambert A. The coalescent of a sample from a binary branching process. Theor Popul Biol 2018;122:30–5. 29704514 10.1016/j.tpb.2018.04.005

[btad561-B33] Lee-Six H , Øbro NF, Shepherd MS et al Population dynamics of normal human blood inferred from somatic mutations. Nature 2018;561:473–8. 30185910 10.1038/s41586-018-0497-0 PMC6163040

[btad561-B34] Lee-Six H , Olafsson S, Ellis P et al The landscape of somatic mutation in normal colorectal epithelial cells. Nature 2019;574:532–7. 31645730 10.1038/s41586-019-1672-7

[btad561-B35] Lewinsohn MA , Bedford T, Müller NF et al State-dependent evolutionary models reveal modes of solid tumour growth. Nat Ecol Evol 2023;7:581–96. 36894662 10.1038/s41559-023-02000-4 PMC10089931

[btad561-B36] Martincorena I , Fowler JC, Wabik A et al Somatic mutant clones colonize the human esophagus with age. Science 2018;362:911–7. 30337457 10.1126/science.aau3879 PMC6298579

[btad561-B37] Martincorena I , Roshan A, Gerstung M et al High burden and pervasive positive selection of somatic mutations in normal human skin. Science 2015;348:880–6. 25999502 10.1126/science.aaa6806 PMC4471149

[btad561-B38] Martinez P , Timmer MR, Lau CT et al Dynamic clonal equilibrium and predetermined cancer risk in Barrett’s oesophagus. Nat Commun 2016;7:12158. 27538785 10.1038/ncomms12158 PMC4992167

[btad561-B39] Mitchell E , Spencer Chapman M, Williams N et al Clonal dynamics of haematopoiesis across the human lifespan. Nature 2022;606:343–50. 35650442 10.1038/s41586-022-04786-y PMC9177428

[btad561-B40] Moeller ME , Pere NVM, Werner B et al Measures of genetic diversification in somatic tissues at bulk and single cell resolution suggest sources of unknown stochasticity. eLife 2023;12:RP89780. 10.7554/eLife.89780 PMC10945735 38265286

[btad561-B41] Nelder JA , Mead R. A simplex method for function minimization. Comp J 1965;7:308–13.

[btad561-B42] Polanski A , Kimmel M. New explicit expressions for relative frequencies of single-nucleotide polymorphisms with application to statistical inference on population growth. Genetics 2003;165:427–36. 14504247 10.1093/genetics/165.1.427 PMC1462751

[btad561-B43] Sano S , Horitani K, Ogawa H et al Hematopoietic loss of Y chromosome leads to cardiac fibrosis and heart failure mortality. Science 2022;377:292–7. 35857592 10.1126/science.abn3100 PMC9437978

[btad561-B44] Savona MR , Malcovati L, Komrokji R et al; MDS/MPN International Working Group. An international consortium proposal of uniform response criteria for myelodysplastic/myeloproliferative neoplasms (MDS/MPN) in adults. Blood 2015;125:1857–65. 25624319 10.1182/blood-2014-10-607341 PMC4915792

[btad561-B45] Schenz J , Rump K, Siegler BH et al Increased prevalence of clonal hematopoiesis of indeterminate potential in hospitalized patients with COVID-19. Front Immunol 2022;13:968778. 36311800 10.3389/fimmu.2022.968778 PMC9614713

[btad561-B46] Scott J , Marusyk A. Somatic clonal evolution: a selection-centric perspective. Biochim Biophys Acta Rev Cancer 2017;1867:139–50. 28161395 10.1016/j.bbcan.2017.01.006

[btad561-B47] Simpson MJ , Browning AP, Warne DJ et al Parameter identifiability and model selection for sigmoid population growth models. J Theor Biol 2022;535:110998. 34973274 10.1016/j.jtbi.2021.110998

[btad561-B48] Slatkin M , Hudson RR. Pairwise comparisons of mitochondrial DNA sequences in stable and exponentially growing populations. Genetics 1991;129:555–62. 1743491 10.1093/genetics/129.2.555 PMC1204643

[btad561-B49] Stadler T. On incomplete sampling under birth–death models and connections to the sampling-based coalescent. J Theor Biol 2009;261:58–66. 19631666 10.1016/j.jtbi.2009.07.018

[btad561-B50] Stadler T , Pybus OG, Stumpf MP. Phylodynamics for cell biologists. Science 2021;371:eaah6266 33446527 10.1126/science.aah6266

[btad561-B51] Stadler T , Vaughan TG, Gavryushkin A et al How well can the exponential-growth coalescent approximate constant-rate birth–death population dynamics? Proc Biol Sci 2015;282:20150420. 25876846 10.1098/rspb.2015.0420 PMC4426635

[btad561-B52] Stahl M , Abdel-Wahab O, Wei AH et al An agenda to advance research in myelodysplastic syndromes: a top 10 priority list from the first international workshop in mds. Blood Adv 2023;7:2709–14. 36260702 10.1182/bloodadvances.2022008747 PMC10333740

[btad561-B53] Steensma DP , Ebert BL. Clonal hematopoiesis as a model for premalignant changes during aging. Exp Hematol 2020;83:48–56. 31838005 10.1016/j.exphem.2019.12.001 PMC7103509

[btad561-B54] Suda K , Nakaoka H, Yoshihara K et al Clonal expansion and diversification of cancer-associated mutations in endometriosis and normal endometrium. Cell Rep 2018;24:1777–89. 30110635 10.1016/j.celrep.2018.07.037

[btad561-B55] Tall AR , Fuster JJ. Clonal hematopoiesis in cardiovascular disease and therapeutic implications. Nat Cardiovasc Res 2022;1:116–24. 36337911 10.1038/s44161-021-00015-3 PMC9631799

[btad561-B56] Van Egeren D , Escabi J, Nguyen M et al Reconstructing the lineage histories and differentiation trajectories of individual cancer cells in myeloproliferative neoplasms. Cell Stem Cell 2021;28:514–23.e9. 33621486 10.1016/j.stem.2021.02.001 PMC7939520

[btad561-B57] van Zeventer IA , de Graaf AO, Wouters HJ et al Mutational spectrum and dynamics of clonal hematopoiesis in anemia of older individuals. Blood 2020;135:1161–70. 32243522 10.1182/blood.2019004362

[btad561-B58] Vaughan TG , Drummond AJ. A stochastic simulator of birth–death master equations with application to phylodynamics. Mol Biol Evol 2013;30:1480–93. 23505043 10.1093/molbev/mst057 PMC3649681

[btad561-B59] Warren JT , Link DC. Clonal hematopoiesis and risk for hematologic malignancy. Blood 2020;136:1599–605. 32736382 10.1182/blood.2019000991 PMC8209630

[btad561-B60] Watson CJ , Papula A, Poon GY et al The evolutionary dynamics and fitness landscape of clonal hematopoiesis. Science 2020;367:1449–54. 32217721 10.1126/science.aay9333

[btad561-B61] Williams MJ , Werner B, Barnes CP et al Identification of neutral tumor evolution across cancer types. Nat Genet 2016;48:238–44. 26780609 10.1038/ng.3489 PMC4934603

[btad561-B62] Williams N , Lee J, Mitchell E et al Life histories of myeloproliferative neoplasms inferred from phylogenies. Nature 2022;602:162–8. 35058638 10.1038/s41586-021-04312-6

[btad561-B63] Yokoyama A , Kakiuchi N, Yoshizato T et al Age-related remodelling of oesophageal epithelia by mutated cancer drivers. Nature 2019;565:312–7. 30602793 10.1038/s41586-018-0811-x

