# Supplementary material for: cloneRate: fast estimation of single-cell clonal dynamics using coalescent theory
Source: Bioinformatics. 2023 Sep 12;39(9):btad561. doi: 10.1093/bioinformatics/btad561 (PMC10534056; doi:10.1093/bioinformatics/btad561)
Supplement: btad561_Supplementary_Data [file btad561_supplementary_data.zip › cloneRate_Supplementary.pdf]

cloneRate: fast estimation of single-cell clonal dynamics using coalescent theory:
Supplementary Material
Authors: Johnson B, Shuai Y, Schweinsberg J, and Curtius K. 2023

1 Supplementary Methods

1.1 Simulating the exact genealogy

We present here Lambert's construction of the exact genealogy of a sample of size n at time T from a birth-death process[1]. The idea is to describe the genealogical tree of n individuals from $n-1$ random variables $H_1, \dots, H_{n-1}$ which represent coalescence times. To reconstruct the tree from the coalescence times, we begin by drawing a vertical line of height T. We then draw vertical lines of heights $H_1, \dots, H_{n-1}$ and, at the top of each vertical line, draw a horizontal line to the left, stopping when it hits a vertical branch. The resulting tree is ultrametric, meaning that the root to tip distance is the same for all tips. See Main text figure 1C-D. This construction is known as a coalescent point process and goes back to the work of Popovic[2] and of Aldous and Popovic[3] in the setting of critical branching processes.

By building on earlier work of Stadler[4] and Lambert and Stadler[5], who considered the case in which each individual in the population is sampled with some fixed probability y, Lambert[1] showed that we obtain exact the genealogy of a sample of size n from a birth-death process at time T, conditional on the population size at time T being at least n, if we choose $H_1, \dots, H_{n-1}$ by the following two-step procedure:

1. Choose a random variable Y with probability density function on $(0, 1)$ given by

$$f_Y(y) = \frac{n\delta_T y^{n-1}}{(y + \delta_T - y\delta_T)^{n+1}}, \qquad \delta_T = \frac{re^{-rT}}{\lambda(1 - e^{-rT}) + re^{-rT}}. \tag{9}$$

2. Conditional on $Y = y$, let the random variables $H_1, \dots, H_{n-1}$ be i.i.d. with probability density function on $(0, T)$ given by

$$f_{H_i|Y=y}(t) = \frac{y\lambda + (r - y\lambda)e^{-rT}}{y\lambda(1 - e^{-rT})} \cdot \frac{y\lambda r^2 e^{-rt}}{(y\lambda + (r - y\lambda)e^{-rt})^2}. \tag{10}$$

Note that the formula for the density of Y comes from equation (12) in Lambert[1], and that δ_T here is $1 - a$ in Lambert[1]. The density for H_i comes from equation (7) in Lambert[1]. One can check that the resulting joint density for $H_1, \dots, H_{n-1}$ matches the joint density for the ordered coalescence times given in Proposition 19 of Harris et al.[6].

While Lambert's construction is only exact when the birth and death rates are constant over time, leading to a population which grows exponentially at a constant rate, Cheek[7] has shown that under certain conditions, the construction remains approximately valid even when the growth rate of the

population slows over time, provided that the population is still growing superlinearly at the time T when the sample is taken. For example, this method should give a good approximation in certain models of logistic population growth, provided that the sample is taken before the population reaches a fraction x of its carrying capacity, where $0 < x < 1$[7]. Consequently, we believe that our methods, while derived in the case of constant birth and death rates, may be more broadly applicable.

1.2 Internal and external branch lengths

Here, we state our main limit theorem, which describes the lengths of the internal and external branches for the genealogical tree of a birth-death process when T and n are large. The asymptotic distribution of the internal branch lengths can be used to estimate the net growth rate of a clone, while the ratio of external to internal branch lengths could provide an estimate of the clone age when the growth rate is known, as detailed further in Supplementary section 1.4.

We call a branch of the genealogical tree internal if it is ancestral to between 2 and $n-1$ of the n leaves (red edges in Main text figure 1C-D) and external if it is ancestral to only one of the n leaves (blue edges in Main text figure 1C-D). Note that a mutation along an internal branch will be inherited by more than one of the sampled individuals, while a mutation along an external branch will be unique to one of the sampled individuals. Therefore, one can estimate the internal and external branch lengths from the number of shared and private mutations respectively.

The site frequency spectrum and the allele frequency spectrum have previously been studied for populations whose genealogy is described by a coalescent point process[8–12]. Because the internal and external branch lengths are closely related to the site frequency spectrum, our methods for using the coalescent point process to understand the internal and external branch lengths are similar to the methods used in these previous works. However, these earlier results are applicable when we are interested in the site frequency spectrum of the entire population, or when each individual is sampled independently with some probability p. Our result pertains to the case of a sample of fixed size n from a much larger population, leading to a star-shaped genealogical tree with long external branches in which most of the coalescence occurs near the root of the tree.

The asymptotic distribution for the internal and external branch lengths was obtained for the classical Kingman's coalescent[13] and for for coalescents with multiple mergers[14,15]. Recently, an asymptotic result for external branch lengths in Yule trees was proved[16]. However, as far as we know, such results have not previously been established for a sample of size n from a birth-death tree.

To state our theorem, we need to consider a sequence of birth-death processes indexed by the sample size n, and the time at which the sample is taken, which we will now denote by T_n, must tend to infinity with n. We will also write λ_n, μ_n, r_n, and ν_n for the birth, death, growth, and mutation rates respectively to emphasize that we allow them to depend on n. We will let L_n^{in} and L_n^{ex} denote the total length of all internal and external branches respectively in the genealogical tree.

Theorem 1. *Suppose*

$$\lim_{n\to\infty} ne^{-r_n T_n} = 0. \tag{11}$$

Then, using $\xrightarrow{P}$ to denote convergence in probability as $n \to \infty$, we have

$$\frac{r_n L_n^{in}}{n} \xrightarrow{P} 1. \tag{12}$$

Furthermore, suppose instead we have

$$\lim_{n\to\infty} n^{3/2}(\log n)e^{-r_n T_n} = 0. \tag{13}$$

Let Z have a standard normal distribution, and let W have an exponential distribution with mean 1, independent of Z. Then

$$\left(\frac{r_n}{\sqrt{n}}\left(L_n^{in} - \frac{n}{r_n}\right), \frac{r_n}{n}L_n^{ex} - r_n T_n + \log n + 1\right) \Rightarrow (Z, \log W), \tag{14}$$

where $\Rightarrow$ denotes convergence in distribution as $n \to \infty$.

Recall that the expected population size at time T_n is $e^{r_n T_n}$, so the condition (11) means that the sample size n must be much smaller than the population size. Under this condition, the total internal branch length L_n^{in} is close to n/r_n with high probability, which means the growth rate estimate (main text equation 4) should be accurate. Under the stronger condition (13), the distribution of the total internal branch length L_n^{in} is approximately normal with mean n/r_n and standard deviation $\sqrt{n}/r_n$, which we denote by

$$L_n^{in} \sim \mathcal{N}(n/r_n, \sqrt{n}/r_n)\,. \tag{15}$$

This means that the confidence interval in (main text equation 5) should be accurate.

Because $\mathbb{E}[\log W] = -\gamma$, where $\gamma \approx .577$ is Euler's constant, Theorem 1 also suggests that for the total external branch length,

$$\mathbb{E}[L_n^{ex}] \approx nT_n - \frac{n}{r_n}(\log n + 1 - \mathbb{E}[\log W]) = nT_n - \frac{n}{r_n}(\log n + 1 + \gamma). \tag{16}$$

1.3 Shared and private mutations

Let M_n^{in} denote the number of mutations that appear on two or more of the sampled individuals, and let M_n^{ex} denote the number of mutations that appear on only one of the sampled individuals. Because we are assuming that mutations occur along each lineage at rate ν_n, the conditional distribution of M_n^{in} given L_n^{in} is Poisson with mean $\nu_n L_n^{in}$, and likewise for M_n^{ex}. In particular, we have

$$\mathbb{E}[M_n^{in}] = \nu_n \mathbb{E}[L_n^{in}] \approx \frac{n\nu_n}{r_n}. \tag{17}$$

and, using the conditional variance formula,

$$\text{Var}(M_n^{in}) = \mathbb{E}[\text{Var}(M_n^{in}|L_n^{in})] + \text{Var}(\mathbb{E}[M_n^{in}|L_n^{in}]) = \nu_n \mathbb{E}[L_n^{in}] + \nu_n^2 \text{Var}(L_n^{in}).$$

Note that the approximation for $\mathbb{E}[M_n^{in}]$ is consistent with main text Eq. 8 because $\sum_{k=2}^{\infty} 1/(k(k-1)) = 1$. The following corollary to Theorem 1 shows that M_n^{in} has an asymptotically normal distribution.

Corollary 2. *Suppose that* (13) *holds and that*

$$\lim_{n\to\infty} \frac{\nu_n n}{r_n} = \infty. \tag{18}$$

Let

$$\sigma_n^2 = n\left(\frac{\nu_n}{r_n} + \frac{\nu_n^2}{r_n^2}\right).$$

Let Z have a standard normal distribution. Then

$$\frac{1}{\sigma_n}\left(M_n^{in} - \frac{n\nu_n}{r_n}\right) \Rightarrow Z,$$

where $\Rightarrow$ denotes convergence in distribution as $n \to \infty$.

Also, using (16) we have the approximation

$$\mathbb{E}[M_n^{ex}] \approx n\nu_n T_n - \frac{n\nu_n}{r_n}(\log n + 1 + \gamma). \tag{19}$$

A similar formula was derived by Durrett[17]. For the private mutations M_n^{ex}, depending on the mutation rate, the dominant source of fluctuations could either be the Gaussian fluctuations from the mutations process or the non-Gaussian fluctuations from the random variable W.

1.4 Estimating the clone age

While we did not find a dataset appropriate for applying our method of estimating the clone age (i.e., time from clone initiation to time of sampling), we show here how it can be done when the growth rate of a clone is known and the mutation rate is unknown. If the mutation rate is known, then estimating the clone age is straightforward because we can simply divide the average number of mutations on the sampled individuals by the mutation rate. We therefore focus on how to estimate the clone age when the growth rate is known but the mutation rate is unknown. Note first that it is not possible to make such an estimate by using only the shared mutations. To see this, consider the figure below in which the dots represent mutations. Because the genealogical tree is nearly star-shaped, we will see the same shared mutations regardless of whether we take the sample at time $T/2$ or time T. The only difference is that if the sample is taken at time T, we will see more private mutations. We therefore estimate the tumor age by comparing the number of shared mutations to the number of private mutations. From (17) and (19), a natural estimate of the age of the tumor is

$$\hat{T} = \frac{M_n^{ex}}{rM_n^{in}} + \frac{\log n + 1 + \gamma}{r}.$$

The result below establishes some asymptotic properties of this estimate, and shows how to obtain a

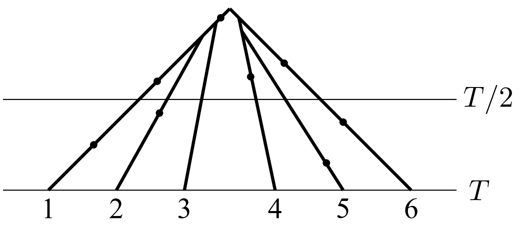

Supplementary Figure 1: Six lineages sampled at times $T/2$ and T

confidence interval for T. Condition (20) is needed to ensure that the Gaussian fluctuations from the lengths of the internal branches and the mutations process are the dominant source of fluctuations, rather than the non-Gaussian fluctuations from the random variable W that measures the initial growth of the branching process.

Corollary 3. *Suppose the following conditions hold:*

$$\lim_{n\to\infty} \frac{\log n}{r_n T_n} = 0, \qquad \lim_{n\to\infty} \frac{\nu_n n}{r_n} = \infty, \qquad \lim_{n\to\infty} \frac{n\nu_n}{r_n^2(r_n+\nu_n)T_n^2} = 0. \tag{20}$$

Define

$$\hat{T}_n = \frac{M_n^{ex}}{r_n M_n^{in}} + \frac{\log n + 1 + \gamma}{r_n},$$

as introduced above. Let Z have a standard normal distribution. Then

$$\frac{1}{T_n}\sqrt{\frac{n\nu_n}{r_n+\nu_n}}\left(\hat{T}_n - T_n\right) \Rightarrow Z.$$

From this result, one can show that for $0 < \alpha < 1$, an asymptotically valid $100(1-\alpha)\%$ confidence interval for T_n can be obtained by

$$\left[\frac{\hat{T}_n}{1+\frac{z_{\alpha/2}}{\sqrt{n}}\sqrt{1+\frac{n}{M_n^{in}}}}, \frac{\hat{T}_n}{1-\frac{z_{\alpha/2}}{\sqrt{n}}\sqrt{1+\frac{n}{M_n^{in}}}}\right].$$

2 Supplementary Information - Proofs

2.1 Approximating the coalescent point process

Recall that we are considering a continuous-time birth-death process in which each individual gives birth at rate λ and dies at rate μ, and we let $r = \lambda - \mu$ be the growth rate of the process. We assume $r > 0$. According to Theorem 3 and Corollary 4 of[1], we can construct the genealogy of a sample of size n from this process at time T, conditioned on the population having size at least n at time T, by first defining the coalescence times $H_1, \dots, H_{n-1}$ and then using the coalescent point process to obtain the genealogical tree, as described in Figure 1. Recall that we can obtain the random variables $H_1, \dots, H_{n-1}$ in the following way, where we record the dependence on n and T in the notation:

1. Choose a random variable $Y_{n,T}$ with probability density function on $(0,1)$ given by

$$f_{Y_{n,T}}(y) = \frac{n\delta_T y^{n-1}}{(y+\delta_T - y\delta_T)^{n+1}}, \qquad \delta_T = \frac{re^{-rT}}{\lambda(1-e^{-rT})+re^{-rT}}.$$

2. Conditional on $Y_{n,T} = y$, let the random variables $H_{i,n,T}$ for $1 \leq i \leq n-1$ be i.i.d. with probability density function on $(0,T)$ given by

$$f_{H_{i,n,T}|Y_{n,T}=y}(t) = \frac{y\lambda + (r-y\lambda)e^{-rT}}{y\lambda(1-e^{-rT})} \cdot \frac{y\lambda r^2 e^{-rt}}{(y\lambda+(r-y\lambda)e^{-rt})^2}.$$

Our goal is to take a limit as $T \to \infty$ and then as $n \to \infty$ to obtain the approximation to the coalescent point process that was described in the section Approximating genealogy using a coalescent point process. We first make a change of variables. We can write $Y_{n,T}$ as $\delta_T \cdot Q_{n,T}$, where $Q_{n,T}$ has density

$$f_{Q_{n,T}}(q) = \frac{nq^{n-1}}{(1+q-q\delta_T)^{n+1}}, \quad q \in (0, 1/\delta_T).$$

Let

$$G_{i,n,T} = T - H_{i,n,T}.$$

Note that $G_{i,n,T}$ represents the amount of time, after time zero, that the coalescence event occurred, whereas $H_{i,n,T}$ represents the amount of time, before the sampling time, that the coalescence event occurred. It will sometimes be more convenient to work with the times $G_{i,n,T}$. Replacing y by $\delta_T \cdot q$, the density for $G_{i,n,T}$ given $Q_{n,T} = q$ is, for $0 < t < T$,

$$f_{G_{i,n,T}|Q_{n,T}=q}(t) = \frac{q\delta_T\lambda + (r-q\delta_T\lambda)\,e^{-rT}}{q\delta_T\lambda\,(1-e^{-rT})} \cdot \frac{q\delta_T\lambda r^2 e^{-r(T-t)}}{\left(q\delta_T\lambda + (r-q\delta_T\lambda)\,e^{-r(T-t)}\right)^2}.$$

By writing $U_{i,n,T} = rG_{i,n,T} - \log q$, we have, for $-\log q < u < rT - \log q$,

$$\begin{aligned} f_{U_{i,n,T}|Q_{n,T}=q}(u) &= \frac{q\delta_T\lambda + (r-q\delta_T\lambda)\,e^{-rT}}{q\delta_T\lambda\,(1-e^{-rT})} \cdot \frac{q\delta_T\lambda r^2 e^{-rT} q e^u}{\left(q\delta_T\lambda + (r-q\delta_T\lambda)\,e^{-rT} q e^u\right)^2} \cdot \frac{1}{r} \\ &= \frac{q\delta_T\lambda + (r-q\delta_T\lambda)\,e^{-rT}}{q\delta_T\lambda(1-e^{-rT})} \cdot \frac{\delta_T\lambda r e^{-rT} e^u}{(\delta_T\lambda + (r-q\delta_T\lambda)\,e^{-rT}e^u)^2}. \end{aligned}$$

Thus, to obtain the coalescence times $H_{i,n,T}$, we can:

1. Choose $Q_{n,T}$ from the density

$$f_{Q_{n,T}}(q) = \frac{nq^{n-1}}{(1+q-q\delta_T)^{n+1}}, \quad q \in (0, 1/\delta_T).$$

2. Given $Q_{n,T} = q$, sample $\{U_{i,n,T}\}_{i=1}^{n-1}$ i.i.d. from the density

$$f_{U_{i,n,T}|Q_{n,T}=q}(u) = \frac{q\delta_T\lambda + (r - q\delta_T\lambda)\, e^{-rT}}{q\delta_T\lambda\,(1 - e^{-rT})} \cdot \frac{\delta_T\lambda r e^{-rT} e^u}{(\delta_T\lambda + (r - q\delta_T\lambda)\, e^{-rT} e^u)^2},$$
$$u \in (-\log q, rT - \log q).$$

3. Let $H_{i,n,T} = T - \frac{1}{r}\left(\log Q_{n,T} + U_{i,n,T}\right)$.

We now take a limit as $T \to \infty$. We note that the $T \to \infty$ limit was previously considered, in a more general setting allowing for non-binary branching, in[7,18]. For fixed λ, we have $\delta_T \to 0$ as $rT \to \infty$. Therefore, the density $f_{Q_{n,T}}(q)$ converges pointwise to the density

$$f_{Q_{n,\infty}}(q) = \frac{nq^{n-1}}{(1+q)^{n+1}}, \quad q \in (0, \infty).$$

By the definition of δ_T, for fixed λ, we have $\delta_T\lambda \sim re^{-rT}$ as rT goes to ∞, where $\sim$ means that the ratio of the two sides tends to 1. Therefore, the conditional density $f_{U_{i,n,T}|Q_{n,T}=q}$ converges pointwise to

$$f_{U_{i,n,\infty}|Q_{n,\infty}=q}(u) = \frac{q+1}{q} \cdot \frac{e^u}{(1+e^u)^2}, \quad u \in (-\log q, \infty).$$

Therefore, to obtain approximate coalescence times $H_{i,n,\infty}$ for $1 \le i \le n-1$, we can

1. Choose $Q_{n,\infty}$ from the density

$$f_{Q_{n,\infty}}(q) = \frac{nq^{n-1}}{(1+q)^{n+1}}dq, \quad q \in (0, \infty),$$

2. Given $Q_{n,\infty} = q$, sample $\{U_{i,n,\infty}\}_{i=1}^{n}$ i.i.d. from the density

$$f_{U_{i,n,\infty}|Q_{n,\infty}=q}(u) = \frac{q+1}{q} \cdot \frac{e^u}{(1+e^u)^2}, \quad u \in (-\log q, \infty),$$

3. Let $H_{i,n,\infty} = T - \frac{1}{r}\left(\log Q_{n,\infty} + U_{i,n,\infty}\right)$.

Next, we take a limit as $n \to \infty$. Let $\widetilde{Q}_{n,\infty} = Q_{n,\infty}/n$, which has density

$$f_{\widetilde{Q}_{n,\infty}}(q) = \frac{q^{n-1}}{\left(\frac{1}{n} + q\right)^{n+1}}, \quad q \in (0, \infty).$$

As n goes to infinity, $f_{\widetilde{Q}_{n,\infty}}(q)$ converges pointwise to

$$\lim_{n\to\infty} \frac{q^{n-1}}{\left(\frac{1}{n} + q\right)^{n+1}} = \frac{e^{-1/q}}{q^2}, \quad q \in (0, \infty),$$

which is the density function for $1/W$ where W has an exponential distribution with rate 1. Condi-

tional on $\widetilde{Q}_{n,\infty} = q$, the density of $U_{i,n,\infty}$ for $1 \le i \le n-1$ is

$$f_{U_{i,n,\infty}|\widetilde{Q}_{n,\infty}=q}(u) = \frac{nq+1}{nq} \cdot \frac{e^u}{(1+e^u)^2}, \quad u \in (-\log q - \log n, \infty)\,.$$

For each $q > 0$, as n goes to infinity, $f_{U_{i,n,\infty}|\widetilde{Q}_{n,\infty}=q}(u)$ converges pointwise to

$$\lim_{n\to\infty} f_{U_{i,\infty,\infty}|\widetilde{Q}_{n,\infty}=q}(u) = \frac{e^u}{(1+e^u)^2}, \quad u \in (-\infty, \infty),$$

which is the density function for the standard logistic distribution. Therefore, when both T and n are large, the coalescence times $H_{i,n,T}$ can be well approximated in the following way, which was described the section Approximating genealogy using a coalescent point process. Note that here, and frequently in the rest of the document, we write U_i in place of $U_{i,\infty,\infty}$ to lighten notation.

1. Let W have an exponential distribution with mean one.
2. Let $\{U_i\}_{i=1}^{n-1}$ be i.i.d. random variables with density

$$f_{U_i}(u) = \frac{e^u}{(1+e^u)^2}, \quad u \in (-\infty, \infty)$$

3. Let

$$H_i = T - \frac{1}{r}\Big(\log(1/W) + \log n + U_i\Big).$$

2.2 Error bounds

In this section, we will show that the random variables H_i give good approximations to the random variables $H_{i,n,T}$. Recall that $G_{i,n,T} = T - H_{i,n,T}$, and likewise define $G_{i,n,\infty} = T - H_{i,n,\infty}$ and $\widetilde{G}_{i,n,\infty} = T - H_i$. It then suffices to compare the random variables $\widetilde{G}_{i,n,\infty}$ and $G_{i,n,T}$. We will always assume that $n < e^{rT}$, which holds for sufficiently large n when (13) is satisfied.

To show that the random variables $\widetilde{G}_{i,n,\infty}$ give a good approximation to the random variables $G_{i,n,T}$, we will use the technique of coupling. That is, we will construct these random variables on the same probability space in such a way that the absolute value of their difference is small with high probability. More precisely, suppose a random variable X has distribution μ_X, and a random variable Y has distribution μ_Y. When we say that the random variables X and Y can be coupled so that a certain condition holds, we mean that on some probability space, we can define random variables X' and Y' such that X' has distribution μ_X, Y' has distribution μ_Y, and the random variables (X', Y') satisfy the given condition.

We will often use what is known as the maximal coupling (see section 4.4 of chapter 1 of[19]). If X and Y are random variables with probability density functions f and g respectively, then the random variables X and Y can be coupled so that $P(X = Y) = \int_{-\infty}^{\infty} f(x) \wedge g(x)\, dx$ and $P(X \neq Y) = \frac{1}{2}\int_{-\infty}^{\infty} |f(x) - g(x)|\, dx$.

Throughout this section, C will be some constant varying from line to line, independent of the parameters n, T, λ and μ. By $f(x) = g(x)(1 + O(h(x)))$, we mean $|f(x) - g(x)| \le Cg(x)h(x)$.

Lemma 4. *Suppose $n \le h(n) \le e^{rT-1}$. Then we can couple $Q_{n,T}$ and $Q_{n,\infty}$ so that*

$$\mathbb{P}(Q_{n,T} \neq Q_{n,\infty}) \le Cne^{-rT}, \qquad \mathbb{P}(Q_{n,T} = Q_{n,\infty} > h(n)) \le \frac{n}{h(n)},$$

and we can also couple $U_{i,n,T}$ and $U_{i,n,\infty}$ so that

$$\mathbb{E}\left[|U_{i,n,T} - U_{i,n,\infty}| \mathbb{1}_{\{Q_{n,T}=Q_{n,\infty} \le h(n)\}}\right] \le \frac{C\log\left(e^{rT}/h(n)\right)}{e^{rT}/h(n)}.$$

Proof. We can couple $Q_{n,T}$ and $Q_{n,\infty}$ so that

$$\mathbb{P}\left(Q_{n,T} = Q_{n,\infty}\right) = \int_0^\infty f_{Q_{n,T}}(q) \wedge f_{Q_{n,\infty}}(q)\, dq.$$

Likewise, on the event $\{Q_{n,T} = Q_{n,\infty} = q\}$, we can couple $U_{i,n,T}$ and $U_{i,n,\infty}$ so that

$$\mathbb{P}\left(U_{i,n,T} = U_{i,n,\infty} \mid Q_{n,T} = Q_{n,\infty} = q\right) = \int_0^\infty f_{U_{i,n,T}|Q_{n,T}=q}(u) \wedge f_{U_{i,n,\infty}|Q_{n,\infty}=q}(u)\, du.$$

On the event $\{Q_{n,T} \neq Q_{n,\infty}\}$, we take $U_{i,n,T}$ and $U_{i,n,\infty}$ to be arbitrary random variables with the prescribed conditional densities.

Since $\delta_T = \frac{r}{\lambda}e^{-rT}(1 + O(e^{-rT}))$, we have

$$\frac{nq^{n-1}}{(1+q-q\delta_T)^{n+1}} = \frac{nq^{n-1}}{(1+q)^{n+1}}\left(1 + O\left(n\delta_T\right)\right), \tag{21}$$

$$\frac{q\delta_T\lambda + (r - q\delta_T\lambda)\, e^{-rT}}{q\delta_T\lambda\left(1 - e^{-rT}\right)} = \frac{q+1}{q}\left(1 + O\left(e^{-rT}\right)\right), \tag{22}$$

$$\frac{\delta_T\lambda r e^{-rT}e^u}{\left(\delta_T\lambda + (r - q\delta_T\lambda)\, e^{-rT}e^u\right)^2} = \frac{e^u}{(1+e^u)^2}\left(1 + O\left((q+1)e^{-rT}\right)\right). \tag{23}$$

Using (21), we have

$$\begin{aligned}
\mathbb{P}(Q_{n,T} \neq Q_{n,\infty}) &= \int_0^\infty \frac{1}{2}\left|f_{Q_{n,\infty}}(q) - f_{Q_{n,T}}(q)\right| dq \\
&\le Cn\delta_T \int_0^{1/\delta_T} \frac{nq^{n-1}}{(1+q)^{n+1}}\, dq + \int_{1/\delta_T}^\infty \frac{nq^{n-1}}{(1+q)^{n+1}}\, dq \\
&\le Cn\delta_T + \int_{1/\delta_T}^\infty \frac{n}{q^2}\, dq \\
&\le Cne^{-rT}.
\end{aligned}$$

Also

$$\mathbb{P}(Q_{n,T} = Q_{n,\infty} > h(n)) \le \mathbb{P}(Q_{n,\infty} > h(n)) = \int_{h(n)}^{\infty} \frac{nq^{n-1}}{(1+q)^{n+1}}\, dq \le \int_{h(n)}^{\infty} \frac{n}{q^2}\, dq = \frac{n}{h(n)}.$$

Because $\lambda \ge r$, we have

$$\frac{1}{\delta_T} = \frac{\lambda(1-e^{-rT}) + re^{-rT}}{re^{-rT}} = \frac{\lambda}{r}(e^{rT}-1) + 1 \ge e^{rT} \ge h(n),$$

and therefore $q \le 1/\delta_T$ whenever $q \le h(n)$. Also, for $q \le h(n)$, we have $e^{rT}/q = e$. It follows that on the event $Q_{n,T} = Q_{n,\infty} = q$, by (22) and (23) we have for $q \le h(n)$,

$$\begin{aligned}
&\mathbb{E}\left[|U_{i,n,T} - U_{i,n,\infty}| \,\middle|\, Q_{n,T} = Q_{n,\infty} = q \right] \\
&\le \mathbb{E}\left[(|U_{i,n,T}| + |U_{i,n,\infty}|)\, \mathbb{1}_{\{U_{i,n,T} \neq U_{i,n,\infty}\}} \,\middle|\, Q_{n,T} = Q_{n,\infty} = q \right] \\
&= \int_{-\infty}^{\infty} |u| \left| f_{U_{i,n,T}|Q_{n,T}=q}(u) - f_{U_{i,n,\infty}|Q_{n,\infty}=q}(u) \right| \, du \\
&\le \int_{-\log q}^{rT-\log q} |u| C(q+1)e^{-rT} \cdot \frac{q+1}{q} \cdot \frac{e^u}{(1+e^u)^2}\, du + \int_{rT-\log q}^{\infty} |u| \cdot \frac{q+1}{q} \cdot \frac{e^u}{(1+e^u)^2}\, du \\
&\le C\left(\frac{(q+1)^2}{q} e^{-rT} + \frac{q+1}{q} \cdot \frac{\log\left(e^{rT}/q\right)}{e^{rT}/q} \right).
\end{aligned} \tag{24}$$

Since the function $f(x) = (\log x)/x$ is decreasing on (e, ∞), it follows that

$$\frac{\log\left(e^{rT}/q\right)}{e^{rT}/q} \le \frac{\log\left(e^{rT}/h(n)\right)}{e^{rT}/h(n)}, \qquad \text{for } q \le h(n).$$

Using this fact and integrating (24), we have

$$\begin{aligned}
&\mathbb{E}\left[|U_{i,n,T} - U_{i,n,\infty}|\, \mathbb{1}_{\{Q_{n,T} = Q_{n,\infty} \le h(n)\}} \right] \\
&\le \int_0^{h(n)} C\left(\frac{(q+1)^2}{q} e^{-rT} + \frac{q+1}{q} \cdot \frac{\log\left(e^{rT}/q\right)}{e^{rT}/q} \right) \frac{nq^{n-1}}{(1+q)^{n+1}}\, dq \\
&= C\int_0^{h(n)} (q+1)e^{-rT} \frac{nq^{n-2}}{(1+q)^n}\, dq + C\int_0^{h(n)} \frac{\log\left(e^{rT}/q\right)}{e^{rT}/q} \cdot \frac{nq^{n-2}}{(1+q)^n}\, dq \\
&\le C(h(n)+1)e^{-rT} \int_0^{\infty} \frac{nq^{n-2}}{(1+q)^n}\, dq + \frac{C\log\left(e^{rT}/h(n)\right)}{e^{rT}/h(n)} \int_0^{\infty} \frac{nq^{n-2}}{(1+q)^n}\, dq \\
&\le \frac{C\log\left(e^{rT}/h(n)\right)}{e^{rT}/h(n)},
\end{aligned}$$

which completes the proof. □

Lemma 5. *We can couple $\widetilde{Q}_{n,\infty}$ with $1/W$ so that*

$$\mathbb{P}\left(\widetilde{Q}_{n,\infty} \neq 1/W \right) \le \frac{C}{n},$$

and we can also couple $U_{i,n,\infty}$ with $U_{i,\infty,\infty}$ so that

$$\mathbb{E}\left[|U_{i,n,\infty} - U_{i,\infty,\infty}|\mathbb{1}_{\{\widetilde{Q}_{n,\infty}=1/W\}}\right] \le \frac{C\log n}{n}.$$

Proof. We can couple $\widetilde{Q}_{n,\infty}$ with $1/W$ so that

$$\mathbb{P}\left(\widetilde{Q}_{n,\infty} = 1/W\right) = \int_0^\infty f_{\widetilde{Q}_{n,\infty}}(q) \wedge f_{1/W}(q)\, dq.$$

On the event $\{\widetilde{Q}_{n,\infty} = q\}$, we can couple $U_{i,n,\infty}$ and $U_{i,\infty,\infty}$ so that

$$\mathbb{P}\left(U_{i,n,\infty} = U_{i,\infty,\infty} \mid \widetilde{Q}_{n,\infty} = q\right) = \int_0^\infty f_{U_{i,n,\infty}|\widetilde{Q}_{n,\infty}=q}(u) \wedge f_{U_{i,\infty,\infty}}(u)\, du.$$

We start with a bound on $\left|f_{\widetilde{Q}_{n,\infty}}(q) - f_{1/W}(q)\right|$. Note that

$$\frac{f_{\widetilde{Q}_{n,\infty}}(q)}{f_{1/W}(q)} = \frac{q^{n-1}}{\left(\frac{1}{n}+q\right)^{n+1}} \bigg/ \frac{e^{-1/q}}{q^2} = \exp\left(\frac{1}{q} - (n+1)\log\left(1+\frac{1}{nq}\right)\right).$$

Take $x_1 = 0$, $x_2 = 1/q - (n+1)\log(1+1/nq)$, and $F(x) = e^x$ so that $f_{1/W}(q) = F(x_1)f_{1/W}(q)$ and $f_{\widetilde{Q}_{n,\infty}}(q) = F(x_2)f_{1/W}(q)$. Using the standard inequality that $\frac{x}{x+1} < \log(1+x) < x$ for $x > 0$, we have

$$-\frac{1}{nq} < \frac{1}{q} - (n+1)\log\left(1+\frac{1}{nq}\right) < \frac{1-q}{(nq+1)q} < \frac{1}{nq^2}.$$

In particular,

$$|x_2 - x_1| \le \frac{1}{nq} \vee \frac{1}{nq^2},$$

and

$$f_{\widetilde{Q}_{n,\infty}}(q) = F(x_2)f_{1/W}(q) \le e f_{1/W}(q) \qquad \text{for } q \ge \frac{1}{\sqrt{n}}. \tag{25}$$

Applying the mean value theorem, we have

$$\begin{aligned}
\left|f_{\widetilde{Q}_{n,\infty}}(q) - f_{1/W}(q)\right| &= f_{1/W}(q)|F(x_2) - F(x_1)| \\
&\le f_{1/W}(q)\,|F'(x_2) \vee F'(x_1)|\,|x_2 - x_1| \\
&= f_{1/W}(q)\,|F(x_2) \vee F(x_1)|\,|x_2 - x_1| \\
&\le \left|f_{\widetilde{Q}_{n,\infty}(q)} \vee f_{1/W}(q)\right| \left(\frac{1}{nq} \vee \frac{1}{nq^2}\right).
\end{aligned} \tag{26}$$

For $g(q)$ where $g(q)$ can be $1/q$ or $1/q^2$, by (25) we have

$$\begin{aligned}
&\int_0^\infty g(q)\left(f_{\widetilde{Q}_{n,\infty}}(q)\vee f_{1/W}(q)\right)\,dq\\
&\quad\le \int_0^\infty g(q)f_{1/W}(q)\,dq+\int_0^\infty g(q)f_{\widetilde{Q}_{n,\infty}}(q)\,dq\\
&\quad= \int_0^\infty g(q)f_{1/W}(q)\,dq+\int_0^{1/\sqrt{n}} g(q)f_{\widetilde{Q}_{n,\infty}}(q)\,dq+\int_{1/\sqrt{n}}^\infty g(q)f_{\widetilde{Q}_{n,\infty}}(q)\,dq\\
&\quad\le (1+e)\int_0^\infty g(q)f_{1/W}(q)\,dq+\int_0^{1/\sqrt{n}} g(q)f_{\widetilde{Q}_{n,\infty}}(q)\,dq. \qquad (27)
\end{aligned}$$

Since $g(q)f_{1/W}(q)$ is integrable on $(0,\infty)$ the first term in (27) is bounded above by some absolute constant. For the second term, using the fact that $q^2g(q)$ is integrable on $(0,1)$,

$$\begin{aligned}
\int_0^{1/\sqrt{n}} g(q)f_{\widetilde{Q}_{n,\infty}}(q)\,dq &= \int_0^{1/\sqrt{n}} g(q)\frac{q^{n-1}}{\left(\frac{1}{n}+q\right)^{n+1}}\,dq\\
&= \int_0^{1/\sqrt{n}} q^2g(q)\frac{q^{n-3}}{\left(\frac{1}{n}+q\right)^{n+1}}\,dq\\
&= \int_0^{1/\sqrt{n}} q^2g(q)\frac{n^4(nq)^{n-3}}{(1+nq)^{n+1}}\,dq\\
&\le \int_0^{1/\sqrt{n}} n^4q^2g(q)\left(\frac{nq}{1+nq}\right)^{n-3}\,dq\\
&\le \int_0^{1/\sqrt{n}} n^4q^2g(q)\left(1-\frac{1}{1+\sqrt{n}}\right)^{n-3}\,dq\\
&\le n^4\exp\left(-\frac{n-3}{1+\sqrt{n}}\right)\int_0^{1/\sqrt{n}} q^2g(q)\,dq\\
&\le C. \qquad (28)
\end{aligned}$$

By (26), (27), and (28), we have

$$\begin{aligned}
\mathbb{P}\big(\widetilde{Q}_{n,\infty}\ne 1/W\big) &= \frac{1}{2}\int_0^\infty \left|f_{\widetilde{Q}_{n,\infty}}(q)-f_{1/W}(q)\right|\,dq\\
&\le \int_0^\infty \left(f_{\widetilde{Q}_{n,\infty}}(q)\vee f_{1/W}(q)\right)\left(\frac{1}{nq}\vee\frac{1}{nq^2}\right)\,dq\\
&\le \frac{C}{n}. \qquad (29)
\end{aligned}$$

We also have

$$\begin{aligned}
&\mathbb{E}\left[|U_{i,n,\infty} - U_{i,\infty,\infty}|\, \mathbb{1}_{\{\tilde{Q}_{n,\infty}=1/W\}}\right] \\
&\le \int_0^\infty \int_0^\infty |u| \left| f_{U_{i,n,\infty}|\tilde{Q}_{n,\infty}=q}(u) - f_{U_{i,\infty,\infty}}(u) \right| \left(f_{1/W}(q) \wedge f_{\tilde{Q}_{n,\infty}}(q) \right) du\, dq \\
&\le \int_0^\infty \left(\int_{-\infty}^{-\log q - \log n} \frac{|u| e^u}{(1+e^u)^2}\, du + \int_{-\infty}^{\infty} |u| \frac{1}{nq} \frac{e^u}{(1+e^u)^2}\, du \right) \frac{e^{-1/q}}{q^2}\, dq \\
&= \int_0^\infty \int_{-\infty}^{-\log q - \log n} |u| \frac{e^u}{(1+e^u)^2} \frac{e^{-1/q}}{q^2}\, du\, dq + \int_0^\infty \int_{-\infty}^{\infty} |u| \frac{1}{nq} \frac{e^u}{(1+e^u)^2} \frac{e^{-1/q}}{q^2}\, du\, dq \\
&\le \int_{-\infty}^{\infty} \int_0^{1/(ne^u)} |u| \frac{e^u}{(1+e^u)^2} \frac{e^{-1/q}}{q^2}\, dq\, du + \frac{C}{n} \\
&= \int_{-\infty}^{\infty} |u| \frac{e^u}{(1+e^u)^2} e^{-ne^u}\, du + \frac{C}{n} \\
&\le \frac{C \log n}{n}. && (30)
\end{aligned}$$

The lemma follows from (29) and (30). □

For the rest of the proof, we will assume that we have a sequence of birth-death processes indexed by the sample size n, and we will denote the growth rate and the sampling time by r_n and T_n. The next lemma, which is proved using the previous two lemmas, establishes that the random variables G_{i,n,T_n} can be well approximated by the random variables $\widetilde{G}_{i,n,\infty}$.

Lemma 6. *Assume condition* (11) *holds. The random variables G_{i,n,T_n} and $\widetilde{G}_{i,n,\infty}$ can be coupled so that as $n \to \infty$,*

$$\frac{r_n}{n} \sum_{i=1}^{n-1} \left| G_{i,n,T_n} - \widetilde{G}_{i,n,\infty} \right| \xrightarrow{P} 0.$$

Assume condition (13) *holds. The random variables G_{i,n,T_n} and $\widetilde{G}_{i,n,\infty}$ can be coupled so that as $n \to \infty$,*

$$\frac{r_n}{\sqrt{n}} \sum_{i=1}^{n-1} \left| G_{i,n,T_n} - \widetilde{G}_{i,n,\infty} \right| \xrightarrow{P} 0.$$

Proof. Under condition (11), we take $h(n) = n^{1/2} e^{r_n T_n/2}$ in Lemma 4 so that

$$\lim_{n\to\infty} \frac{n}{h(n)} = \lim_{n\to\infty} \left(n e^{-r_n T_n} \right)^{1/2} = 0,$$

and

$$\lim_{n\to\infty} \frac{\log\left(e^{r_n T_n}/h(n) \right)}{e^{r_n T_n}/h(n)} = \lim_{n\to\infty} n^{1/2} e^{-r_n T_n/2} \log\left(n^{-1/2} e^{r_n T_n/2} \right).$$

Using condition (11) and the fact that $\lim_{x\to 0^+} x \log(1/x) = 0$, the limit above is 0. Then by Lemma 4, as $n \to \infty$ we have

$$\mathbb{P}(Q_{n,T_n} = Q_{n,\infty} \le h(n)) \to 1, \tag{31}$$

and

$$r_n \mathbb{E}\left[|G_{i,n,T_n} - G_{i,n,\infty}| \mathbb{1}_{\{Q_{n,T_n}=Q_{n,\infty}\le h(n)\}}\right]$$
$$= \mathbb{E}\left[|U_{i,n,T_n} - U_{i,n,\infty}| \mathbb{1}_{\{Q_{n,T_n}=Q_{n,\infty}\le h(n)\}}\right] \le \frac{C\log\left(e^{r_nT_n}/h(n)\right)}{e^{r_nT_n}/h(n)} \to 0.$$

Summing over $1 \le i \le n-1$ and applying Markov's inequality, we have

$$\frac{r_n}{n}\sum_{i=1}^{n-1} |G_{i,n,T_n} - G_{i,n,\infty}|\, \mathbb{1}_{\{Q_{n,T_n}=Q_{n,\infty}\le h(n)\}} \xrightarrow{P} 0. \tag{32}$$

Combining (31) and (32), we have

$$\frac{r_n}{n}\sum_{i=1}^{n-1} |G_{i,n,T_n} - G_{i,n,\infty}| \xrightarrow{P} 0. \tag{33}$$

A similar argument using Lemma 5 instead implies that

$$\frac{r_n}{n}\sum_{i=1}^{n-1} |G_{i,n,\infty} - \widetilde{G}_{i,n,\infty}| \xrightarrow{P} 0. \tag{34}$$

The first claim of the lemma follows from (33) and (34).

Under condition (13), we take $h(n) = n^{1/2}(\log n)^{-1/3}e^{r_nT_n/3}$ in Lemma 4 so that

$$\lim_{n\to\infty}\frac{n}{h(n)} = \lim_{n\to\infty}\left(n^{3/2}(\log n)e^{-r_nT_n}\right)^{1/3} = 0,$$

and

$$\lim_{n\to\infty}\sqrt{n}\cdot\frac{\log\left(e^{r_nT_n}/h(n)\right)}{e^{r_nT_n}/h(n)}$$
$$= \lim_{n\to\infty} n(\log n)^{-1/3}e^{-2r_nT_n/3}\log\left(n^{-1/2}(\log n)^{1/3}e^{2r_nT_n/3}\right)$$
$$= \lim_{n\to\infty} n(\log n)^{-1/3}e^{-2r_nT_n/3}\left\{\log\left(n^{-1}(\log n)^{1/3}e^{2r_nT_n/3}\right) + \log(n^{1/2})\right\}. \tag{35}$$

Since

$$\lim_{n\to\infty} n(\log n)^{-1/3}e^{-2r_nT_n/3} = \lim_{n\to\infty}\left(n^{3/2}\left(\log n\right)e^{-r_nT_n}\right)^{2/3}\cdot(\log n)^{-1} = 0,$$

and $\lim_{x\to 0^+} x\log(1/x) = 0$, we have

$$\lim_{n\to\infty} n(\log n)^{-1/3}e^{-2r_nT_n/3}\log\left(n^{-1}(\log n)^{1/3}e^{2r_nT_n/3}\right) = 0. \tag{36}$$

Also,

$$\lim_{n\to\infty} n(\log n)^{-1/3}e^{-2r_nT_n/3}\log(n^{1/2}) = \lim_{n\to\infty}\frac{1}{2}\left(n^{3/2}(\log n)e^{-r_nT_n}\right)^{2/3} = 0.$$

Combining this with (35) and (36), we deduce that

$$\lim_{n\to\infty} \sqrt{n} \cdot \frac{\log\left(e^{r_n T_n}/h(n)\right)}{e^{r_n T_n}/h(n)} = 0.$$

Then by Lemma 4, we have

$$\mathbb{P}(Q_{n,T_n} = Q_{n,\infty} \leq h(n)) \to 1, \tag{37}$$

and

$$\begin{aligned} &r_n\sqrt{n}\,\mathbb{E}\left[|G_{i,n,T_n} - G_{i,n,\infty}|\mathbb{1}_{\{Q_{n,T_n}=Q_{n,\infty}\leq h(n)\}}\right] \\ &\quad= \sqrt{n}\,\mathbb{E}\left[|U_{i,n,T_n} - U_{i,n,\infty}|\mathbb{1}_{\{Q_{n,T_n}=Q_{n,\infty}\leq h(n)\}}\right] \leq C\sqrt{n} \cdot \frac{\log\left(e^{r_n T_n}/h(n)\right)}{e^{r_n T_n}/h(n)} \to 0. \end{aligned}$$

Summing over $1 \leq i \leq n-1$ and applying Markov's inequality, we have

$$\frac{r_n}{\sqrt{n}} \sum_{i=1}^{n-1} |G_{i,n,T_n} - G_{i,n,\infty}|\, \mathbb{1}_{\{Q_{n,T_n}=Q_{n,\infty}\leq h(n)\}} \xrightarrow{P} 0. \tag{38}$$

Combining (37) and (38), we have

$$\frac{r_n}{\sqrt{n}} \sum_{i=1}^{n-1} |G_{i,n,T_n} - G_{i,n,\infty}| \xrightarrow{P} 0. \tag{39}$$

A similar argument using Lemma 5 instead implies that

$$\frac{r_n}{\sqrt{n}} \sum_{i=1}^{n-1} |G_{i,n,\infty} - \widetilde{G}_{i,n,\infty}| \xrightarrow{P} 0. \tag{40}$$

The second claim of the lemma follows from (39) and (40). □

2.3 Approximating internal and external branch lengths

In this section, we show how to use the approximation to the coalescent point process to estimate the moments of the internal and external branch lengths. We first explain how to read off the internal and external branch lengths from the coalescent point process. Consider the coalescent point process for which the coalescence times are given by H_{i,n,T_n}. The length of the internal portion of the 0th branch, denoted by L^{in}_{0,n,T_n}, is

$$L^{in}_{0,n,T_n} = \max_{1\leq i\leq n-1} H_{i,n,T_n} - H_{1,n,T_n} = G_{1,n,T_n} - \min_{1\leq i\leq n-1} G_{i,n,T_n}.$$

Note that the top portion of the 0th branch, which is ancestral to all n of the sampled individuals, is not counted towards the internal branch length. For $1 \leq i \leq n-2$, the length of the internal portion

of the ith branch, denoted by $L^{in}_{i,n,T}$, is

$$L^{in}_{i,n,T_n} = (H_{i,n,T_n} - H_{i+1,n,T_n})^+ = (G_{i+1,n,T_n} - G_{i,n,T_n})^+.$$

The $(n-1)$st branch is entirely external. The total internal branch length is therefore

$$L^{in}_n = \sum_{i=0}^{n-2} L^{in}_{i,n,T_n}. \tag{41}$$

The length of the external portion of the 0th branch is

$$L^{ex}_{0,n,T_n} = H_{1,n,T_n} = T_n - G_{1,n,T_n}.$$

For $1 \le i \le n-2$, the length of the external portion of the ith branch is

$$L^{ex}_{i,n,T_n} = H_{i,n,T_n} \wedge H_{i+1,n,T_n} = T_n - G_{i,n,T_n} \vee G_{i+1,n,T_n}.$$

The length of the external portion of the $(n-1)$st branch is

$$L^{ex}_{n-1,n,T_n} = H_{n-1,n,T_n} = T_n - G_{n-1,n,T_n}.$$

The total external branch length is

$$L^{ex}_n = \sum_{i=0}^{n-1} L^{ex}_{i,n,T_n}. \tag{42}$$

We collect here some results about the logistic distribution that we will need later. We refer the reader to[20]. See, in particular, the formula following (1.10) in[20] for the variance, and (2.3.9) of[20] for the formula for the expected value of the maximum of n i.i.d. logistic random variables.

Lemma 7. *Let $U_1, U_2, \ldots$ be i.i.d standard logistic random variables. Then $\mathbb{E}[U_1] = 0$ and $\mathrm{Var}(U_1) = \pi^2/3$. Also, we have*

$$\mathbb{E}\left[\max_{1 \le i \le n} U_i\right] = \sum_{i=1}^{n-1} \frac{1}{i}.$$

The next lemma shows that the internal and external branch lengths can be well approximated using the random variables $\widetilde{G}_{i,n,\infty}$ in place of G_{i,n,T_n}. For $1 \le i \le n-2$, we will use the notation

$$\widetilde{L}^{in}_{i,n,\infty} = (\widetilde{G}_{i+1,n,\infty} - \widetilde{G}_{i,n,\infty})^+$$

for the approximation to the length of the internal portion of the ith branch.

Lemma 8. *Assume condition (11) holds. Then as $n \to \infty$, the following hold:*

1. For the internal branch length, we have

$$\frac{r_n}{n}\left(\sum_{i=0}^{n-2} L^{in}_{i,n,T_n} - \sum_{i=1}^{n-2} \widetilde{L}^{in}_{i,n,\infty}\right) \xrightarrow{P} 0.$$

2. For the external branch length, we have

$$\frac{r_n}{n}\left(\sum_{i=0}^{n-1}(T_n - L^{ex}_{i,n,T_n}) - \sum_{i=1}^{n-2}(\widetilde{G}_{i,n,\infty} \vee \widetilde{G}_{i+1,n,\infty})\right) \xrightarrow{P} 0. \tag{43}$$

Assume condition (13) holds. Then as $n \to \infty$, the following hold:

1. For the internal branch length, we have

$$\frac{r_n}{\sqrt{n}}\left(\sum_{i=0}^{n-2} L^{in}_{i,n,T_n} - \sum_{i=1}^{n-2} \widetilde{L}^{in}_{i,n,\infty}\right) \xrightarrow{P} 0.$$

2. For the external branch length, we have

$$\frac{r_n}{\sqrt{n}}\left(\sum_{i=0}^{n-1}(T_n - L^{ex}_{i,n,T_n}) - \sum_{i=1}^{n-2}(\widetilde{G}_{i,n,\infty} \vee \widetilde{G}_{i+1,n,\infty})\right) \xrightarrow{P} 0. \tag{44}$$

Proof. The proofs under conditions (11) and (13) are essentially the same. We present the proof under condition (13) here. For the internal branch length, we have

$$\sum_{i=0}^{n-2} L^{in}_{i,n,T_n} - \sum_{i=1}^{n-2} \widetilde{L}^{in}_{i,n,\infty} = L^{in}_{0,n,T_n} + \sum_{i=1}^{n-2}\left(L^{in}_{i,n,T_n} - \widetilde{L}^{in}_{i,n,\infty}\right). \tag{45}$$

For the second term in (45), using the triangle inequality, we have

$$|L^{in}_{i,n,T_n} - \widetilde{L}^{in}_{i,n,\infty}| \leq |G_{i,n,T_n} - \widetilde{G}_{i,n,\infty}| + |G_{i+1,n,T_n} - \widetilde{G}_{i+1,n,\infty}|.$$

Summing this for $1 \leq i \leq n-2$ and using Lemma 6, we have

$$\frac{r_n}{\sqrt{n}}\sum_{i=1}^{n-2}\left(L^{in}_{i,n,T_n} - \widetilde{L}^{in}_{i,n,\infty}\right) \xrightarrow{P} 0. \tag{46}$$

For the first term in (45), we have

$$\begin{aligned} L^{in}_{0,n,T_n} &= G_{1,n,T_n} - \min_{1\leq i\leq n-1} G_{i,n,T_n} \\ &\leq \widetilde{G}_{1,n,\infty} - \min_{1\leq i\leq n-1} \widetilde{G}_{i,n,\infty} + |G_{1,n,T_n} - \widetilde{G}_{1,n,\infty}| + \sum_{i=1}^{n-1} |G_{i,n,T_n} - \widetilde{G}_{i,n,\infty}|. \end{aligned} \tag{47}$$

By Lemma 6, we have

$$\frac{r_n}{\sqrt{n}}\left(|G_{1,n,T_n}-\widetilde{G}_{1,n,\infty}|+\sum_{i=1}^{n-1}|G_{i,n,T_n}-\widetilde{G}_{i,n,\infty}|\right)\xrightarrow{P}0. \tag{48}$$

Finally, since U_i has a symmetric distribution, by Lemma 7, we have

$$\mathbb{E}\left[\widetilde{G}_{1,n,\infty}-\min_{1\le i\le n-1}\widetilde{G}_{i,n,\infty}\right]=\frac{1}{r_n}\mathbb{E}\left[U_1-\min_{1\le i\le n-1}U_i\right]=\frac{1}{r_n}\mathbb{E}\left[\max_{1\le i\le n-1}U_i\right]=\frac{1}{r_n}\sum_{i=1}^{n-2}\frac{1}{i}\le\frac{C\log n}{r_n}.$$

In particular, using Markov's Inequality, we have

$$\frac{r_n}{\sqrt{n}}\left(\widetilde{G}_{1,n,\infty}-\min_{1\le i\le n-1}\widetilde{G}_{i,n,\infty}\right)\xrightarrow{P}0. \tag{49}$$

The claim for the internal branch length follows from (45), (46), (47), (48), and (49).

For the external branch length, we have

$$\begin{aligned}\sum_{i=0}^{n-1}&(T_n-L^{ex}_{i,n,T_n})-\sum_{i=1}^{n-2}\left(\widetilde{G}_{i,n,\infty}\vee\widetilde{G}_{i+1,n,\infty}\right)\\&=G_{1,n,T_n}+\sum_{i=1}^{n-2}\Big((G_{i,n,T_n}\vee G_{i+1,n,T_n})-\big(\widetilde{G}_{i,n,\infty}\vee\widetilde{G}_{i+1,n,\infty}\big)\Big)+G_{n-1,n,T_n}.\end{aligned} \tag{50}$$

Similar to the argument for the internal branch length, we have

$$\frac{r_n}{\sqrt{n}}\sum_{i=1}^{n-2}\Big((G_{i,n,T_n}\vee G_{i+1,n,T_n})-\big(\widetilde{G}_{i,n,\infty}\vee\widetilde{G}_{i+1,n,\infty}\big)\Big)\xrightarrow{P}0. \tag{51}$$

Also, we have

$$\begin{aligned}\frac{r_n}{\sqrt{n}}\cdot G_{1,n,T_n}&\le\frac{r_n}{\sqrt{n}}\left(\widetilde{G}_{1,n,\infty}+|G_{1,n,T_n}-\widetilde{G}_{1,n,\infty}|\right)\\&\le\frac{1}{\sqrt{n}}\left(|\log(1/W)|+|U_1|+\log n+|G_{1,n,T_n}-\widetilde{G}_{1,n,\infty}|\right)\xrightarrow{P}0,\end{aligned} \tag{52}$$

and similarly for G_{n-1,n,T_n}. The claim for the external branch length follows from (50), (51), and (52). □

Lemma 9. *We have the following moment estimates for the internal branch length:*

$$\mathbb{E}\left[\sum_{i=1}^{n-2}\widetilde{L}^{in}_{i,n,\infty}\right]=\frac{1}{r_n}(n-2),$$

$$\mathrm{Var}\left(\sum_{i=1}^{n-2}\widetilde{L}^{in}_{i,n,\infty}\right)=\frac{1}{r_n^2}\left(n+\frac{\pi^2}{3}-4\right).$$

Proof. Since U_i has a symmetric distribution, we have

$$\mathbb{E}[U_i \wedge U_{i+1}] = \mathbb{E}[(-U_i) \wedge (-U_{i+1})] = -\mathbb{E}[U_i \vee U_{i+1}].$$

By Lemma 7, we have

$$\begin{aligned}\mathbb{E}\left[\left(\widetilde{G}_{i+1,n,\infty} - \widetilde{G}_{i,n,\infty}\right)^+\right] &= \frac{1}{r_n}\mathbb{E}\left[(U_{i+1} - U_i)^+\right] \\ &= \frac{1}{2r_n}\mathbb{E}[U_{i+1} \vee U_i - U_{i+1} \wedge U_i] = \frac{1}{r_n}\mathbb{E}[U_{i+1} \vee U_i] = \frac{1}{r_n}. \qquad (53)\end{aligned}$$

Therefore,

$$\mathbb{E}\left[\sum_{i=1}^{n-2} \widetilde{L}^{in}_{i,n,\infty}\right] = \frac{1}{r_n}(n-2).$$

For the variance computation, we have

$$\begin{aligned}\mathbb{E}\left[\left(\left(\widetilde{G}_{i+1,n,\infty} - \widetilde{G}_{i,n,\infty}\right)^+\right)^2\right] &= \frac{1}{r_n^2}\mathbb{E}\left[\left((U_{i+1} - U_i)^+\right)^2\right] \\ = \frac{1}{2r_n^2}\mathbb{E}\left[(U_{i+1} - U_i)^2\right] &= \frac{1}{2r_n^2}\text{Var}(U_{i+1} - U_i) = \frac{1}{r_n^2}\text{Var}(U_i) = \frac{\pi^2}{3r_n^2}\end{aligned}$$

and, using Mathematica to evaluate the triple integral,

$$\begin{aligned}&\mathbb{E}\left[\left(\widetilde{G}_{i+1,n,\infty} - \widetilde{G}_{i,n,\infty}\right)^+\left(\widetilde{G}_{i+2,n,\infty} - \widetilde{G}_{i+1,n,\infty}\right)^+\right] \\ &= \frac{1}{r_n^2}\mathbb{E}\left[(U_{i+1} - U_i)^+ (U_{i+2} - U_{i+1})^+\right] \\ &= \frac{1}{r_n^2}\int_{-\infty}^{\infty}\int_{u_i}^{\infty}\int_{u_{i+1}}^{\infty} (u_{i+1} - u_i)(u_{i+2} - u_{i+1})\frac{e^{u_i}}{(1+e^{u_i})^2}\frac{e^{u_{i+1}}}{(1+e^{u_{i+1}})^2}\frac{e^{u_{i+2}}}{(1+e^{u_{i+2}})^2}\, du_{i+2}\, du_{i+1}\, du_i \\ &= \left(2 - \frac{\pi^2}{6}\right)\frac{1}{r_n^2}.\end{aligned}$$

For i, j with $|i - j| \geq 2$, the random variables $(\widetilde{G}_{i+1,n,\infty} - \widetilde{G}_{i,n,\infty})^+$ and $(\widetilde{G}_{j+1,n,\infty} - \widetilde{G}_{j,n,\infty})^+$ are independent. Therefore, the variance of the internal branch length is given by:

$$\begin{aligned}\text{Var}\left(\sum_{i=1}^{n-2} \widetilde{L}^{in}_{i,n,\infty}\right) &= \sum_{i=1}^{n-2}\sum_{j=1}^{n-2} \text{Cov}\left(\widetilde{L}^{in}_{i,n,\infty}, \widetilde{L}^{in}_{j,n,\infty}\right) \\ &= (n-2)\text{Var}\left(\widetilde{L}^{in}_{1,n,\infty}\right) + 2(n-3)\text{Cov}\left(\widetilde{L}^{in}_{1,n,\infty}, \widetilde{L}^{in}_{2,n,\infty}\right) \\ &= (n-2)\left(\frac{\pi^2}{3r_n^2} - \frac{1}{r_n^2}\right) + 2(n-3)\left(\left(2 - \frac{\pi^2}{6}\right)\frac{1}{r_n^2} - \frac{1}{r_n^2}\right) \\ &= \frac{1}{r_n^2}\left(n + \frac{\pi^2}{3} - 4\right),\end{aligned}$$

which completes the proof. □

2.4 Proof of Theorem 1

Proof of Theorem 1. We first suppose that (11) holds. It follows from Lemma 8 that to prove (12), it suffices to show that

$$\frac{r_n}{n}\sum_{i=1}^{n-2}\widetilde{L}^{in}_{i,n,\infty}\xrightarrow{P}1. \tag{54}$$

By Lemma 9, we have

$$\mathbb{E}\left[\left(\frac{r_n}{n}\sum_{i=1}^{n-2}\widetilde{L}^{in}_{i,n,\infty}-1\right)^2\right]=\mathrm{Var}\left(\frac{r_n}{n}\sum_{i=1}^{n-2}\widetilde{L}^{in}_{i,n,\infty}\right)+\left(\frac{n-2}{n}-1\right)^2=\frac{1}{n^2}\left(n+\frac{\pi^2}{3}-4\right)+\frac{4}{n^2}.$$

The result (54) now follows from Chebyshev's inequality.

Next, we suppose that (13) holds, and we need to show (14). Taking the negative of the second coordinate in (14) and using (41) and (42), we see that (14) is equivalent to the convergence

$$\left(\frac{r_n}{\sqrt{n}}\left(\sum_{i=0}^{n-2}L^{in}_{i,n,T_n}-\frac{n}{r_n}\right),\frac{r_n}{n}\sum_{i=0}^{n-1}\left(T_n-L^{ex}_{i,n,T_n}\right)-\log n-1\right)\Rightarrow(Z,\log(1/W)).$$

By Lemma 8, it suffices to show that

$$\left(\frac{r_n}{\sqrt{n}}\left(\sum_{i=1}^{n-2}\widetilde{L}^{in}_{i,n,\infty}-\frac{n}{r_n}\right),\frac{r_n}{n}\sum_{i=1}^{n-2}\left(\widetilde{G}_{i,n,\infty}\vee\widetilde{G}_{i+1,n,\infty}\right)-\log n-1\right)\Rightarrow(Z,\log(1/W)). \tag{55}$$

Using (53), for $1\le i\le n-2$, we have

$$r_n\left(\widetilde{L}^{in}_{i,n,\infty}-\mathbb{E}\left[\widetilde{L}^{in}_{i,n,\infty}\right]\right)=r_n\left(\left(\widetilde{G}_{i+1,n,\infty}-\widetilde{G}_{i,n,\infty}\right)^+-\frac{1}{r_n}\right)=(U_{i+1}-U_i)^+-1.$$

Recall that a sequence of random variables $(X_i)_{i=1}^{\infty}$ is said to be m-dependent if $(X_i)_{i\le k}$ and $(X_i)_{i>k+m}$ are independent for all k. Note that the sequence whose ith term is $(U_{i+1}-U_i)^+-1$ is 1-dependent. We can therefore apply the Central Limit Theorem for m-dependent sequences to this sequence. By Theorem 3 of[21], it suffices to check the following conditions:

$$\liminf_{n\to\infty}\frac{1}{n}\mathrm{Var}\left(r_n\sum_{i=1}^{n-2}\widetilde{L}^{in}_{i,n,\infty}\right)>0,$$

$$\lim_{n\to\infty}\frac{1}{n}\sum_{i=1}^{n}\mathbb{E}\left[\left|r_n\widetilde{L}^{in}_{i,n,\infty}-\mathbb{E}\left[r_n\widetilde{L}^{in}_{i,n,\infty}\right]\right|^2\mathbb{1}_{\left\{\left|r_n\widetilde{L}^{in}_{i,n,\infty}-\mathbb{E}\left[r_n\widetilde{L}^{in}_{i,n,\infty}\right]\right|>\epsilon\sqrt{n}\right\}}\right]=0,\quad\forall\epsilon>0.$$

For the first condition, it follows from Lemma 9 that

$$\liminf_{n\to\infty}\frac{1}{n}\mathrm{Var}\left(r_n\sum_{i=1}^{n-2}\widetilde{L}^{in}_{i,n,\infty}\right)=1.$$

For the second condition, we have

$$\begin{aligned}
&\frac{1}{n}\sum_{i=1}^{n}\mathbb{E}\left[\left|r_n\widetilde{L}^{in}_{i,n,\infty}-\mathbb{E}\left[r_n\widetilde{L}^{in}_{i,n,\infty}\right]\right|^2\mathbb{1}_{\left\{\left|r_n\widetilde{L}^{in}_{i,n,\infty}-\mathbb{E}\left[r_n\widetilde{L}^{in}_{i,n,\infty}\right]\right|>\epsilon\sqrt{n}\right\}}\right]\\
&\qquad=\mathbb{E}\left[\left|r_n\widetilde{L}^{in}_{1,n,\infty}-\mathbb{E}\left[r_n\widetilde{L}^{in}_{1,n,\infty}\right]\right|^2\mathbb{1}_{\left\{\left|r_n\widetilde{L}^{in}_{1,n,\infty}-\mathbb{E}\left[r_n\widetilde{L}^{in}_{1,n,\infty}\right]\right|>\epsilon\sqrt{n}\right\}}\right]\\
&\qquad=\mathbb{E}\left[|(U_{1,\infty,\infty}-U_{2,\infty,\infty})^+-1|^2\mathbb{1}_{\left\{|(U_{1,\infty,\infty}-U_{2,\infty,\infty})^+-1|>\epsilon\sqrt{n}\right\}}\right],
\end{aligned}$$

which goes to 0 as n goes to infinity by the Dominated Convergence Theorem. Therefore, by the m-dependent Central Limit Theorem and Lemma 9,

$$\frac{r_n}{\sqrt{n}}\left(\sum_{i=1}^{n-2}\widetilde{L}^{in}_{i,n,\infty}-\frac{n}{r_n}\right)\Rightarrow Z, \tag{56}$$

where Z has a standard normal distribution.

We now consider the sum

$$\sum_{i=1}^{n-2}\left(\widetilde{G}_{i,n,\infty}\vee\widetilde{G}_{i+1,n,\infty}\right)=\frac{n-2}{r_n}\log(1/W)+\frac{n-2}{r_n}\log n+\frac{1}{r_n}\sum_{i=1}^{n-2}(U_i\vee U_{i+1}).$$

By Lemma 7, we have $\mathbb{E}(U_1\vee U_2)=1$. Applying the Strong Law of Large Numbers separately to $(U_1\vee U_2)+(U_3\vee U_4)+\dots$ and $(U_2\vee U_3)+(U_4\vee U_5)+\dots$ and then recombining, we have

$$\frac{r_n}{n}\sum_{i=2}^{n-2}\left(\widetilde{G}_{i,n,\infty}\vee\widetilde{G}_{i+1,n,\infty}\right)-\log n-1\to\log(1/W)\quad\text{a.s.} \tag{57}$$

Combining this result with (56) gives (55), which completes the proof. □

Remark 10. Note that the convergence of the second coordinate holds even if we only have (43) rather than (44) in Lemma 8. Therefore, even under the weaker condition (11), we have the convergence

$$\frac{r_n}{n}L^{ex}_n-r_nT_n+\log n+1\Rightarrow\log W.$$

2.5 Proof of Corollaries 2 and 3

To prove Corollaries 2 and 3, we need to consider the mutations. Let M^{in}_n be the number of mutations inherited by between 2 and $n-1$ individuals, and let M^{ex}_n be the number of mutations inherited by and exactly one individual. Recall that conditional on (L^{in}_n, L^{ex}_n), M^{in}_n and M^{ex}_n are independent Poisson random variables with means $\nu_nL^{in}_n$ and $\nu_nL^{ex}_n$ respectively. Therefore, there are two sources of fluctuations for M^{in}_n and M^{ex}_n. There are Poissonian fluctuations coming from the mutations process, and fluctuations resulting from the randomness in the branch lengths. We decompose and rescale the

fluctuations as follows. Let

$$A_n = \frac{M_n^{in} - \nu_n L_n^{in}}{\sqrt{\nu_n n / r_n}}, \qquad B_n = \frac{M_n^{ex} - \nu_n L_n^{ex}}{\sqrt{\nu_n n T_n}},$$
$$C_n = \frac{r_n}{\sqrt{n}} \left(L_n^{in} - \frac{n}{r_n} \right), \qquad D_n = \frac{r_n}{n} L_n^{ex} - r_n T_n + \log n + 1,$$

so that

$$M_n^{in} = \frac{\nu_n n}{r_n} + \sqrt{\frac{\nu_n n}{r_n}} A_n + \frac{\sqrt{n}\nu_n}{r_n} C_n, \tag{58}$$
$$M_n^{ex} = \nu_n n T_n - \frac{\nu_n n \log n}{r_n} - \frac{\nu_n n}{r_n} + \sqrt{\nu_n n T_n} B_n + \frac{\nu_n n}{r_n} D_n. \tag{59}$$

Corollaries 2 and 3 will follow from the next proposition, which says that (A_n, B_n, C_n, D_n) are asymptotically independent.

Proposition 11. *Suppose the following two conditions hold:*

$$\lim_{n\to\infty} \frac{\log n}{r_n T_n} = 0, \qquad \lim_{n\to\infty} \frac{\nu_n n}{r_n} = \infty. \tag{60}$$

Then as $n \to \infty$,

$$(A_n, B_n, C_n, D_n) \Rightarrow (Z_1, Z_2, Z, \log W), \tag{61}$$

where Z, Z_1, and Z_2 have a standard normal distribution, W has an exponential distribution with mean one, and Z, Z_1, Z_2, and W are independent. Furthermore, if the first condition in (60) is replaced by the weaker condition (13), then we still have the convergence

$$(A_n, C_n) \Rightarrow (Z_1, Z). \tag{62}$$

To prove Proposition 11, we will need the following lemma.

Lemma 12. *Let $(a_n)_{n=1}^\infty$, $(\delta_n)_{n=1}^\infty$, and $(\epsilon_n)_{n=1}^\infty$ be three sequences such that*

$$\lim_{n\to\infty} a_n = \infty, \qquad \lim_{n\to\infty} \frac{\delta_n}{a_n} = 0, \qquad \lim_{n\to\infty} \frac{\epsilon_n}{a_n} = 0.$$

Then for any $M > 0$ and $t \in \mathbb{R}$, we have as $n \to \infty$,

$$\sup_{-M \le c \le M} \left| \exp\left\{ (a_n + \delta_n + c\epsilon_n) \left(\exp\left(\frac{it}{\sqrt{a_n}} \right) - \frac{it}{\sqrt{a_n}} - 1 \right) \right\} - \exp\left(-\frac{t^2}{2} \right) \right| \to 0.$$

Proof. Using the inequality (see Lemma 3.3.19 of[22])

$$\left| e^{ix} - \sum_{m=0}^{n} \frac{(ix)^m}{m!} \right| \le \frac{|x|^{n+1}}{(n+1)!}, \tag{63}$$

we have

$$
\begin{aligned}
&\sup_{-M\le c\le M}\left|(a_n+\delta_n+c\epsilon_n)\left(\exp\left(\frac{it}{\sqrt{a_n}}\right)-\frac{it}{\sqrt{a_n}}-1\right)+\frac{t^2}{2}\right| \\
&\quad\le \sup_{-M\le c\le M}\left(\left|(a_n+\delta_n+c\epsilon_n)\left(-\frac{t^2}{2a_n}\right)+\frac{t^2}{2}\right|+|a_n+\delta_n+c\epsilon_n|\cdot\frac{1}{6}\left|\frac{it}{\sqrt{a_n}}\right|^3\right) \\
&\quad\le \frac{t^2(|\delta_n|+M|\epsilon_n|)}{2a_n}+\frac{t^3}{6}\left(\frac{|a_n|+|\delta_n|+M|\epsilon_n|}{a_n^{3/2}}\right),
\end{aligned}
$$

which goes to 0 as $n\to\infty$. The result follows because the exponential function is continuous. □

Proof of Proposition 11. We first prove (61) under the condition (60). We apply Theorem 3 of[23] where the marginal distributions are the distributions of (C_n, D_n) and the conditional distributions are the conditional distributions of (A_n, B_n) given (C_n, D_n). We know from Theorem 1 that $(C_n, D_n)\Rightarrow (Z,\log W)$. For $t=(t_1,t_2)\in\mathbb{R}^2$ and $(c,d)\in\mathbb{R}^2$, let

$$
\phi_n(t,(c,d))=\mathbb{E}\left[e^{it_1A_n+it_2B_n}|C_n=c,D_n=d\right],\quad \phi_0(t,(c,d))=\mathbb{E}\left[e^{it_1Z_1+it_2Z_2}\right].
$$

Note that $\phi_0(t,(c,d))$ does not depend on (c,d). We need to check the following:

1. For all compact sets $I\subset\mathbb{R}^2$, we have

$$
\sup_{(c,d)\in I}|\phi_n(t,(c,d))-\phi_0(t,(c,d))|\to 0 \text{ as } n\to\infty.
$$

2. The function $\phi_0(t,(c,d))$ is equicontinuous in (c,d) at $t=0$.
3. For each t, the function $\phi_0(t,(c,d))$ is a continuous function of (c,d).

The last two conditions are trivially satisfied because $\phi_0(t,(c,d))$ is constant as a function of (c,d). Now we check the first condition. Conditional on $C_n=c$ and $D_n=d$, which means

$$
L_n^{in}=\frac{n}{r_n}+\frac{c\sqrt{n}}{r_n},\qquad L_n^{ex}=nT_n-\frac{n\log n}{r_n}-\frac{n}{r_n}+\frac{dn}{r_n},
$$

the random variables (M_n^{in}, M_n^{ex}) are independent Poisson with means $\nu_nL_n^{in}$ and $\nu_nL_n^{ex}$ respectively. Therefore, we have

$$
\begin{aligned}
\phi_n(t,(c,d)) = &\exp\left\{\left(\frac{\nu_nn}{r_n}+\frac{c\nu_n\sqrt{n}}{r_n}\right)\left(\exp\left(it_1\sqrt{\frac{r_n}{\nu_nn}}\right)-it_1\sqrt{\frac{r_n}{\nu_nn}}-1\right)\right\}\\
&\times\exp\left\{\left(\nu_nnT_n-\frac{\nu_nn\log n}{r_n}-\frac{\nu_nn}{r_n}+\frac{d\nu_nn}{r_n}\right)\left(\exp\left(\frac{it_2}{\sqrt{\nu_nnT_n}}\right)-\frac{it_2}{\sqrt{\nu_nnT_n}}-1\right)\right\}
\end{aligned}
$$

and

$$
\phi_0(t,(c,d))=\exp\left(-\frac{t_1^2+t_2^2}{2}\right).
$$

Note that if $|z_i|\le 1$ and $|w_i|\le 1$ for $i=1,2$, then $|z_1z_2-w_1w_2|\le|z_1-w_1|+|z_2-w_2|$. It suffices

to check that for all $M > 0$, as $n \to \infty$, we have

$$\sup_{-M \le c \le M} \left| \exp\left\{ \left(\frac{\nu_n n}{r_n} + \frac{c\nu_n \sqrt{n}}{r_n} \right) \left(\exp\left(it_1 \sqrt{\frac{r_n}{\nu_n n}} \right) - it_1 \sqrt{\frac{r_n}{\nu_n n}} - 1 \right) \right\} - \exp\left(-\frac{t_1^2}{2} \right) \right| \to 0. \tag{64}$$

and

$$\sup_{-M \le d \le M} \left| \exp\left\{ \left(\nu_n n T_n - \frac{\nu_n n \log n}{r_n} - \frac{\nu_n n}{r_n} + \frac{d \nu_n n}{r_n} \right) \right. \right. \\ \left. \left. \times \left(\exp\left(\frac{it_2}{\sqrt{\nu_n n T_n}} \right) - \frac{it_2}{\sqrt{\nu_n n T_n}} - 1 \right) \right\} - \exp\left(-\frac{t_2^2}{2} \right) \right| \to 0. \tag{65}$$

Equations (64) and (65) follow from Lemma 12 and the assumptions in (60), which implies that (61) holds under the condition (60).

To prove (62) when the first condition in (60) is replaced by the weaker condition (13), we repeat the above argument using only A_n and C_n. It is then necessary to check only (64), which does not require the first condition in (60). □

Proof of Corollary 2. Note that, when we define $\sigma_n^2 = n(\nu_n/r_n + \nu_n^2/r_n^2)$ as in Corollary 2, we have

$$\frac{1}{\sigma_n}\left(M_n^{in} - \frac{n\nu_n}{r_n} \right) = \frac{1}{\sigma_n}\left(\sqrt{\frac{\nu_n n}{r_n}} A_n + \frac{\sqrt{n}\nu_n}{r_n} C_n \right).$$

Proposition 11 implies that the distribution of the right-hand side converges to the standard normal distribution as $n \to \infty$, which is the conclusion of Corollary 2. □

Proof of Corollary 3. From (58) and (59), we get

$$\begin{aligned} \hat{T}_n &= \frac{M_n^{ex}}{r_n M_n^{in}} + \frac{\log n + 1 + \gamma}{r_n} \\ &= \frac{n\nu_n T_n - n\nu_n(\log n)/r_n - n\nu_n/r_n + \sqrt{n\nu_n T_n} B_n + n\nu_n D_n/r_n}{n\nu_n + \sqrt{n\nu_n r_n} A_n + \sqrt{n}\nu_n C_n} + \frac{\log n + 1 + \gamma}{r_n} \\ &= \frac{T_n - (\log n)/r_n - 1/r_n + \sqrt{T_n/(n\nu_n)} B_n + D_n/r_n}{1 + \sqrt{r_n/(n\nu_n)} A_n + C_n/\sqrt{n}} + \frac{\log n + 1 + \gamma}{r_n}. \end{aligned} \tag{66}$$

For a sequence of random variables $(X_n)_{n=1}^\infty$ and a sequence of real numbers $(a_n)_{n=1}^\infty$, we write $X_n = o_p(a_n)$ if X_n/a_n converges to 0 in probability as $n \to \infty$. Since we are assuming (20), it follows that

$$\sqrt{r_n/(n\nu_n)} A_n = o_p(1), \qquad C_n/\sqrt{n} = o_p(1), \qquad (\log n + 1)/r_n = o_p(T_n)$$

$$\sqrt{T_n/(n\nu_n)} B_n = T_n \sqrt{1/(r_n T_n)} \sqrt{r_n/n\nu_n} B_n = o_p(T_n), \qquad D_n/r_n = o_p(T_n).$$

Therefore, writing E_n for an error term which is $o_p\big(\sqrt{r_n/(n\nu_n)} A_n + C_n/\sqrt{n}\big)$, we can rewrite (66)

as

$$\begin{aligned}\hat{T}_n &= \Big(T_n - (\log n)/r_n - 1/r_n + \sqrt{T_n/(n\nu_n)}B_n + D_n/r_n\Big) \\ &\quad \times \Big(1 - \sqrt{r_n/(n\nu_n)}A_n - C_n/\sqrt{n} + E_n\Big) + \frac{\log n + 1 + \gamma}{r_n} \\ &= T_n - \sqrt{r_n/(n\nu_n)}T_nA_n + \sqrt{T_n/(n\nu_n)}B_n - T_nC_n/\sqrt{n} + \frac{D_n+\gamma}{r_n} \\ &\quad + o_p\big(T_n\big(\sqrt{r_n/(n\nu_n)}A_n + C_n/\sqrt{n}\big)\big). \end{aligned} \tag{67}$$

Now we do a variance computation by replacing A_n, B_n, and C_n with Z_1, Z_2, and Z respectively. We have

$$\begin{aligned}&\mathrm{Var}\big(-\sqrt{r_n/(n\nu_n)}T_nZ_1 + \sqrt{T_n/(n\nu_n)}Z_2 - T_nZ/\sqrt{n}\big) \\ &\quad = \frac{r_nT_n^2}{n\nu_n} + \frac{T_n}{n\nu_n} + \frac{T_n^2}{n} = \frac{T_n}{n\nu_n}(r_nT_n + \nu_nT_n + 1) \sim \frac{(r_n+\nu_n)T_n^2}{n\nu_n},\end{aligned} \tag{68}$$

where $\sim$ means that the ratio of the two sides tends to 1 as $n \to \infty$. Equation (68) and Proposition 11 imply that the distribution of

$$\frac{1}{T_n}\sqrt{\frac{n\nu_n}{r_n+\nu_n}}\left(-\sqrt{r_n/(n\nu_n)}T_nA_n + \sqrt{T_n/(n\nu_n)}B_n - T_nC_n/\sqrt{n}\right)$$

converges to the standard normal distribution. It follows from (20) and Proposition 11 that

$$\frac{1}{T_n}\sqrt{\frac{n\nu_n}{r_n+\nu_n}} \cdot \frac{D_n+\gamma}{r_n} \Rightarrow 0.$$

Thus, the conclusion of Corollary 3 follows from (67). □

3 Supplementary Information – Derivation of Confidence Intervals

We explain in this section how our confidence intervals were derived.

3.1 Confidence interval for maximum likelihood

We assume here that we have random variables $H_i = a_n + b_nU_i$, where $U_1, \ldots, U_{n-1}$ are i.i.d. and have the standard logistic distribution. A package in R can then be used to obtain a maximum likelihood estimate $\hat{b}_n$ for b_n. It is known (see[24]) that when n is large, the estimate $\hat{b}_n$ is asymptotically normal, and the variance of $\hat{b}_n$ can be estimated using the Cramer-Rao bound, which gives

$$\mathrm{Var}(\hat{b}_n) \sim \frac{9}{3+\pi^2} \cdot \frac{b_n^2}{n}.$$

Therefore, letting $c = 3/\sqrt{3+\pi^2}$, we have

$$1-\alpha = \lim_{n\to\infty} \mathbb{P}\Big(b_n - z_{\alpha/2}\cdot\frac{cb_n}{\sqrt{n}} \le \hat{b}_n \le b_n + z_{\alpha/2}\cdot\frac{cb_n}{\sqrt{n}}\Big)$$
$$= \lim_{n\to\infty} \mathbb{P}\Big(b_n\Big(1 - z_{\alpha/2}\cdot\frac{c}{\sqrt{n}}\Big) \le \hat{b}_n \le b_n\Big(1 + z_{\alpha/2}\cdot\frac{c}{\sqrt{n}}\Big)\Big).$$

We now divide the inequality by $b_n\hat{b}_n$. Recalling that $r_n = 1/b_n$ and that the estimator for r_n can be expressed as $\hat{r}_n = 1/\hat{b}_n$, we arrive at

$$1-\alpha = \lim_{n\to\infty} \mathbb{P}\Big(\hat{r}_n\Big(1 - z_{\alpha/2}\cdot\frac{c}{\sqrt{n}}\Big) \le r_n \le \hat{r}_n\Big(1 + z_{\alpha/2}\cdot\frac{c}{\sqrt{n}}\Big)\Big).$$

This leads to our confidence interval for r_n, which is

$$\Big[\hat{r}_n\Big(1 - \frac{cz_{\alpha/2}}{\sqrt{n}}\Big),\ \hat{r}_n\Big(1 + \frac{cz_{\alpha/2}}{\sqrt{n}}\Big)\Big].$$

When we calculate this confidence interval from data, the random variables H_i that are used to obtain the estimate $\hat{r}_n$ only approximately have a logistic distribution. We have not proved the asymptotic validity of the confidence interval using this approximation, but the confidence interval performs well in simulations.

3.2 Confidence interval based on internal branch lengths

Recall that we estimate the growth rate r_n from the internal branch lengths by using

$$\hat{r}_n = \frac{n}{L_n^{in}}.$$

Let $0 < \alpha < 1$, and recall that $z_{\alpha/2}$ is the number such that if Z has a standard normal distribution, then $P(Z > z_{\alpha/2}) = \alpha/2$. By Theorem 1, we have

$$1-\alpha = \lim_{n\to\infty} \mathbb{P}\Big(-z_{\alpha/2} \le \frac{r_n}{\sqrt{n}}\Big(L_n^{in} - \frac{n}{r_n}\Big) \le z_{\alpha/2}\Big)$$
$$= \lim_{n\to\infty} \mathbb{P}\Big(\frac{n}{r_n}\Big(1 - \frac{z_{\alpha/2}}{\sqrt{n}}\Big) \le L_n^{in} \le \frac{n}{r_n}\Big(1 + \frac{z_{\alpha/2}}{\sqrt{n}}\Big)\Big).$$

Multiplying the inequality by r_n/L_n^{in}, we get

$$1-\alpha = \lim_{n\to\infty} \mathbb{P}\Big(\frac{n}{L_n^{in}}\Big(1 - \frac{z_{\alpha/2}}{\sqrt{n}}\Big) \le r_n \le \frac{n}{L_n^{in}}\Big(1 + \frac{z_{\alpha/2}}{\sqrt{n}}\Big)\Big)$$
$$= \lim_{n\to\infty} \mathbb{P}\Big(\hat{r}_n\Big(1 - \frac{z_{\alpha/2}}{\sqrt{n}}\Big) \le r_n \le \hat{r}_n\Big(1 + \frac{z_{\alpha/2}}{\sqrt{n}}\Big)\Big).$$

Therefore, an asymptotically valid $100(1-\alpha)\%$ confidence interval for r_n is given by

$$\left[\hat{r}_n\left(1-\frac{z_{\alpha/2}}{\sqrt{n}}\right),\ \hat{r}_n\left(1+\frac{z_{\alpha/2}}{\sqrt{n}}\right)\right].$$

3.3 Confidence interval based on shared mutations

Recall that, if the mutation rate ν_n and the site frequency spectrum are known, but we do not have a reconstruction of the entire tree, we can estimate the growth rate from the number of shared mutations using

$$\hat{r}_n = \frac{n\nu_n}{M_n^{in}}. \tag{69}$$

Recall that in the statement of Corollary 2, we defined

$$\sigma_n^2 = \frac{n\nu_n^2}{r_n^2}\left(1+\frac{r_n}{\nu_n}\right).$$

From Corollary 2, we get

$$\begin{aligned}
1-\alpha &= \lim_{n\to\infty}\mathbb{P}\left(-z_{\alpha/2} \le \frac{1}{\sigma_n}\left(M_n^{in} - \frac{n\nu_n}{r_n}\right) \le z_{\alpha/2}\right) \\
&= \lim_{n\to\infty}\mathbb{P}\left(\frac{n\nu_n}{r_n} - z_{\alpha/2}\sigma_n \le M_n^{in} \le \frac{n\nu_n}{r_n} + z_{\alpha/2}\sigma_n\right) \\
&= \lim_{n\to\infty}\mathbb{P}\left(\frac{n\nu_n}{r_n}\left(1-\frac{z_{\alpha/2}}{\sqrt{n}}\sqrt{1+\frac{r_n}{\nu_n}}\right) \le M_n^{in} \le \frac{n\nu_n}{r_n}\left(1+\frac{z_{\alpha/2}}{\sqrt{n}}\sqrt{1+\frac{r_n}{\nu_n}}\right)\right).
\end{aligned}$$

We now multiply the inequality by r_n/M_n to get

$$1-\alpha = \lim_{n\to\infty}\mathbb{P}\left(\hat{r}_n\left(1-\frac{z_{\alpha/2}}{\sqrt{n}}\sqrt{1+\frac{r_n}{\nu_n}}\right) \le r_n \le \hat{r}_n\left(1+\frac{z_{\alpha/2}}{\sqrt{n}}\sqrt{1+\frac{r_n}{\nu_n}}\right)\right).$$

Note that we can not use these upper and lower bounds as a confidence interval because they involve r_n, which is unknown. However, we can approximate r_n/ν_n under the square root by $\hat{r}_n/\nu_n$, which equals n/M_n^{in}. Because $\hat{r}_n/r_n$ converges in probability to 1 when (18) holds, an asymptotically valid $100(1-\alpha)\%$ confidence interval for r_n is given by

$$\left[\hat{r}_n\left(1-\frac{z_{\alpha/2}}{\sqrt{n}}\sqrt{1+\frac{n}{M_n^{in}}}\right),\ \hat{r}_n\left(1+\frac{z_{\alpha/2}}{\sqrt{n}}\sqrt{1+\frac{n}{M_n^{in}}}\right)\right].$$

3.4 Confidence interval for tumor age

Recall that when r_n is known, we can estimate the tumor age T_n by $\hat{T}_n$. By Corollary 3, when (20) holds, we have

$$\frac{1}{T_n}\sqrt{\frac{n\nu_n}{r_n+\nu_n}}\left(\hat{T}_n - T_n\right) \Rightarrow Z,$$

where Z has a standard normal distribution. Therefore,

$$\begin{aligned}1-\alpha &= \lim_{n\to\infty} \mathbb{P}\Big(- z_{\alpha/2} \le \frac{1}{T_n}\sqrt{\frac{n\nu_n}{r_n+\nu_n}}\left(\hat{T}_n - T_n\right) \le z_{\alpha/2}\Big) \\ &= \lim_{n\to\infty} \mathbb{P}\Big(T_n\Big(1 - z_{\alpha/2}\sqrt{\frac{r_n+\nu_n}{n\nu_n}}\Big) \le \hat{T}_n \le T_n\Big(1 + z_{\alpha/2}\sqrt{\frac{r_n+\nu_n}{n\nu_n}}\Big)\Big) \\ &= \lim_{n\to\infty} \mathbb{P}\left(\frac{\hat{T}_n}{1 + z_{\alpha/2}\sqrt{\frac{r_n+\nu_n}{n\nu_n}}} \le T_n \le \frac{\hat{T}_n}{1 - z_{\alpha/2}\sqrt{\frac{r_n+\nu_n}{n\nu_n}}}\right).\end{aligned}$$

The mutation rate ν_n is not assumed here to be known, but by rearranging (69) and using the result of Corollary 2, we see that ν_n can be estimated by

$$\hat{\nu}_n = \frac{r_n M_n^{in}}{n},$$

and then $\hat{\nu}_n/\nu_n$ converges in probability to 1. When we replace ν_n by $\hat{\nu}_n$, the square root in the expression above becomes

$$\sqrt{\frac{r_n+\hat{\nu}_n}{n\hat{\nu}_n}} = \frac{1}{\sqrt{n}}\sqrt{1+\frac{n}{M_n^{in}}}.$$

It follows that for $0 < \alpha < 1$, an asymptotically valid $100(1-\alpha)\%$ confidence interval for T_n can be obtained by

$$\left[\frac{\hat{T}_n}{1 + \frac{z_{\alpha/2}}{\sqrt{n}}\sqrt{1+\frac{n}{M_n^{in}}}}, \frac{\hat{T}_n}{1 - \frac{z_{\alpha/2}}{\sqrt{n}}\sqrt{1+\frac{n}{M_n^{in}}}}\right].$$

4 Supplementary Information - Data application

4.1 Agreement across methods for growth rate estimates

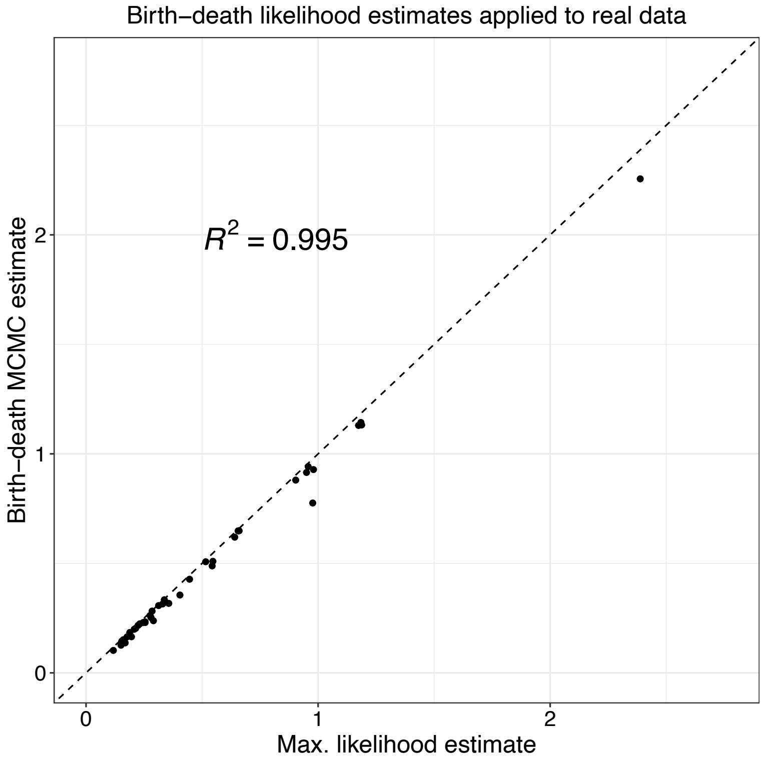

Supplementary Figure 2: **Maximum likelihood and birth-death MCMC agree on blood data:** There are a few cases where the maximum likelihood estimate is slightly higher than the birth-death MCMC, but the differences are generally very small. The agreement indicates that our assumptions of $n << N, T \to \infty$, and $n \to \infty$ do not significantly affect the estimates when applied to the data.

We see excellent agreement between the birth-death MCMC and our maximum likelihood estimates when applied to real data (Supp. Fig. 2). This motivated our decision to include only the maximum likelihood estimates in Application to human blood datasets for ease of illustration. The birth-death MCMC agreement with our maximum likelihood estimates provides validation that the approximations required to use the maximum likelihood estimate did not affect estimates in any significant way, at least in the context of this dataset.

While agreement between the maximum likelihood and internal lengths estimates is generally good when applied to the real data (Supp. Fig. 3), the internal lengths estimate tends to be higher than the maximum likelihood estimate in many cases. We hypothesize that this may be due to slight differences in the fitness of cells within the clone. While, on average, clones fit the neutral expectation (see Main text Fig. 5A), there may be cases where the random merging of lineages is violated to some degree. Slight fitness differences within a clone, leading to non-random lineage merging, could reduce the sum of internal lengths, leading to higher estimates of r, based on Main text Eq. 4. In order to test this, we re-generated trees by randomly merging the lineages using the coalescence times from the real data. In these re-generated trees (see Supp. Fig. 3B), the internal lengths estimates agreed more

closely with the maximum likelihood estimates, suggesting that slight differences in fitness within a clone may be responsible for the discrepancies that we see in the real data sets.

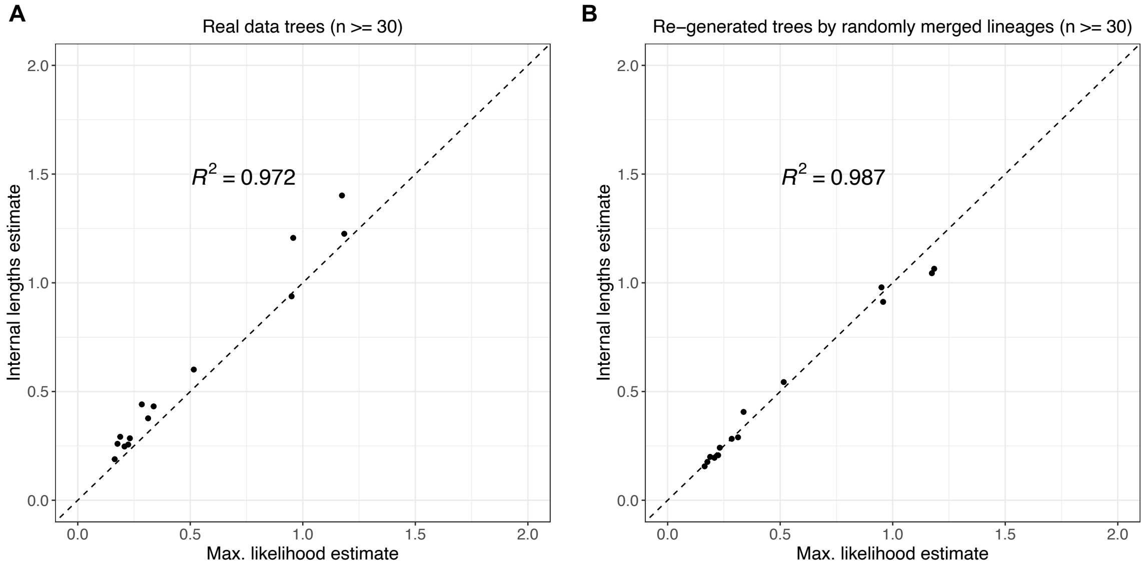

Supplementary Figure 3: **Maximum likelihood and internal lengths estimate. A:** Internal lengths estimates for some clones are higher than maximum likelihood estimates. **B:** When we re-generate trees by taking the coalescence times from the data and randomly merging the lineages, we see better agreement between internal lengths and maximum likelihood estimates. Note: we use only those real data trees with $n \geq 30$ sampled cells in this analysis, to ensure that the effect is less likely to be caused by chance.

4.2 Clonal fraction and Phylofit with ACF

As described by Williams et al., Phylofit is "an efficient MCMC approach that models selection/growth by directly fitting the three parameter deterministic phase population trajectory using the joint probability density of coalescence times given the population size trajectory"[25]. The three parameters mentioned are the growth rate, the total number of hematopoietic stem cells, and the midpoint time of a deterministic logistic growth population trajectory. The likelihood function for the coalescence times is based on Equation 1 in Lan et. al[26]. In this sense, it is similar to our approach, leveraging the information provided by coalescence times, but is based on the Kingman coalescent with deterministic growth rather than a stochastic growth process. There are two ways to run Phylofit, only one of which is directly comparable to our methods.

Phylofit optionally incorporates Aberrant Cell Fraction (ACF) into its calculation of the likelihood. Aberrant Cell Fraction is simply the number of sampled cells within a clone divided by the total number of sampled cells. ACF is an approximation of the clonal fraction at the time of sampling estimated from single cell-derived colonies, analogous to the variant allele frequency that would be observed in bulk whole genome sequencing. We refer to it alternatively as "sampled clonal fraction" in Main text section Application to human blood datasets, but use "Aberrant Cell Fraction" or "ACF" here for consistency with the terminology in Williams et al.[25]. When incorporating ACF, Phylofit assumes that the population trajectory of the clone is logistic, with a carrying capacity for the clone

equal to the total number of hematopoietic stem cells. While coalescence events provide information from the early history of the clone, when the total clone size is on the order of the sample size n, the ACF provides information on the clone size at the time of sampling. Therefore, while the assumptions for our methods simply require an early expansion phase with constant birth and death rates, Phylofit with ACF assumes a logistic population size trajectory with a carrying capacity indicating that a clone becomes completely dominant in the blood (ACF=1). This is a much stronger assumption than is necessary for our methods or when using Phylofit without incorporating ACF. If the clonally dominant logistic population size trajectory is not the actual population size trajectory of a clone, the estimated growth rate from Phylofit with ACF will be affected. Moreover, the population size of a clone at the time of sampling is likely affected by treatments if applied, competing expansions, and/or yet unknown reasons that carrying capacities would be lower than the total HSC pool.

In simulations where the growth trajectory is logistic with a carrying capacity equal to a clonal fraction of 1, Phylofit with ACF outperforms Phylofit without ACF as well as our methods (see Supp. Fig. 4). However, data[25,28,29] show that these Phylofit with ACF estimates are often discordant with early growth rate estimates. Williams et al. show that Phylofit produces significantly different estimates for growth rate depending on whether ACF is incorporated into the likelihood equation, suggesting that some clones show "smaller than expected final clonal fractions" (see Ext. Data Fig. 7c from Williams et al.[25]). Fabre et al.[29], who also include data from Mitchell et al.[28], compare their phylodynamic estimates using *phylodyn*[30] to the expected clone size later in life. Similar to Williams et al., they conclude that "at least for some clones and genes, the dynamics observed in later life are not representative of those that prevail earlier"[29]. Combining data from all three sources[25,28,29], we show in Supp. Fig. 5A that most clones do not reach the expected fraction that would be predicted by a logistic growth trajectory reaching a clonal fraction of 1. A similar analysis is shown in Figure 4d of Fabre et al.[29].

Our methods for estimating the net growth rate are derived from coalescent theory (specifically coalescent point processes), and estimate the growth rate during the early expansion phase of the clone. Because we estimate the growth rate from early coalescence times, our estimates reflect the growth rate during the time period when the number of cells in the clone is on the order of the sample size, n, which is typically between 10 and 100 in the data we have analyzed. Aberrant cell fraction (ACF) estimates, on the other hand, will be affected by the growth rate from clone initiation all the way until the sampling time. Note that ACF estimates are, by definition, only relevant for detectable population sizes above a certain VAF threshold, when calculating the fraction of cells within a given clone is possible using bulk sequencing. Therefore, ACF estimates are possible when the number of cells in the clone is on the order of N, the total number of hematopoietic stem cells, which is estimated to be between 25,000 and 300,000[31–33]. Both Fabre et al.[29] and Williams et al.[25] observed slower growth rates and reduced ACF at later timepoints than would be expected based on the early expansion. Using our early growth rate estimates, we confirm these conclusions, demonstrating that incorporating ACF leads to reduced growth rate estimates that do not agree with those produced by our methods or those from Phylofit without ACF. In order to compare our estimates to the early growth rate estimates from other methods, such as Phylofit[25], it is necessary to exclude ACF from the likelihood

A

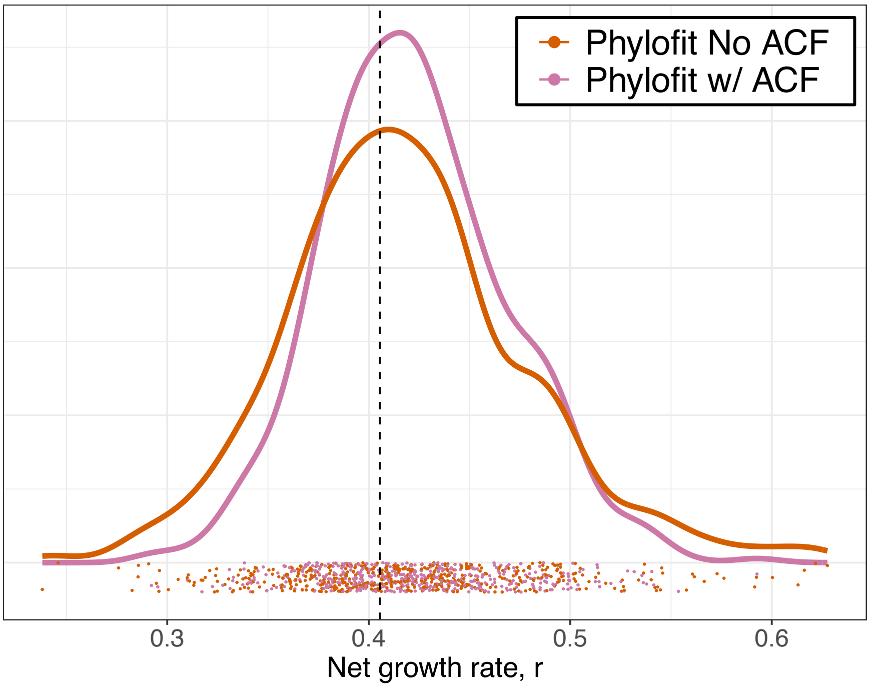

B

Method	RMSE	Mean Estimate	SD Estimate	True Growth Rate
Phylofit w/ ACF	0.049	0.423	0.046	0.405
Max. likelihood	0.060	0.423	0.057	0.405
Phylofit No ACF	0.062	0.418	0.060	0.405
Internal lengths	0.065	0.404	0.065	0.405

Supplementary Figure 4: **Incorporating aberrant cell fraction (ACF) leads to improved estimates on simulated data. A:** Estimated growth rates from Phylofit with (pink) and without (orange) ACF metric shows that ACF does give a slight advantage in the simulated case. Function *run_selection_sim()* from the *rsimpop* package[25] was used to simulate a logistic birth-death clonal expansion with growth rate $r = \log(1.5) = 0.405$ and a carrying capacity equal to a total HSC population size of $N = 100,000$. That is, we allowed the clone's carrying capacity to be equal to a clonal fraction (or ACF) of 1. The simulated clone was allowed to expand for 33 years following driver initiation, so that the expected population size was roughly 85% of the carrying capacity. $n = 100$ cells were then sampled to reconstruct the tree. The process was repeated 500 times. **B:** Table shows performance across methods for same simulations from (A), including our methods and ordered by root mean square error (RMSE).

calculation. Fortunately, Phylofit has been designed to run with or without ACF[25]. Once we remove ACF, we find similar estimates across methods (see Supp. Fig. 5B-C). Therefore, we compare our estimates to Phylofit without ACF, as this method, like our methods, estimates the growth rate during the expansion phase of the clone. However, published results in Williams et al.[25] consider ACF for growth rate inference, so our results using Phylofit differ from those which they present.

Possible explanations for the discordance between growth rates from early expansion and those using ACF are treatment effects, frequency dependent selection, and nested or independent clonal expan-

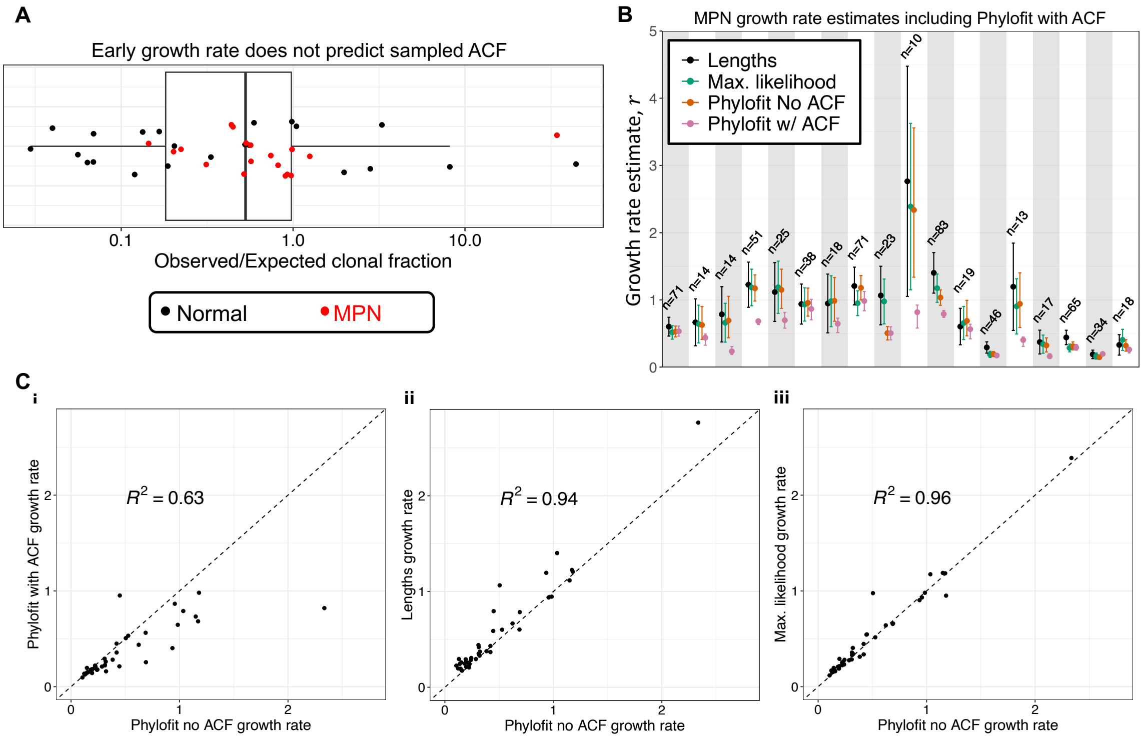

Supplementary Figure 5: **Early growth rate is not predicted by aberrant cell fraction (ACF). A:** Ratio of observed to expected clonal fraction assuming a logistic growth trajectory. There is a clear bias shown for clones which expand rapidly at first (high expected ACF) but decelerate (Observed ACF $<$ Expected ACF). Parameters used to calculate expected ACF are Phylofit (no ACF) growth rate and HSC carrying capacity $N = 100,000$. **B:** Growth rate estimates of clones from individuals with MPN shows that Phylofit with ACF is a low outlier in many cases. **C:** Correlation between Phylofit without ACF and Phylofit with ACF (i), internal lengths method (ii), and maximum likelihood method (iii) from estimates applied to hematopoietic clones. Phylofit with ACF is not capturing the same expansion rates estimated by other methods. Specifically, the correlation coefficient between Phylofit with ACF and Phylofit without ACF (i) is lowest of all the methods, with a clear bias towards lower growth rates estimated by the Phylofit with ACF method. Note that all hematopoietic clones included except the two clones from the Van Egeren dataset[27] are excluded from these Phylofit with ACF analyses because the cells were not sampled randomly.

sions which may outcompete the original clone. Clinical evidence suggests that some patients have relatively stable clonal fractions below 1 for extended periods of time[34]. Further, estimates of clonal fraction may be affected by sampled cell type (Peripheral blood vs. bone marrow and granulocytes vs. mononuclear cells vs. whole blood), as shown in Supp. Fig. 6. Van Egeren et al.[27] observe this in the context of *JAK2* mutant cells, which appear at higher frequency in erythroid progenitors. Such a lineage bias which may lead to clonal dominance in only a subset of the blood progenitors. Clonal fraction in whole blood may saturate below 0.5, even when a clone has become completely dominant within a specific type of progenitor (i.e. Megakaryocyte Erythroid Progenitors (MEP) in *JAK2* mutants). Therefore, the use of ACF in the estimation of early growth rates using coalescence times is prone to errors due to measuring difficulties and poorly understood growth trajectories of clones at high fraction. While measures of clonal fraction are valuable clinically[34], there is no single growth

rate metric that can currently be used to explain early coalescent and late clonal fraction data in most cases.

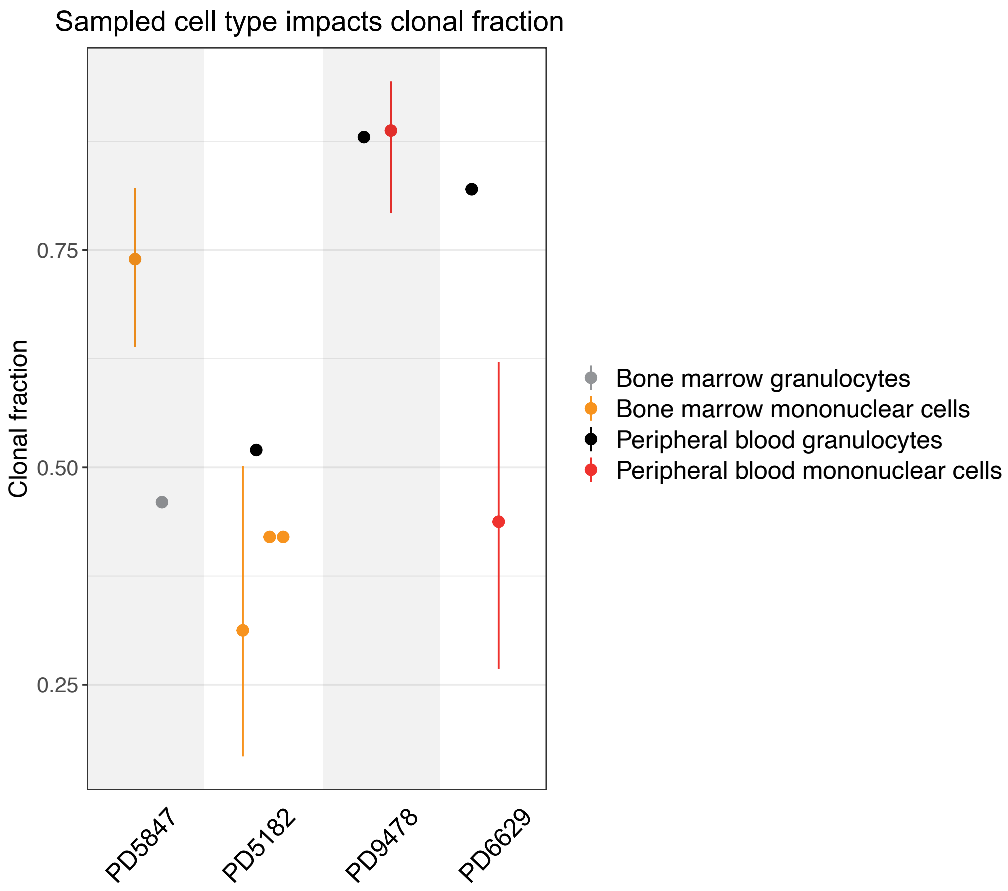

Supplementary Figure 6: **Clonal fraction estimates depend on sampled cell types.** In three out of four individuals with samples from different cell types taken within a month of each other, dominant clonal fraction is significantly different depending on the sampled cell type (all except PD9478). Estimates with error bars represent those taken from single cell colony data, with 95% confidence intervals based on binomial sampling. Points without error bars represent bulk recapture samples at mean depth $> 300\times$. All data from Williams et al.[25].

4.3 Longitudinal modeling

4.3.1 Longitudinal clone inclusion criteria

As noted in Main text section Longitudinal validation of clone growth estimates, we only use data for longitudinal validation that has multiple timepoints sampled from a single cell type, as sampled cell type may affect the estimated clonal fraction. The possible effects of sampled cell type are discussed in Supplementary section 4.2 and shown in Supp. Fig. 6. This presents obvious difficulties for longitudinal growth modeling, so we exclude such data.

In longitudinal data from individuals without hematological malignancies[29], sequential data typically shows a lower but increasing VAF, making it ideal for our estimates of early growth rate. However, in longitudinal data from individuals with MPN[25], increased clonal competition and treatment gives rise

to more complicated dynamics. Many clones which at first appear advantageous are outcompeted by clones with higher fitness or knocked down by treatment, leading to clones occasionally decreasing in Variant Allele Frequency (VAF) over the sampled time period. To avoid these external effects, which are not representative of the growth rate during the early expansion phase, we did not consider longitudinal data from clones decreasing in size. Any data points featuring more than a 20% decrease in VAF from a previous timepoint were removed. Removal due to a 20% drop was only applied if the previous VAF was ≥ 0.05, to avoid removing data due to small fluctuations at low VAF. After removing any decreasing data, we required 4 data points in total and at least 2 with a VAF > 0 and ≤ 0.25. Finally, because we are looking for expanding clones, we remove any clones which do not increase in VAF by at least 0.05.

This filtering leaves us with 60 clones, with 56 from Fabre et al.[29] and 4 from Williams et al.[25]. Of these, one clone from Williams et al.[25] and two clones from Fabre et al.[29] have sufficient matched single cell data to make growth rate estimates from both data modalities, and these are shown in Main text Figure 6A-D. We note that the only MPN clone with matched single-cell and longitudinal data (PD9478: *JAK2* + *DNMT3A*) is also from a patient that is essentially untreated, with the only intervention being venesection (bleeding). Further, the *JAK2* + *DNMT3A* clone in this patient appears to be the only large expansion, and there are 11 longitudinal samples from the same cell type, making this clone an ideal candidate for validation.

From the remaining 57 clones without matched single cell data, we identified 17 clones with mutations in the same driver gene as a single cell clone. We then excluded the longitudinal data from two MPN clones from Williams et al.[25], as treatment likely affected the VAF in both cases. Further, we removed one *JAK2* clone where there was a competing clone at high VAF, as this was likely to affect the growth rate. After fitting the logistic growth rate model to each of the remaining clones, as detailed below in Supplementary section 4.3.2, we removed one DNMT3A clone where the fit failed to converge. After all filtering steps, there were 13 longitudinal clones matching to 10 single cell clones. *DNMT3A* mutant clones were the most abundant, with 7 *DNMT3A* clones having longitudinal data and 5 having single cell data. There were 3 longitudinal *JAK2* clones and 4 single cell *JAK2* clones. There were 3 longitudinal *PPM1D* clones and 1 single cell *PPM1D* clone. This data is shown in Main text Figure 6E.

4.3.2 Logistic modeling of longitudinal data

We used the *nls()* function from the *stats* package in R, with the port algorithm, to perform fitting to the following logistic growth equation, which models the VAF over time:

$$\mathrm{VAF}(t) = \frac{K}{1 + e^{-(rt+\phi)}} \tag{70}$$

Bounds for K, representing the carrying capacity, are $[0, 0.5]$, as all mutations are in diploid regions. Bounds for r are $[0, 5]$ per year, and bounds for ϕ are $[-500, 0]$. 95% confidence intervals are found by assuming normality of the parameter estimate, using $r \pm 1.96 * \mathrm{stdError}$ to calculate the bounds.

Midpoint time of the logistic curve is given by $t_m = -\phi/r$.

Importantly, we do not assume a carrying capacity K equal to a variant allele frequency of 0.5, instead allowing the carrying capacity to be fit simultaneously with the growth rate and midpoint time. This is discussed in Supplementary section 4.2. Our decision to fit K rather than fixing it at 0.5 is motivated by data[29] showing growth rates slowing more than would be expected by a logistic fit with $K = 0.5$, even in the absence of treatment. In fitting to longitudinal data from 16 clones (3 from Figure 6A-C and 13 from Figure 6E), nine clones have a fit VAF carrying capacity below 0.4, consistent with the claim that clones do not always saturate at an allele frequency of 0.5. In fact, single cell clonal fraction data from the 13 MPN patients we have analyzed shows no example of a somatic clone that is completely dominant, despite matched stromal[27] or buccal[25] normal cell samples distinguishing between somatic and germline variants.

It should be noted that a logistic growth model with any carrying capacity may not be the most appropriate model for clones in the blood, especially in the presence of other clones and/or treatment[35]. Our coalescent methods avoid this dependence on a particular model by assuming only that exponential growth occurs immediately following the initiation of a clone, while its population size is still on the order of the sample size, n. However, longitudinal validation requires the choice of a particular growth model in order to estimate a growth rate. Based on previous work exploring practical parameter identifiability in sigmoid growth models[36], the logistic model outperformed Gompertz and Richards' models, which is why we use it for longitudinal validation.

4.4 Annotating Clones

As noted in the main text, "early mergers" are instances which will affect our methods significantly. For an example of an early merger occurring in a simulated tree, see Supp. Fig. 7A. Such an early merge in the ancestry of two sampled cells leads to a sum of internal lengths much greater than would otherwise be expected. Also, the distribution of coalescence times would now have an outlier. Thus, both the internal lengths method and the method of maximum likelihood would be affected, and the resulting growth rate estimate from each method would be lower than the true value.

In the context of hematopoiesis, there are several possible explanations for why an early merger might occur. First, there may be an expansion within a clone due to a cell within that clone acquiring an additional fitness advantage. We call this a "nested expansion". In Supp. Fig. 7B, we see that the *DNMT3A* clone has two such expansions, one resulting from a *CBL* mutation, and the other resulting from a *JAK2* mutation. While these expansions are a result of known driver mutations and have more sampled cells, it is possible for a recent or slow growing expansion to only be captured by two sampled cells. Further, it is possible for the expansion to be caused by an unknown driver mutation or epigenetic change, complicating the annotation of a such a nested expansion.

Second, the early merger can be a result of chance. While unlikely, the sampling may capture two cells which have a recent common ancestor. If stem cell turnover independent of further clonal expansions is high or the clone population size is low, sampled cells are more likely to have recent common

ancestors. This probability will be influenced by the number of total hematopoietic stem cells (HSCs) as well as the normal turnover rate. Fewer stem cells and higher turnover increase the probability of early mergers, consistent with coalescent theory in critical branching processes[3].

In both of these cases, estimating the main or parent clone will be more accurate if the early merger is removed from the phylogenetic tree. In the dataset we analyze, there were no clear instances of early mergers without annotated drivers. That is, the case of Supp. Fig. 7A did not occur. However, many cases similar to Supp. Fig. 7B occur, where a larger nested clone or one with a known driver is present. In those cases, we remove the nested expansion(s) and estimate the growth rate using the remaining samples. If the nested expansion has 10 or more samples, we also estimate the growth rate of the nested clone independently.

Supp. Fig. 7B shows an example of manual annotation of clones. Most of this work was performed in the papers that generated the data[25,27–29]. However, there are cases where our approach differs slightly from the annotation in those works. For example, as shown in Supp. Fig. 7B, we include an expanded clone with an unknown driver which is not included in the work of Williams et al.[25]. Other expanded clones without known drivers are annotated by the authors in the work of Fabre et al.[29] and Mitchell et al.[28] and are not present in the data from Van Egeren et al.[27], so they do not lead to differences in clonal annotation between our work and these three papers. Further, in cases where nested expansions occur, we remove the expansions from the tree that is ultimately used for our estimates, leaving only one resulting tip (sampled cell) from each nested expansion, so as to preserve the original coalescence event preceding the acquisition of an added fitness advantage. It is unclear whether this step is performed in the previous analyses[25,28,29]. Nested expansions do not appear in the data from Van Egeren et al.[27]. Because we use the same tree and clone annotations when running Phylofit and our methods, these differences do not affect the consistency across methods. Many sources are available for tree reconstruction and further work will be required to automate the process of annotating clones and identifying nested clones. Such work will likely require metrics of tree balance in addition to coalescent theory. For the time being, manual annotation of clones is the best option, and we attempted to use the most accurate trees as input.

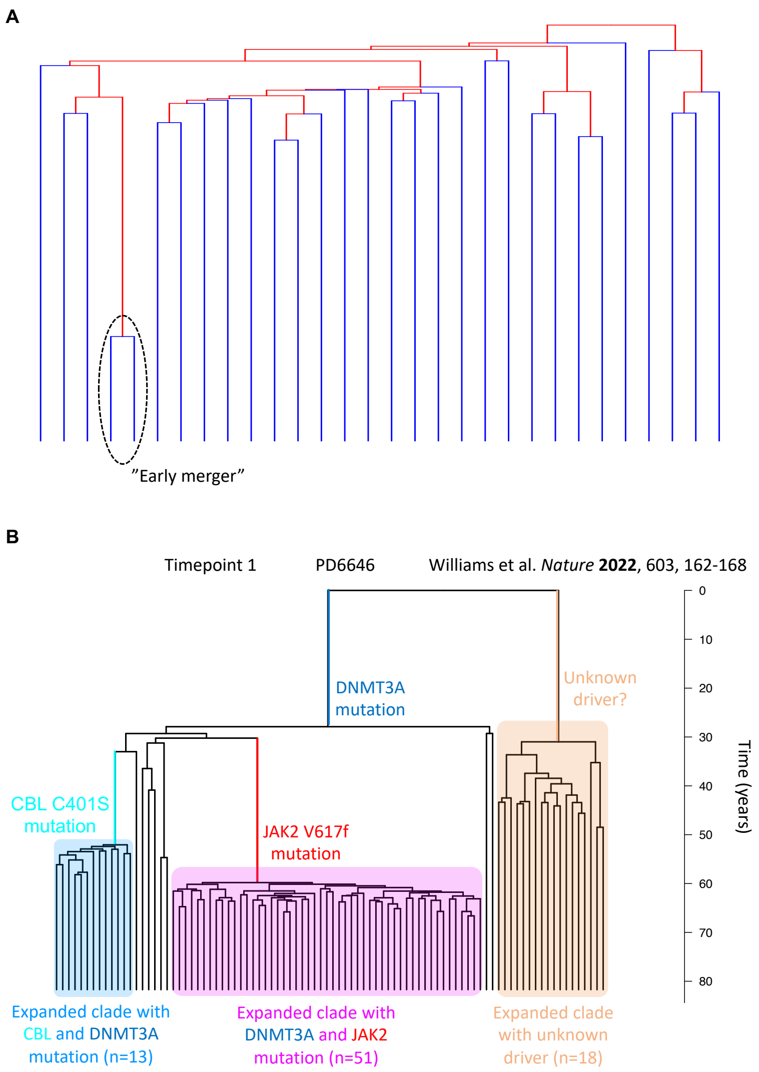

Supplementary Figure 7: **Example trees. A:** An example of an early merger which would make our methods inaccurate. Excess internal lengths which is a result of a possible nested expansion and/or normal stem cell turnover would lead to a smaller growth rate than would otherwise be estimated without such an early merger. Similarly, maximum likelihood estimate using the distribution of coalescence times would lead to a smaller growth rate. If the merger is caused by a known driver or thought to be a nested expansion it can be removed. However, it may be difficult to decide whether to remove the early merger in cases where there is no clear driver. **B:** A real data example of a reconstructed phylogenetic tree from Williams et al.[25] with clades with greater than 10 sampled cells annotated. Each tip of the tree represents a sampled cell. Here, we see a *DNMT3A* mutation leading to nested clones, one with a *CBL* mutation and one with a *JAK2* mutation. This tree also shows a clonal expansion with an unknown driver, and we estimated a growth rate for this clone.

5 Death rate to birth rate ratio

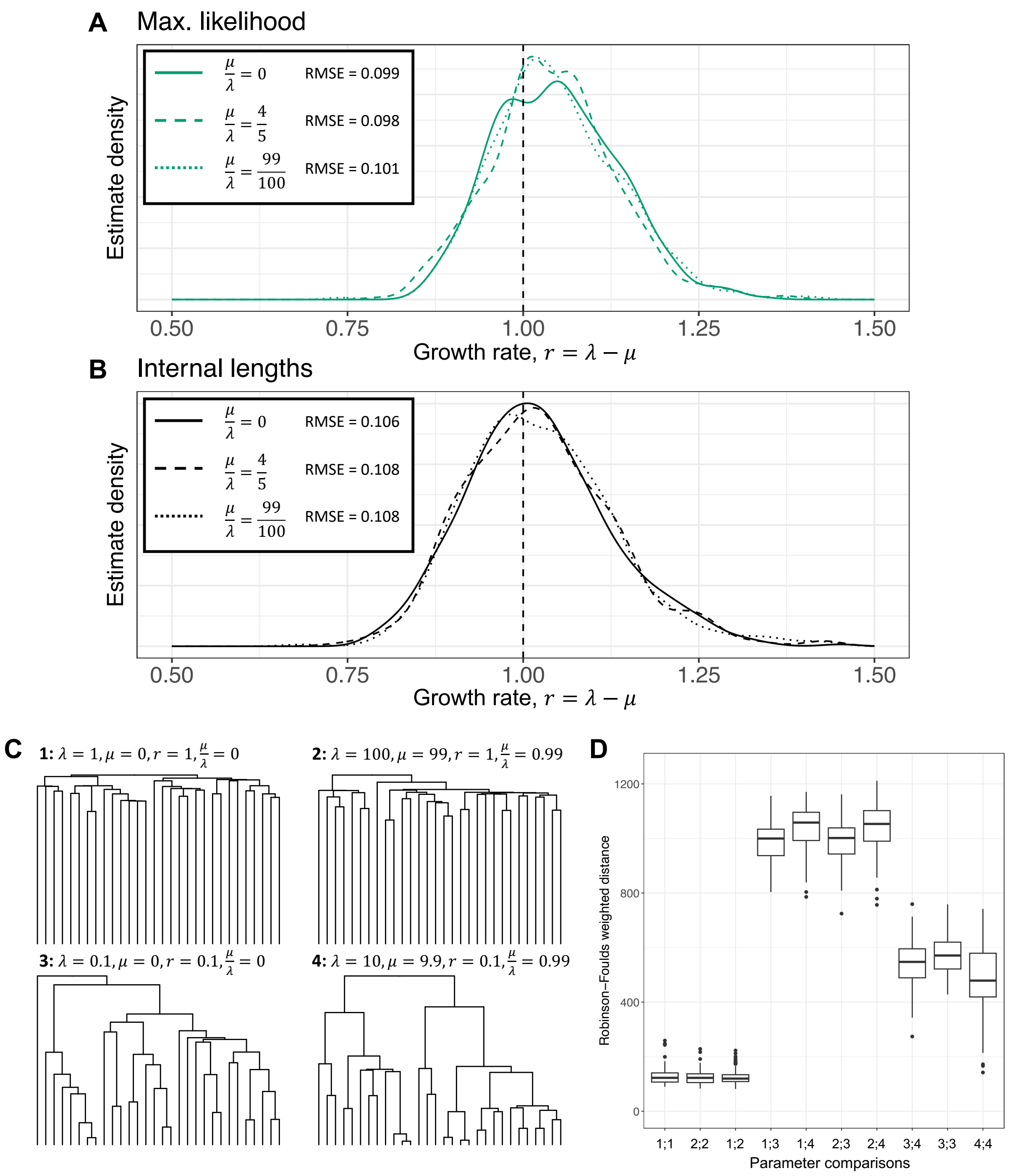

Supplementary Figure 8: **For fixed T, growth rate determines tree shape.** Simulations with growth rate $r = 1$ and differing birth rates (λ) and death rates (μ) show that growth rate estimates made by our methods are valid for any ratio of μ/λ. Parameters corresponding to each different ratio: Solid line: $\lambda = 1, \mu = 0$. Dashed line: $\lambda = 5, \mu = 4$. Dotted line: $\lambda = 100, \mu = 99$. Other parameters: $n = 100$ sampled cells and $T = 40$. 500 trees simulated for each ratio of μ/λ and each method, **A:** Maximum likelihood method, **B:** Internal lengths method. **C:** Four simulated trees with various growth rates, r, and μ/λ ratios demonstrate that, for fixed T, growth rate $r = \lambda - \mu$ determines tree shape. $T = 40$ and $n = 30$ in trees labeled **1-4**. **D:** To quantify the similarity between phylogenetic trees produced by parameters shown in **C**, we computed Robinson-Foulds weighted distance[37] for 100 pairwise comparisons between randomly generated trees with either the same parameters used (**X;X**, i.e., **X** vs **X**) or 2 different sets of parameters used (**X;Y**). Trees with same growth rates and differing μ/λ ratios (**1;2** and **3;4**, with numbers **1-4** as labeled in **C**) were roughly as similar to each other as those with exact same parameters (**1;1, 2;2, 3;3, 4;4**). Those with differing growth rates were more dissimilar (**1;3, 1;4, 2;3**, and **2;4**).

6 Comparing results to Chronic Lymphocytic Leukemia (CLL)

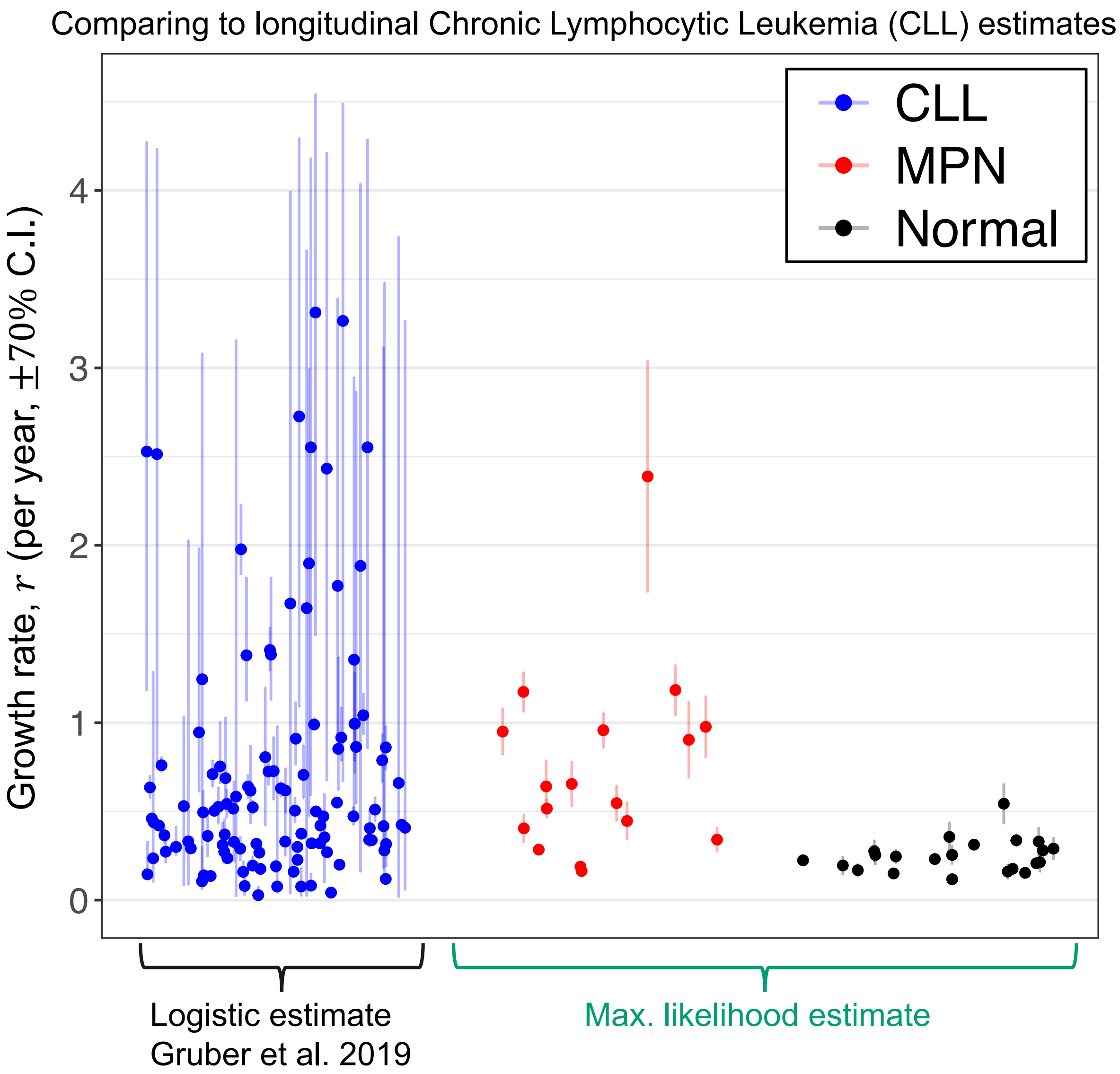

Supplementary Figure 9: **Longitudinal estimates from Chronic lymphocytic leukemia (CLL):** Longitudinal data of white blood cell (WBC) counts from 110 individuals with CLL were fit to logistic growth trajectories (including the possibility of exponential growth) in Gruber et al. 2019. Resulting growth rate estimates of CLL (blue; data from Supp. Tables 1 and 8 in[38]) were similar to our estimates of clone-specific growth rates in MPNs (red), and somewhat larger than our estimates for normal hematopoietic clones (black). Specifically, MPN clones had a median growth rate of 0.64 per year (interquartile range (IQR)= $0.40 - 0.96$) compared to a median CLL growth rate of 0.50 (IQR= $0.30 - 0.86$). Normal clones estimated by our methods had a median growth rate of 0.24 (IQR= $0.18 - 0.29$).

References

[1] Lambert, A. The coalescent of a sample from a binary branching process. *Theoretical Population Biology* **2018**, *122*, 30–35.

[2] Popovic, L. Asymptotic genealogy of a critical branching process. *The Annals of Applied Probability* **2004**, *14*, 2120–2148.

[3] Aldous, D.; Popovic, L. A critical branching process model for biodiversity. *Advances in Applied Probability* **2005**, *37*, 1094–1115.

[4] Stadler, T. On incomplete sampling under birth–death models and connections to the sampling-based coalescent. *Journal of Theoretical Biology* **2009**, *261*, 58–66.

[5] Lambert, A.; Stadler, T. Birth–death models and coalescent point processes: The shape and probability of reconstructed phylogenies. *Theoretical Population Biology* **2013**, *90*, 113–128.

[6] Harris, S. C.; Johnston, S. G.; Roberts, M. I. The coalescent structure of continuous-time Galton–Watson trees. *The Annals of Applied Probability* **2020**, *30*, 1368–1414.

[7] Cheek, D. The coalescent tree of a Markov branching process with generalised logistic growth. *Journal of Mathematical Biology* **2022**, *84*.

[8] Lambert, A. The allelic partition for coalescent point processes. *Markov Processes and Related Fields* **2009**, *15*, 359–386.

[9] Champagnat, N.; Lambert, A. Splitting trees with neutral Poissonian mutations I: Small families. *Stochastic Processes and their Applications* **2012**, *122*, 1003–1033.

[10] Delaporte, C.; Achaz, G.; Lambert, A. Mutational pattern of a sample from a critical branching population. *Journal of Mathematical Biology* **2016**, *73*, 627–664.

[11] Champagnat, N.; Henry, B. Moments of the frequency spectrum of a splitting tree with neutral Poissonian mutations. *Electronic Journal of Probability* **2016**, *21*, 1–34.

[12] Dinh, K. N.; Jaksik, R.; Kimmel, M.; Lambert, A.; Tavaré, S. Statistical Inference for the Evolutionary History of Cancer Genomes. *Statistical Science* **2020**, *35*, 129–144.

[13] Dahmer, I.; Kersting, G. The internal branch lengths of the Kingman coalescent. *Annals of Applied Probability* **2015**, *25*, 1325–1348.

[14] Dahmer, I.; Kersting, G.; Wakolbinger, A. The total external branch length of beta-coalescents. *Combinatorics, Probability, and Computing* **2015**, *23*, 1010–1027.

[15] Birkner, M.; Dahmer, I.; Diehl, C.; Kersting, G. The joint fluctuations of the lengths of the Beta$(2-\alpha,\alpha)$-coalescents. 2020; ArXiv Preprint 2009.13642.

[16] Disanto, F.; Fuchs, M. Distribution of external branch lengths in Yule trees. 2022; ArXiv Preprint 2208.04804.

[17] Durrett, R. Population genetics of neutral mutations in exponentially growing cancer cell populations. *Annals of Applied Probability* **2013**, *23*, 230.

[18] Johnston, S. G. The genealogy of Galton-Watson trees. *Electronic Journal of Probability* **2019**, *24*, 1–35.

[19] Thorisson, H. *Coupling, Stationarity, and Regeneration*; Springer: New York, 2000.

[20] Balakrishnan, N. *Handbook of the Logistic Distribution*; Marcel Dekker: New York, 1992.

[21] Diananda, P. The central limit theorem for m-dependent variables. Mathematical Proceedings of the Cambridge Philosophical Society. 1955; pp 92–95.

[22] Durrett, R. *Probability: Theory and Examples*, 5th ed.; Cambridge University Press: Cambridge, 2019.

[23] Sethuraman, J. Some Limit Theorems for Joint Distributions. *The Indian Journal of Statistics, Series A* **1961**, *23*, 379–386.

[24] Antle, C.; Klimko, L.; Harkness, W. Confidence Intervals for the Parameters of the Logistic Distribution. *Biometrika* **1970**, *57*, 397–402.

[25] Williams, N.; Lee, J.; Mitchell, E.; Moore, L.; Baxter, E. J.; Hewinson, J.; Dawson, K. J.; Menzies, A.; Godfrey, A. L.; Green, A. R., et al. Life histories of myeloproliferative neoplasms inferred from phylogenies. *Nature* **2022**, *602*, 162–168.

[26] Lan, S.; Palacios, J. A.; Karcher, M.; Minin, V. N.; Shahbaba, B. An efficient Bayesian inference framework for coalescent-based nonparametric phylodynamics. *Bioinformatics* **2015**, *31*, 3282–3289.

[27] Van Egeren, D.; Escabi, J.; Nguyen, M.; Liu, S.; Reilly, C. R.; Patel, S.; Kamaz, B.; Kalyva, M.; DeAngelo, D. J.; Galinsky, I., et al. Reconstructing the lineage histories and differentiation trajectories of individual cancer cells in myeloproliferative neoplasms. *Cell stem cell* **2021**, *28*, 514–523.

[28] Mitchell, E.; Spencer Chapman, M.; Williams, N.; Dawson, K. J.; Mende, N.; Calderbank, E. F.; Jung, H.; Mitchell, T.; Coorens, T. H.; Spencer, D. H., et al. Clonal dynamics of haematopoiesis across the human lifespan. *Nature* **2022**, 1–8.

[29] Fabre, M. A.; de Almeida, J. G.; Fiorillo, E.; Mitchell, E.; Damaskou, A.; Rak, J.; Orrù, V.; Marongiu, M.; Chapman, M. S.; Vijayabaskar, M., et al. The longitudinal dynamics and natural history of clonal haematopoiesis. *Nature* **2022**, 1–8.

[30] Karcher, M. D.; Palacios, J. A.; Lan, S.; Minin, V. N. phylodyn: an R package for phylodynamic simulation and inference. *Molecular ecology resources* **2017**, *17*, 96–100.

[31] Watson, C. J.; Papula, A.; Poon, G. Y.; Wong, W. H.; Young, A. L.; Druley, T. E.; Fisher, D. S.; Blundell, J. R. The evolutionary dynamics and fitness landscape of clonal hematopoiesis. *Science* **2020**, *367*, 1449–1454.

[32] Lee-Six, H.; Øbro, N. F.; Shepherd, M. S.; Grossmann, S.; Dawson, K.; Belmonte, M.; Osborne, R. J.; Huntly, B. J.; Martincorena, I.; Anderson, E., et al. Population dynamics of normal human blood inferred from somatic mutations. *Nature* **2018**, *561*, 473–478.

[33] Moeller, M. E.; Pere, N. V. M.; Werner, B.; Huang, W. Measures of genetic diversification in somatic tissues at bulk and single cell resolution. *bioRxiv* **2022**,

[34] Steensma, D. P. Clinical consequences of clonal hematopoiesis of indeterminate potential. *Hematology 2014, the American Society of Hematology Education Program Book* **2018**, *2018*, 264–269.

[35] Bolton, K. L.; Ptashkin, R. N.; Gao, T.; Braunstein, L.; Devlin, S. M.; Kelly, D.; Patel, M.; Berthon, A.; Syed, A.; Yabe, M., et al. Cancer therapy shapes the fitness landscape of clonal hematopoiesis. *Nature genetics* **2020**, *52*, 1219–1226.

[36] Simpson, M. J.; Browning, A. P.; Warne, D. J.; Maclaren, O. J.; Baker, R. E. Parameter identifiability and model selection for sigmoid population growth models. *Journal of theoretical biology* **2022**, *535*, 110998.

[37] Robinson, D. F.; Foulds, L. R. Comparison of phylogenetic trees. *Mathematical biosciences* **1981**, *53*, 131–147.

[38] Gruber, M.; Bozic, I.; Leshchiner, I.; Livitz, D.; Stevenson, K.; Rassenti, L.; Rosebrock, D.; Taylor-Weiner, A.; Olive, O.; Goyetche, R., et al. Growth dynamics in naturally progressing chronic lymphocytic leukaemia. *Nature* **2019**, *570*, 474–479.
